# Multimodal imaging to analyze the biomechanical properties of kidney tumors, evaluating feasibility, inter-modality correspondence, and diagnostic value (UroCCR-115)

**DOI:** 10.1371/journal.pone.0351477

**Published:** 2026-07-08

**Authors:** Mattia Ronca, Paolo Conforti, Amandine Crombé, Manon Jaffredo, Solène Ricard, Thierry Colin, Mokrane Yacoub, Yann Le Bras, Jean-Christophe Bernhard, Gaelle Margue, Eva Fourage-Jambon

**Affiliations:** 1 Urology Department, Bordeaux University Hospital, Bordeaux, France; 2 I, CaRe Bordeaux-BRIC Inserm U1312, Bordeaux, France; 3 Service d’imagerie Médicale Adulte, Hôpital Pellegrin, Centre Hospitalier Universitaire de Bordeaux, Bordeaux, France; 4 Sarcotarget -BRIC Inserm U1312, Bordeaux, France; 5 SOPHiA GENETICS, Multimodal R&D Team, Pessac, France; 6 Department of Pathology, Bordeaux University Hospital, Bordeaux, France; PLOS: Public Library of Science, UNITED KINGDOM OF GREAT BRITAIN AND NORTHERN IRELAND

## Abstract

**Introduction:**

Assessment of renal tissue and renal tumor stiffness may provide complementary information for tissue characterization; however, conventional imaging modalities such as multiphasic computed tomography (CT) do not directly quantify biomechanical properties. Elastography techniques, including magnetic resonance elastography (MRE) and ultrasound elastography (US-E), allow noninvasive measurement of tissue stiffness but are not routinely available in standard clinical practice. This study protocol aims to develop a CT-based stiffness mapping of renal parenchyma and renal tumors by investigating the relationship between CT attenuation values and elastography-derived stiffness measurements, using MRE and US-E as reference modalities.

**Methods and analysis:**

This monocentric, prospective, exploratory, non-randomized, and non-blinded diagnostic study will enroll 50 adults undergoing partial or radical nephrectomy for renal tumors at the University Hospital of Bordeaux. All participants will undergo a predefined multimodal imaging protocol—including contrast-enhanced CT, multiparametric magnetic resonance imaging (MRI) with -MRE and US-E—conducted between inclusion and the day before surgery. The primary objective is to construct a regression model predicting MRE-derived elasticity (μMRE) from CT density values using multiple machine-learning algorithms evaluated through repeated nested cross-validation. Secondary analyses will include voxel-level and region-of-interest correlations across modalities, feasibility and image-quality assessment of DWI-vMRE, repeatability of elastography measurements, identification of limiting factors such as BMI, sarcopenia, lesion location and architecture, evaluation of inter-modality de-correlation and associations with final histopathology (including subtype and grade).

**Trial registration:**

ClinicalTrials.gov identifier: NCT06525831. Protocol ID-RCB: 2024-A00959-38. Recruitment began on 7 March 2025.

## Introduction

Renal cell carcinoma (RCC) accounts for approximately 3–5% of all adult malignancies and represents the seventh leading cause of cancer-related mortality in men and the tenth in women [1]. More than 50% of RCCs are discovered incidentally during imaging performed for unrelated purposes [2]. Medical imaging plays a central role in the diagnostic and therapeutic management of RCC. It enables confirmation and localization of renal lesions, assessment of malignancy and histological subtype, TNM staging, surgical planning, biopsy guidance, treatment monitoring, and detection of recurrence. Ultrasound (US), CT and magnetic resonance imaging (MRI) are complementary modalities in this setting, with CT widely used for three-dimensional reconstruction and preoperative modeling. However, the relationships among renal tissue density on multiphasic CT, enhancement characteristics, and tissue stiffness measured through US and MRI remain insufficiently defined. Accurate estimation of renal tissue stiffness from imaging could expand the diagnostic capabilities of CT and improve the anatomical and mechanical fidelity of 3D models used for surgical planning. Multiparametric magnetic resonance imaging (mpMRI) is currently the most advanced imaging modality for renal tumor characterization and for differentiating histological subtypes. However, its diagnostic performance remains insufficient to reliably obviate invasive biopsy procedures when tumor malignancy is not clearly

A meta-analysis including 1,239 solid renal lesions reported an overall correct classification rate of 64%, with sensitivity of 95% and specificity of 63% [3]. In another multicenter prospective study of 250 small renal masses (<4 cm), mpMRI yielded a sensitivity and specificity of 75% and 79%, respectively, for diagnosing clear cell RCC (ccRCC), with modest interobserver agreement (κ = 0.58) [4]. As a result, approximately 30% of partial or radical nephrectomies are performed for benign lesions—most commonly fat-poor angiomyolipomas and oncocytomas—leading to avoidable morbidity, including hemorrhage, fistula, and infection in about 16% of cases [2,5]. mpMRI provides the most comprehensive integration of morphological and functional parameters for renal tumor characterization. By combining T1- and T2-weighted sequences, chemical-shift imaging, dynamic contrast-enhanced imaging, and diffusion-weighted imaging, mpMRI offers richer insights into tumor phenotype compared with CT [3]. However, even with machine-learning–based predictive models, diagnostic reproducibility and clinical reliability remain suboptimal [3,6–8]. Beyond morphological evaluation, MRI allows quantitative assessment of biomechanical properties through MRE. Preliminary studies have shown strong correlations between renal parenchymal stiffness and histopathological features such as tubulo-interstitial fibrosis, extracellular matrix expansion, and glomerulosclerosis [9,10], as well as associations between intratumoral elasticity and certain histological subtypes [11]. Prezzi et al. included 18 patients with renal tumors measuring 2–5 cm. MRE demonstrated superior discrimination between histological subtypes compared with other individual MRI sequences, including T2-weighted imaging, diffusion-weighted imaging, and dynamic contrast-enhanced imaging, particularly in differentiating clear cell RCC from oncocytoma (p = 0.007) These findings support the emerging diagnostic value of biomechanical imaging biomarkers. CT remains the reference modality for anatomical segmentation and 3D model construction because of its robustness and accessibility; however, no established relationship currently exists between CT density values and tissue stiffness derived from elastography. Establishing these correlations could enhance CT predictive value and improve the realism of 3D-printed kidney models [12–15].

## Materials and methods

### Aim

The following protocol tries to evaluate the concordance of advanced imaging techniques in assessing renal tissue stiffness for preoperative evaluation of renal tumors in patients undergoing a partial or radical nephrectomy for renal tumor at the Centre Hospitalier Universitaire Bordeaux. This protocol includes multiphasic CT, MRE and US-E, performed between inclusion and the day before surgery.

### Trial

This is a monocentric, prospective, explorator, non-randomized, and non-blinded diagnostic study. Patients enrolled are prospectively followed from inclusion to surgery, with all imaging procedures performed within a predefined preoperative window. All participants undergo a predefined multimodal imaging workflow including multiple diagnostic modalities, incorporing contrast-enhanced CT, mpMRI (including MRE) and US-E, aiming to explore the diagnostic potential of these techniques in the characterization of renal tumors and renal-parenchyma.

### Ethic declarations

The protocol number is ID-RCB 2024-A00959-38, version 2.0 from 16/10/2025. The study received regulatory approval from the Comité de Protection des Personnes (CPP) ÎdF1 and authorization from the ANSM. Patient recruitment began on 7 March 2025, and enrolment is planned over an 18-month period, followed by a 4-month completion phase for final assessments.The trial is registered on ClinicalTrials.gov with the reference NCT06525831 (Study Details | NCT06525831 | Rein 3D PRINT MECHANICS | ClinicalTrials.gov). All study procedures are conducted before surgery at the University Hospital of Bordeaux.

In accordance with French Law No. 2002-303 of March 4, 2002, participants were informed of their right to access the overall results of the study upon request.

### Study population

The study will enroll 50 patients with renal tumors referred for surgical management at the University Hospital of Bordeaux. Patients eligible for inclusion must be at least 18 years old or older, scheduled for nephrectomy for renal neoplasm within the Urology Department, and have a planned or available multiphasic CT as part of their preoperative workup. Participants must also provide informed consent for inclusion in the UroCCR cohort and participation in the Rein-3D Print Mechanics study, with the condition that they are affiliated with social security system. Exclusion criteria include pregnancy or breastfeeding, contraindications to MRI or gadolinium-based contrast agents, previous renal biopsy <15 days before MRI, and certain comorbidities such as obesity (body mass index (BMI) ≥ 30 kg/m²), thoracolumbar spinal fixation, or the presence of small (<2 cm) cystic or necrotic renal tumors with a solid component. Additionally, patients with ascites, legal protection measures, or difficulty in understanding and expressing themselves in French will be excluded.

All study data will be recorded in the imaging systems (Carestream PACS; Philips) and UroCCR database, managed by the CREDIM (Centre de Recherche et Développement en Informatique Médicale) at the University Hospital of Bordeaux. The database is authorized by the French Data Protection Authority (CNIL; authorization DR-2013–206, request n°912578) and operates under the national MR-001. The study involves senior urologists, expertised radiologists from the uro-vascular imaging department, the reference pathologist, biomedical engineers, data managers and biostatisticians.

Data entry was performed by trained personnel, and clinical variables were coded using standard dictionaries (MedDRA, ATC). Data quality was ensured through automated checks and query resolution. All data were pseudonymized, securely stored, and transferred via a protected platform when required. Confidentiality was maintained in accordance with regulatory standards, and study data were archived for 20 years.

### Study setting

This study aims to investigate the relationships between renal tumor biomechanical properties measured by multimodal imaging and underlying tumor characteristics, with the broader objective of improving non‑invasive diagnostic approaches. The study will enroll 50 patients scheduled for nephrectomy over an 18‑month period, with follow‑up extending 4 months after inclusion. To date, 40 patients have been enrolled, and recruitment is expected to be completed by September 2026. Approximately 250 patients per year are referred to the Department of Urology for the initial evaluation of renal lesions, establishing a robust recruitment base that is further strengthened by their established clinical and research collaborations through multidisciplinary tumor board meetings. Eligible patients will be recruited during urology consultations at Pellegrin Hospital or after identification by radiologists during imaging sessions. All participants will provide written informed consent in accordance with the approved protocol. Preoperative imaging will include ultrasound elastography, mpMRI (including MRE), and contrast‑enhanced CT, all performed within a standardized preoperative window to ensure methodological consistency. These imaging procedures will take place at Saint‑André Hospital for MRI and at Pellegrin Hospital for ultrasound examinations, using standardized equipment and performed by senior radiologists, with routine care multiphasic CT incorporated as clinically indicated. After the nephrectomy pathological analyses will follow standard institutional workflows. Imaging data will be analyzed at the voxel level for MRI and CT, and at the region of interest (ROI) level for US-E. All data will be managed securely according to protocol specifications until completion following the availability of the final histopathological report.

### Procedure

Each patient meeting the eligibility criteria will be enrolled during a dedicated inclusion visit (T0). During this visit, the investigator provides complete study information, verifies all inclusion and exclusion criteria, and obtains written informed consent before any research-related procedure is performed. Women of childbearing potential undergo a urine pregnancy test; a positive result leads to exclusion. As part of routine care, a contrast-enhanced multiphasic -CT will be performed if not already available at inclusion.

Between inclusion and surgery, all imaging examinations related to the study (T1) will be carried out by skilled radiologists and all ultrasound examinations will be performed on the same ultrasound machine to ensure consistency. mpMRI will be conducted at the Saint-André imaging department, on a different day than the CT, following standard clinical practice due to the use of contrast agents. The MRI examination lasts approximately 30–40 minutes and uses the routine gadolinium-based contrast agents DOTAREM® or CLARISCAN® at the recommended dose (0.1 mmol/kg). CT and MRI will ideally be scheduled within 15 days of each other, with a maximum allowed interval of 28 days to minimize the risk of tumor evolution between exams. Ultrasound elastography will be performed at Pellegrin Hospital, potentially during the same visit as the planning CT, and lasts approximately 15 minutes.

The T2 visit corresponds to the scheduled nephrectomy performed as part of routine care.

**Fig 1:**
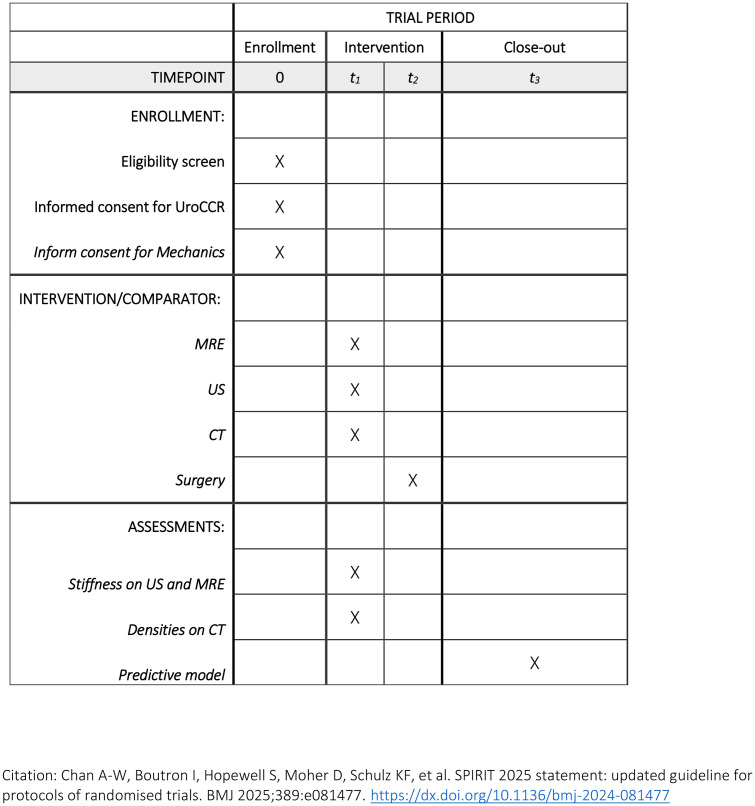
MECHANICS Protocole participant timeline.

**Fig 2:**
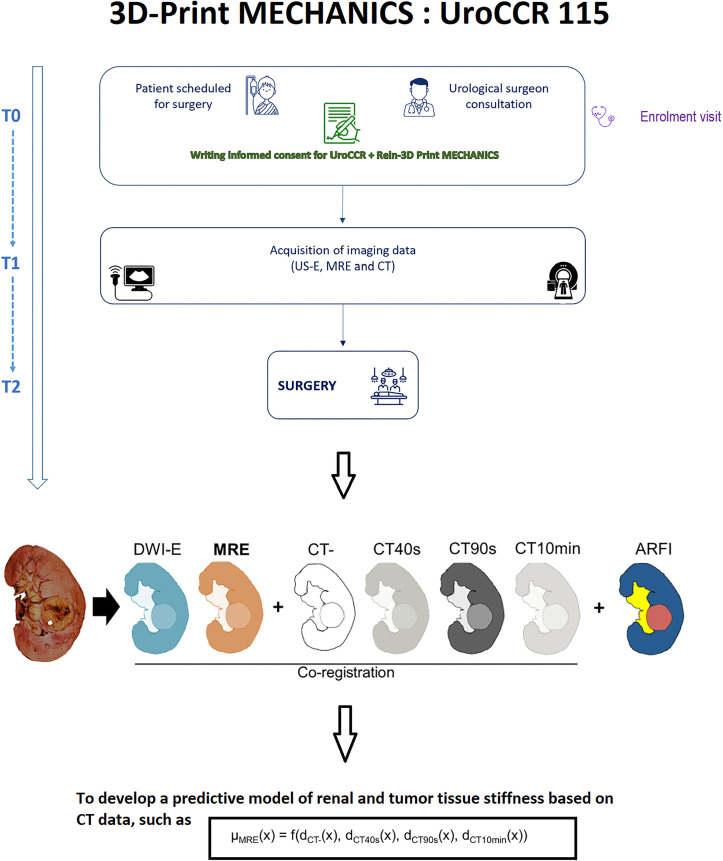
Study workflow.

No additional follow-up visits are planned. Study participation ends when the results of the routine histopathological examination become available, up to one month after surgery. The last research-related procedure corresponds to the final imaging examination (ultrasound or MRI), and at the latest the day before surgery. Subsequent clinical care is conducted according to standard practice (Figs 1 and 2).

### Outcome measures

The primary outcome of this study is to develop a predictive model of biomechanical properties of both normal and pathological renal tissue, using MRE as the reference, based on density values from multiphasic CT. All analyses will be conducted before the definitive histological diagnosis.

The secondary outcomes are:

- Multimodal correlation: to correlate elasticity values obtained by MRE, and ultrasound elastography with CT density values, on a voxel to voxel, ROI to ROI, and anatomical region basis across all CT acquisition phases.
- Feasibility of DWI-vMRE: This study aims to assess the feasibility and optimization of parameters for DWI-vMRE using a clinical 1.5-Tesla MRI scanner. It will focus on evaluating key performance metrics, including image quality (qualitative assessment), contrast-to-noise ratio (CNR), signal-to-noise ratio (SNR), and the identification and documentation of artifacts.
- Feasibility of elastography modalities: to assess the feasibility of MRE, and US–E for quantifying renal parenchymal and tumoral stiffness, and to identify limiting factors (e.g., BMI, sarcopenia, lesion location, size, and architecture), including enumeration of non-diagnostic examinations.
- Repeatability analysis: to evaluate the repeatability of MRE, and US-E across the cohort and within subgroups defined by patient morphotype and tumor characteristics, using intraclass correlation coefficients and Bland–Altman plots.
- De-correlation and bias analysis: to identify situations of de-correlation or potential biases between biomechanical measurements obtained from MRE and US-E (e.g., extreme elasticity values, morphotype-related limitations).
- Histopathological associations: to assess associations between biomechanical properties (measured or CT-predicted) and final histological subtype; and, for clear cell renal cell carcinoma, with histological grade. This includes ROC curve analyses and identification of potential diagnostic cutoff values.

### Imaging analysis workflow

CT and MRI datasets will be anonymized and transferred to a dedicated research workstation for postprocessing. Tumor and renal parenchyma segmentations will be performed semi-automatically by an imaging engineer and a radiologist with expertise in genitourinary imaging, blinded to histopathological results and elastography measurements. Segmentations will include the whole tumor volume on axial images. An in-board software (Sophia DDM for Radiomics, SophiaGenetics SA, Saint-Sulpice, Switzerland) was used.

For renal parenchyma analyses, cortical and medullary ROIs will be separately defined on anatomically matched slices.

Multimodal image co-registration between CT and MRI datasets will be performed using rigid registration followed, when necessary, by deformable registration to compensate for respiratory and positional differences. All images will be resampled to a common voxel resolution before voxel-wise analysis.

Quality control procedures will include visual assessment of registration accuracy and exclusion of non-diagnostic datasets.

### Statistical analysis

Imaging data will be acquired across multiple modalities, including MRE (μMRE), US-E, and CT (dCT, dCT40s, dCT90s, dCT10min), alongside clinical and morphological variables such as BMI, sarcopenia, lesion size, volume, location, histological type, malignancy status, and histological grade for clear cell renal carcinoma, where applicable. Quantitative variables will be extracted and analyzed for each region of interest (ROI). Descriptive statistics will summarize categorical variables as frequencies and percentages, while continuous variables will be presented as mean, standard deviation, median, range, and interquartile range.

The primary objective is to develop a regression model to predict μMRE based on CT-derived density values. The dataset will be divided into a 70% training set and a 30% test set, and various regression algorithms (linear regression, elastic net, k-nearest neighbors, support vector machines, random forests, and artificial neural networks) will be compared using repeated nested cross-validation. Model performance will be evaluated based on coefficient of determination (R²), mean squared error (MSE), and root mean squared error (RMSE), with RMSE serving as the primary criterion for model selection. Reproducibility of MRE measurements will be assessed through repeated acquisitions, with the highest-quality sequence selected for model development.

Secondary analyses will investigate the interrelationships between biomechanical parameters across imaging modalities. Spearman’s rank correlation coefficient will be used to assess correlations between elasticity values from MRE, US-E, and CT densities. The feasibility and image quality of DWI will be evaluated using a five-point qualitative scale, contrast-to-noise ratio, signal-to-noise ratio, and systematic reporting of artifacts. Non-diagnostic examinations and potential limiting factors—such as patient morphotype, sarcopenia, and lesion characteristics—will be documented. The repeatability of elastography measurements will be quantified using intraclass correlation coefficients and Bland-Altman plots. Cases of modality de-correlation will be explored through paired scatterplots and descriptive analysis of discordant data points.

The final statistical analysis plan will be reviewed and approved by the Scientific Committee prior to database lock.

## Expected results

This protocol introduces a multimodal evaluation model that integrates several imaging techniques, including CT, MRI and US-E. Despite their routine clinical use, their comparative value and the degree of complementarity among them have not been systematically examined in the same patients within a standardized preoperative interval. By generating voxel-level and ROI-level based datasets across modalities, the study will enable a level of correlation analysis that has not previously been possible.

**Fig 3:**
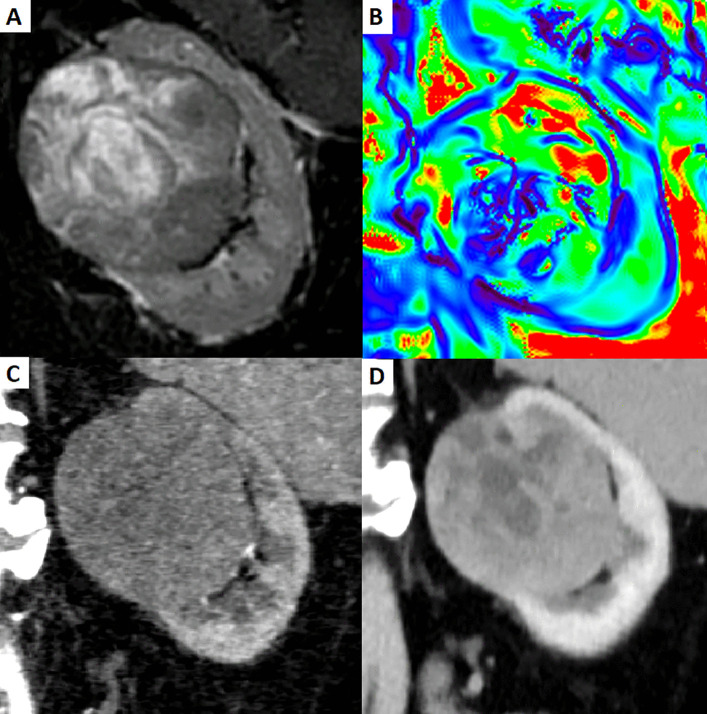
Example of corresponding slices in MRI and CT scans. Unclassified eosinophilic renal cell carcinoma (NOS), ISUP grade 3, measuring 7.9 cm in maximum diameter in the left kidney. The tumor is shown on **(A)** T2-weighted fat-suppressed MRI, **(B)** color-coded stiffness map obtained by MR elastography (shear wave velocity), **(C)** CT scan acquired during the cortical phase at 40 s, and **(D)** CT scan acquired during the nephrographic phase at 90 s. Multimodal imaging demonstrates tumor heterogeneity and supports the development of a biomimetic model based on these data.

A key strength of the study is its ability to associate all imaging measurements. This unique design reduces the ambiguity inherent in isolated imaging biomarkers and supports the development of a CT-based predictive model that could be broadly applicable, given the near-universal availability of multiphasic CT (Fig 3).

The study is exploratory by design and therefore has inherent limitations. The monocentric setting and the restricted sample size limit generalizability, although they are appropriate for a proof-of-concept investigation. The exclusion of certain patient profiles—particularly those with high BMI or complex anatomical constraints—is necessary to preserve imaging quality but narrows applicability in real-world practice. Furthermore, the absence of longitudinal imaging prevents assessment of temporal variability in biomechanical properties, which may be of interest in future research, especially in patients undergoing active surveillance or systemic therapy.

Despite these constraints, this protocol establishes a solid framework for a rigorous evaluation of biomechanical imaging in renal tumors. By clarifying the relationships between imaging-derived elasticity, CT density dynamics, and true tissue stiffness, the study may support the emergence of new diagnostic markers and improve the fidelity of 3D models used for planning and education. More broadly, the findings may contribute to the development of quantitative imaging tools that could ultimately refine diagnostic decision-making and reduce unnecessary surgical procedures.

### Monitoring

A Scientific Advisory Board met annually to oversee study conduct, ensuring protocol compliance, ethical standards, and study progress, and advising on modifications or continuation. No independent monitoring committee was established due to the low-risk nature of the study but could be implemented if needed. Substantial protocol amendments requiring changes affecting participant safety, study validity, or conduct were approved by the sponsor and relevant ethics and regulatory authorities (CPP, ANSM) prior to implementation, while non-substantial modifications were notified to the ethics committee. Investigators were informed of all changes, and updated consent was obtained when required.

## Supporting information

S1 File Mechanics protocol. (DOCX)

S2 File English mechanics protocol. (DOCX)

S3 File SPIRIT checklist. (DOCX)

S4 File Associated content. (DOCX)
