# Supplementary material for: Multimodal imaging to analyze the biomechanical properties of kidney tumors, evaluating feasibility, inter-modality correspondence, and diagnostic value (UroCCR-115)
Source: PLoS One. 2026 Jul 8;21(7):e0351477. doi: 10.1371/journal.pone.0351477 (PMC13345387; doi:10.1371/journal.pone.0351477)
Supplement: S1 File — (DOCX) [file pone.0351477.s001.docx]

Imagerie multimodale des propriétés biomécaniques des tumeurs rénales : étude de faisabilité, correspondance intermodalités des valeurs d’élasticité et valeur diagnostique (UroCCR-115).

**Rein 3D-Print Mechanics**

**Code promoteur :** CHUBX 2022/11

**PROTOCOLE DE RECHERCHE INTERVENTIONNELLE IMPLIQUANT**

**LA PERSONNE HUMAINE (Catégorie 1 - HPS)**

Version n°2.0 du 16/10/2025

**Numéro ID-RCB** : 2024-A00959-38

**Cette recherche a obtenu le financement de** **l’ANR dans le cadre du RHU Digital Urology 3D**

Promoteur :

**Centre Hospitalier Universitaire de Bordeaux**

12, rue Dubernat

33400 Talence

FRANCE

Investigateur principal :

**Dr Eva FOURAGE**

Imagerie diagnostique et interventionnelle de l’adulte

CHU Bordeaux - Hôpital Pellegrin

Place Amélie Raba Léon

33076 Bordeaux Cedex

Tel : 05 56 79 59 88 – Fax : 05 57 82 16 50

Mail : [eva.fourage@chu-bordeaux.fr](mailto:eva.fourage@chu-bordeaux.fr)

Responsable scientifique :

**Pr Jean-Christophe BERNHARD**

CHU de Bordeaux - Hôpital Pellegrin

Place Amélie Rabat Léon

33076 Bordeaux Cedex

Tel : 05 57 82 06 87 – Fax : 05 56 79 56 51

Mail : [jean-christophe.bernhard@chu-bordeaux.fr](file:///C:\Users\zazol\Downloads\jean-christophe.bernhard@chu-bordeaux.fr)

Centre de Méthodologie :

###### Pr Thierry COLIN

SOPHiA GENETICS

Technopôle Izarbel

374 Allée d’Abbadie

64210 Bidart

Mail: [TColin@sophiagenetics.com](file:///C:\Users\zazol\Downloads\TColin@sophiagenetics.com)

Unité de sécurité et de vigilance de la recherche clinique :

###### Dr Caroline LACOSTE

Direction de la Recherche Clinique et de l’Innovation

12, rue Dubernat

33404 Talence Cedex

Tel : 05 57 82 16 26 – Fax : 05 57 82 12 62

Mail : [vigilance.essais-cliniques@chu-bordeaux.fr](file:///C:\Users\zazol\Downloads\vigilance.essais-cliniques@chu-bordeaux.fr)

**Ce protocole a été conçu et rédigé à partir de la version 4.0 du 18/01/2022**

**du protocole-type du GIRCI SOHO**

Historique des mises à jour du protocole

| Version | Date | Raison de la mise à jour |
| --- | --- | --- |
| 1.0 | 16/04/2024 | Version initiale soumise au CPP et à l’ANSM |
| 1.1 | 07/06/2024 | Version modifiée suite aux remarques de l’ANSM |
| 1.2 | 26/06/2024 | Version modifiée suite aux remarques du CPP du 18/06/2024 |
| 1.3 | 22/07/2024 | Version modifiée suite aux remarques du CPP du 18/07/2024 |
| 2.0 | 16/10/2025 | Modification substantielle n°1 |

Page de signature du protocole

Imagerie multimodale des propriétés biomécaniques des tumeurs rénales : étude de faisabilité, correspondance intermodalités des valeurs d’élasticité et valeur diagnostique (UroCCR-115).

**Rein 3D-Print Mechanics**

**Code promoteur :** CHUBX 2022/11

**Promoteur**

Centre Hospitalier Universitaire de Bordeaux à Talence, le :

12, rue Dubernat

33400 Talence

Le Directeur Général du CHU de Bordeaux

V.N. DELPECH

Et par délégation, le Directeur de la

Recherche Clinique et de l’Innovation,

G. DULUC

à Bordeaux, le *:*

**Investigateur principal**

**Dr Eva FOURAGE**

Imagerie diagnostique et interventionnelle de l’adulte

CHU de Bordeaux – Hôpital Pellegrin

Place Amélie Raba Léon

33076 Bordeaux Cedex

Tel : 05 56 79 59 88 – Fax : 05 57 82 16 50

Mail : [eva.fourage@chu-bordeaux.fr](mailto:eva.fourage@chu-bordeaux.fr)

PRINCIPAUX CORRESPONDANTS

**Investigateur principal**

Dr Eva FOURAGE

Imagerie diagnostique et interventionnelle de l’adulte, CHU Bordeaux

Place Amélie Raba Léon

33076 Bordeaux Cedex

Tel : 05 56 79 59 88 – Fax : 05 57 82 16 50

Mail : [eva.fourage@chu-bordeaux.fr](mailto:eva.fourage@chu-bordeaux.fr)

**Co-investigateur**

Dr Yann LE BRAS

Mail : [yann.lebras@chu-bordeaux.fr](file:///C:\Users\zazol\Downloads\yann.lebras@chu-bordeaux.fr)

**Responsable scientifique**

Pr Jean-Christophe BERNHARD

CHU de Bordeaux - Hôpital Pellegrin

Place Amélie Rabat Léon

33076 Bordeaux Cedex

Tel : 05 57 82 06 87 – Fax : 05 56 79 56 51

Mail : [jean-christophe.bernhard@chu-bordeaux.fr](file:///C:\Users\zazol\Downloads\jean-christophe.bernhard@chu-bordeaux.fr)

**Co-responsables scientifiques**

Pr Amandine CROMBE

Mail : [amandine.crombe@chu-bordeaux.fr](mailto:amandine.crombe@chu-bordeaux.fr)

Dr Gaëlle MARGUE

Mail : [gaelle.margue@chu-bordeaux.fr](mailto:gaelle.margue@chu-bordeaux.fr)

**ARC support investigateur**

Mme Julie DUMAS

Service d’Urologie et Transplantation Rénale

CHU de Bordeaux – Pellegrin

Tel : 05 57 82 23 94 – Fax : 05 56 79 56 51

Mail : julie.dumas@chu-bordeaux.fr

**Data Manager UroCCR**

M. Guillaume HERMAN

Service d’Urologie et Transplantation Rénale

CHU de Bordeaux - Pellegrin

Tel : 05 56 79 60 50 – Fax : 05 56 79 56 51

Mail : [guillaume.herman@chu-bordeaux.fr](mailto:guillaume.herman@chu-bordeaux.fr)

**Unité de Soutien Méthodologique à la Recherche Clinique et épidémiologique**

Pr Laura RICHERT

Service d'information médicale, Pôle Santé publique CHU Bordeaux
146 rue Léo Saignat, case n°75
33076 Bordeaux Cedex
Tel : 05 57 57 11 29 / 14 42 - Fax : 05 57 57 15 78

Mail : [laura.richert@chu-bordeaux.fr](mailto:laura.richert@chu-bordeaux.fr)

**Unité de vigilance de la recherche clinique**

Dr Caroline LACOSTE (Médecin responsable)

Dr Marine ROUSSET (Pharmacien évaluateur)

Dr Magalie CASTOREO (Pharmacien évaluateur)

Sylvie GEORGEVAIL (Attachée de recherche clinique)

Valérie MARTY (Chargée de vigilance)

Direction de la recherche clinique et de l’innovation

12 rue Dubernat

33404 Talence Cedex

Tel: 05 57 82 16 26 - Fax: 05 57 82 12 62

[vigilance.essais-cliniques@chu-bordeaux.fr](mailto:vigilance.essais-cliniques@chu-bordeaux.fr)

**Promoteur**

Centre Hospitalier Universitaire de Bordeaux

12 rue Dubernat

33400 Talence

FRANCE

**Responsable de la recherche au niveau du promoteur**

M. Gilles DULUC – Directeur de la Recherche Clinique et de l’Innovation

Mail : [gilles.duluc@chu-bordeaux.fr](file:///C:\Users\zazol\Downloads\gilles.duluc@chu-bordeaux.fr)

Dr Anne GIMBERT – Responsable « Promotion interne »

Direction de la Recherche Clinique et de l’Innovation du CHU de Bordeaux

12 rue Dubernat

33404 Talence Cedex

Tel : 05 57 82 08 34 – Fax : 05 56 79 49 26

Mail : [anne.gimbert@chu-bordeaux.fr](mailto:anne.gimbert@chu-bordeaux.fr)

**Responsable d’Etudes Cliniques**

Mme Corinne CASTERMANS

Direction de la Recherche Clinique et de l’Innovation du CHU de Bordeaux

12 rue Dubernat

33404 Talence Cedex

Tel : 05 57 82 08 53– Fax : 05 56 79 49 26

Mail : [corinne.castermans@chu-bordeaux.fr](mailto:corinne.castermans@chu-bordeaux.fr)

**Centre de Statistiques**

###### Pr Thierry COLIN

SOPHiA GENETICS

Technopôle Izarbel

374 Allée d’Abbadie

64210 Bidart

Email: [TColin@sophiagenetics.com](mailto:TColin@sophiagenetics.com)

SOMMAIRE

1. RESUME de la recherche 9

Abstract 14

2. justification scientifique et description générale 17

2.1. Etat actuel des connaissances 17

2.1.1. Sur la pathologie 17

2.1.2. Sur les procédures de référence et à l’étude 17

2.2. Hypothèses de la recherche et résultats attendus 18

2.3. Rapport bénéfice / risque 18

2.4. Retombées attendues 19

3. Objectifs de la recherche 19

3.1. Objectif principal 19

3.2. Objectifs secondaires 19

4. CRITERES DE JUGEMENT 20

4.1. Critère de jugement principal 20

4.2. Critères de jugement secondaires 20

5. Conception de la recherche 21

5.2. Schéma de la recherche 21

6. critères d’Éligibilité 21

6.1. Critères d’inclusion 21

6.2. Critères de non inclusion 22

6.3. Faisabilité et Modalités de recrutement 22

7. PROCEDURE(S) de la recherche 22

7.1. procédure(S) expérimentale(S) 22

**7.1.1.** **L’élastographie par échographie via la méthode ARFI** 22

**7.1.2.** **L’élastographie par IRM via la méthode DWI-E et MRE** 23

7.2. procédure de comparaison 23

7.3. Post-traitement des images obtenues 24

8. DEROULEMENT DE la recherche 25

8.1. Calendrier de la recherche 25

8.2. Tableau récapitulatif du suivi participant 25

8.3. T0 : Visite d’/inclusion 26

8.3.1. Recueil du consentement 26

8.3.2. Déroulement de la visite 26

8.4. T1_ ACQUISITION D’IMAGERIE 27

8.5. T2 _ Chirurgie 27

8.6. Visite de SUIVIF 27

8.7. Visite de fin de la recherche 27

8.8. Règles d’arrêt 27

8.8.1. Arrêt de la participation d’une personne a la recherche 27

8.8.2. Arrêt de la recherche 28

8.9. Déviations au protocole 28

8.9.1. Arrêt prématuré et définitif de la procédure de la recherche 28

8.9.2. Participant perdu de vue 28

8.9.3. Participant inclus à tort 28

8.10. Participation simultanée à d’autres recherches, période d’exclusion, indemnisation 29

9. Gestion des ÉVÉNEMENTS INDÉSIRABLES, des grossesses et des faits nouveaux 29

9.1. Définitions 29

9.2. Description des événements indésirables attendus 29

9.3. Conduite à tenir par l’investigateur en cas d’evenement indesirable, de fait nouveau ou de grossesse 30

9.3.1. Recueil des évènements indésirables (EI) 30

9.3.2. Notification sans délai des évènements indésirables graves (EIG)et des faits nouveaux 30

9.3.3. Notification des grossesses 31

9.3.4. Tableau récapitulatif du circuit de notification par type d’évènement 31

9.4. Déclaration par le promoteur des effets indesirables graves inattendus, des faits nouveaux et autres evenements 31

9.5. Rapport annuel de sécurité 32

10. ASPECTS STATISTIQUES 33

10.1. Calcul de la taille d’étude 33

10.2. Méthodes statistiques employées 33

10.3. Plan d’analyse statistique 33

11. SURVEILLANCE DE La recherche 34

11.1. Conseil scientifique 34

11.1.1. Composition 34

11.1.2. Rythme des réunions 34

11.1.3. Rôles 34

11.2. Comité indépendant de Surveillance 34

12. GESTION ET TRAITEMENT DES données et documents source 35

12.1. Données et documents source 35

12.2. Consignes pour le recueil des données 35

12.3. Gestion et circuit des données 35

12.3.1. Logiciel de gestion de données 35

12.3.1.1. Logiciel utilisé 35

12.3.1.2. Hébergement des données 35

12.3.1.3. Sécurité des données 35

12.3.2. Saisie des données 35

12.3.3. Codage des données 36

12.3.4. Contrôles des données 36

12.3.5. Réconciliation des bases EI/EIG 36

12.3.6. Transfert des données 36

12.3.7. Archivage de la base de données 36

12.4. Confidentialité des données 36

12.5. Conservation des documents et des donNees relatifs à la recherche 37

12.6. Cession des données 37

13. contrôle et assurance qualité 37

13.1. Accès aux données 37

13.2. Contrôle Qualité 37

13.3. Audit et inspection 37

14. Considérations éthiques et réglementaires 38

14.1. Conformité aux textes de référence 38

14.2. Modifications au protocole 38

15. Rapport final 39

16. Regles relatives à la publication 39

16.1. Communications scientifiques 39

16.2. Communication des résultats aux participants 39

Références Bibliographiques 40

LISTE DES ABREVIATIONS

**ADC** Coefficient Apparent de Diffusion

**ANSM** Agence Nationale de Sécurité du Médicament

**ARFI** Acoustic Radiation Force Impulsion

**BMI** Body Mass Index

**CCCR** Carcinome Rénal à Cellules Claires

**CREDIM** Centre de Recherche et Développement en Informatique Médicale

**DFG** Débit de Filtration Glomérulaire

**DWI** Diffusion Weighted Imaging

**DWI-E** DWI Elastography

**e-CRF**  electronic Case Report Form

**EI** Evènement Indésirable

**EIG** Evènement Indésirable Grave

**ICC** Coefficient de Corrélation Intra-Classe

**ICH** International Council for Harmonisation

**IRM/MRI** Imagerie par Résonance Magnétique / Magnetic Resonance Imaging

**IRM-mp** IRM Multi-Paramétrique

**MR** Magnetic Resonance

**MRE** Magnetic Resonance Elastography

**MSE** Mean Squared Error

**OMS** Organisation Mondiale de la Santé

**R3DP-M** Etude Rein 3D Print-Mechanics

**RCP** Réunion de Concertation Pluridisciplinaire

**RMSE** Root Mean Square Error

**ROI** Region Of Interest / Région d’intérêt

**US** Ultrasound (Echographie)

RESUME de la recherche

| **Promoteur** | Centre Hospitalier Universitaire de Bordeaux  12, rue Dubernat  33400 Talence  FRANCE |
| --- | --- |
| **Investigateur principal** | Dr Eva FOURAGE  Imagerie diagnostique et interventionnelle de l’adulte  CHU Bordeaux – Hôpital Pellegrin  Place Amélie Raba Léon,  33076 Bordeaux Cedex  Tel : 05 56 79 59 88  Mail : [eva.fourage@chu-bordeaux.fr](mailto:eva.fourage@chu-bordeaux.fr) |
| **Responsable scientifique** | Pr Jean-Christophe BERNHARD  Service de Chirurgie Urologique et Transplantation  CHU de Bordeaux – Hôpital Pellegrin  Place Amélie Raba Léon  33076 Bordeaux Cedex  Tel : 05 57 82 03 50  Mail : [jean-christophe.bernhard@chu-bordeaux.fr](file:///C:\Users\zazol\Downloads\jean-christophe.bernhard@chu-bordeaux.fr) |
| **Titre** | **Rein 3D-Print MECHANICS**  Imagerie multimodale des propriétés biomécaniques des tumeurs rénales : étude de faisabilité, correspondance intermodalités des valeurs d’élasticité et valeur diagnostique |
| **Justification / contexte** | L’imagerie médicale joue un rôle central dans la prise en charge diagnostique et thérapeutique des carcinomes rénaux. En effet, elle permet de confirmer la présence d’une tumeur, de la localiser, d’orienter vers la malignité voire vers le sous-type histologique, de guider les prélèvements, de réaliser le staging TNM, d’aider à la planification opératoire, de suivre l’efficacité thérapeutique en cas de traitement systémique, de guider des gestes d’ablathermie et de rechercher des rechutes après la fin des traitements curatifs.  L’imagerie des tumeurs rénales repose sur trois modalités d’imagerie complémentaires : l’échographie (US) basée sur les ultrasons, le scanner basé sur l’absorption des rayons X et l’imagerie par résonance magnétique (IRM) basée sur la résonance magnétique nucléaire.  L’examen le plus couramment réalisé reste le scanner et c’est cette modalité d’imagerie qui est utilisée pour préparer les impressions 3D. Or, l’étude des corrélations entre d’une part les densités du parenchyme rénal et les tumeurs rénales (avant et au cours des différents temps d’acquisition du scanner après injection), et d’autre part les paramètres d’élasticité mesurés en échographie et IRM, n’a jamais été exploré. Etre capable de prédire avec précision la dureté des tissus sur la base du scanner permettrait d’améliorer les performances prédictives du scanner et la qualité des impressions 3D de rein tumoral.  Ce projet innovant a pour objectif d’évaluer la correspondance entre plusieurs modalités d’imagerie pour la caractérisation de l’élasticité du tissu rénal sain et pathologique. Il permettra également d’améliorer le réalisme des impressions 3D utilisées par les chirurgiens urologues et d’identifier de nouveaux biomarqueurs d’imagerie complémentaires. |
| **Objectifs** | **Objectif principal :**  Développer un modèle prédictif des propriétés biomécaniques du tissu rénal normal et pathologique évaluées par la méthode de référence (MR-élastographie) à partir des densités obtenues sur les différentes phases du scanner. Les modèles prédictifs seront évalués selon plusieurs indices (R2, MSE, RMSE). Nous sélectionnerons les meilleurs modèles selon la RMSE.  Cette évaluation est réalisée avant le diagnostic histologique définitif.  **Objectifs secondaires** :   1. Corréler voxel à voxel, ROI à ROI, région anatomique à région anatomique, l’élasticité des tumeurs rénales et du tissu rénal sain environnant obtenu par MR-élastographie et par US-élastographie, avec les valeurs de densité des différentes phases de l’uro-scanner 2. Etudier la faisabilité et le paramétrage de la DWI-élastographie sur une IRM 1.5-Tesla clinique :  - évaluation de la qualité (selon une échelle qualitative ordinale en 5 points), - du contraste (calcul du ratio contraste sur bruit), - du bruit (calcul du ratio signal sur bruit), - des artefacts (annotation des artefacts rencontrés);  1. Etudier la faisabilité de la MR-élastographie, DWI-élastographie, US-élastographie pour mesurer l’élasticité du parenchyme rénal et de tumeurs rénales ; identification des facteurs limitants (morphotype patient [body mass index, BMI], sarcopénie, localisation, taille et architecture de la lésion) (dénombrement de situations où l’examen n’a pas de valeur clinique diagnostique et associations aux potentiels facteurs limitant) 2. Evaluer la répétabilité de la MR-élastographie, DWI-élastographie, et US-élastographie sur le parenchyme rénal et de tumeurs rénales : sur l’ensemble de l’échantillon, selon le morphotype du patient, la localisation, la taille et l’architecture de la lésion (calcul des coefficients de corrélation intra-classe et traces des plots de Bland-Altmann) 3. Identifier des situations de dé-corrélation (ou non-corrélation) et biais potentiels entre propriétés biomécaniques obtenues par MR-élastographie, DWI-élastographie et US-élastographie (valeurs d’élasticité extrêmes, morphotype patient...) 4. Réaliser des associations entre propriétés biomécaniques obtenues par prédictions du modèle scanographique, MR-élastographie, DWI-élastographie, US-élastographie et le type histologique final de la lésion (si CCCR : associations avec le grade histologique) (associations entre variables catégorielles histologiques et numériques d’élasticité – comparaison d’aire sous la courbe ROC, identification de cut-off le cas échéant) |
| **Critères de jugement** | **Critère de jugement principal :**  Le critère de jugement principal est la racine de l’erreur quadratique moyenne (ou « root mean square error », RMSE – sans unité). L’objectif est de réussir à prédire **μ_MRE_** à partir de **d_CT-_, d_CT40s_, d_CT90s_, d_CT10min_** (en nommant μ_CT_ la prédiction du modèle scanographique) avec l’erreur la plus faible possible.  **Critères de jugement secondaires :**   1. Le critère de jugement sera le rho de Spearman, avec pour objectif qu’il soit le plus élevé possible (maximum = 1). 2. L’évaluation de la qualité sera basée sur une échelle qualitative ordinale en 5 points. Le contraste sera calculé grâce au ratio contraste sur bruit. Le bruit sera évalué par le calcul du ratio signal sur bruit. La présence d’artefacts sera annotée pour chaque examen. 3. Identification des facteurs limitants (morphotype patient [body mass index, BMI], sarcopénie, localisation, taille et architecture de la lésion) (dénombrement de situations où l’examen n’a pas de valeur clinique diagnostique et associations aux potentiels facteurs limitant) 4. Les critères de jugement seront les coefficients de corrélation intra-classe et les traces des plots de Bland-Altmann. L’objectif est que le score intra-classe soit le plus élevé possible (maximum = 1, idéalement >0.90) 5. Les situations de décorrélation entre les mesures de dureté obtenues par les différentes modalités d’imagerie seront identifiées visuellement en traçant pour chaque patient et situation anatomique avec appariement possible, les nuages de point avec en X la dureté par une modalité d’imagerie et en Y la dureté obtenue par une autre modalité d’imagerie. Les caractéristiques descriptives des points (voxel ou segmentation d’un patient) avec décorrelation seront ensuite analysés. 6. Les critères de jugement reposeront sur les associations entre variables catégorielles histologiques et numériques d’élasticité avec des comparaisons d’aires sous la courbe ROC ou l’identification de cut-off le cas échéant. Le caractère malin / bénin (variable binaire) et le type histologique (variable catégorielle non ordinale) seront évalués dans le cadre du soin courant par l’anatomopathologiste sénior expert en urologique du CHU |
| **Schéma de la recherche** | Etude exploratoire d’évaluation diagnostique, monocentrique prospective , basée sur des examens d’imagerie |
| **Critères d’inclusion** | - Age ≥ 18 ans, - Prise en charge chirurgicale programmée pour néphrectomie pour tumeur du rein dans le département d’urologie du CHU de Bordeaux, - Uro-scanner disponible ou programmé pour la chirurgie, - Consentement exprimé pour intégration de la cohorte UroCCR, - Consentement exprimé pour participation à l’étude Rein-3D Print Mechanics, - Personne affiliée ou bénéficiaire d’un régime de sécurité sociale |
| **Critères de non inclusion** | - - Femme enceinte ou allaitante, - Contre-indication à l’IRM   - Présence d’un dispositif médical électronique contre-indiquant l’IRM (pacemaker, défibrillateur, implants cochléaires, neurostimulateur), stent datant de moins de 6 mois, valve cardiaque incompatible, présence de corps étranger métallique intra-oculaires, grossesse en cours.   - Contre-indication à l’injection de produits de contraste gadolinés,   - Réalisation d’une éventuelle biopsie dans les 15 jours avant la réalisation des IRM, scanners et échographie protocolaires (donc à risque d’altération artéfactuelle, via des remaniements hémorragiques iatrogènes, des propriétés biomécaniques de la tumeur rénale et du parenchyme rénal)   - Présence de matériel d’arthrodèse thoraco lombaire   - Patient obèse (body mass index ≥ 30 kg/m2)   - Tumeurs rénales kystiques avec composante solide (correspondant soit à un épaississement pariétal ou bourgeon tumoral) < 2 cm   - Tumeurs rénales nécrotiques avec composante solide (correspondant soit à un épaississement pariétal ou bourgeon tumoral) < 2 cm   - Ascite   - Personne faisant l’objet d’une mesure de protection légale (sauvegarde de justice, tutelle ou curatelle)   - Difficultés à comprendre et à s’exprimer en français |
| **Stratégie/ procédures de la recherche** | Chez tous les patients : IRM rénale avec séquences de DWI et MR-élastographie selon les protocoles d’acquisition et d’interprétation standardisés, couplé à une échographie avec US élastographie 🡪 dans les 28 jours suivant le scanner de planification pré-opératoire et avant la chirurgie ou toute autre procédure invasive diagnostique (biopsie, ablathermie, embolisation...).  Analyse anatomopathologique de la pièce excisée couplée à une mesure de la dureté physique du tissu à la réception de l’état frais. |
| **Taille d’étude** | 50 patients |
| **Durée de la recherche** | Durée de la période d’inclusion : 18 mois  Durée de participation de chaque participant : entre 15 jours et 4 mois  Durée totale de la recherche : 22 mois |
| **Analyse statistique des données** | A l’issue des acquisitions d’imagerie et d’une étape de co-registration, nous obtiendrons pour chaque région d’intérêt (ROI, tracée à la main – i.e., analyse par ROI), pour chaque région anatomique du rein (cortex, médullaire, tumeur – i.e., analyse par région), pour chaque voxel (i.e., analyse par voxel), les variables suivantes :  MR-élastographie : μMRE  DWI: ADCb200, ADCb1500, μDWI  US-élastographie : μUS (uniquement dans l’analyse par ROI)  Scanner : dCT-, dCT40sec, dCT90sec, dCT10min  Nous aurons aussi un ensemble de variables possiblement confondantes lié au patient (âge, sexe, BMI, sarcopénie, débit de filtration glomérulaire [DFG], hauteur de chaque rein, épaisseur du cortex de chaque rein, distance peau-mesure) et lié à la lésion (taille, volume, localisation antérieure/postérieure, polaire supérieure/équatoriale/inférieure, endophytique/exophytique)  Ainsi que le diagnostic final des lésions : bénin / malin, le type histologique, et pour les CCR le grade histologique.  Toutes ces variables seront décrites en termes de nombre absolu et de pourcentages pour les variables catégorielles et en moyenne, écart-type, médiane, minimum, maximum et écart inter-quartile pour les variables numériques   - Concernant **l’objectif principal** consistant à développer un modèle prédictif de y = μMRE selon les variables X = dCT-, dCT40sec, dCT90sec, dCT10min (dit modèle scanographique) : nous disposerons de plusieurs observations par patients, de différentes natures (tumeur, parenchyme sain), obtenues selon différents moyens (voxels, ROI, régions anatomiques).   L’échantillon sera scindé en un échantillon d’entraînement et un échantillon de test (70%-30% de la population). Plusieurs algorithmes de régression seront entraînés en nested cross-validation répétées (régression linéaire, régression linéaire pénalisée elasticnet, k-nearest neighbors, support vector machine, random forest, artificial neural network...) et sélectionnés selon la RMSE. Les performances des meilleurs modèles seront ensuite évaluées de manière indépendante sur l’échantillon de test.   - Concernant les **objectifs secondaires** : - Les corrélations pour chaque paire de variables d’imagerie sur l’ensemble des ROIs seront évaluées par le test des rangs de Spearman. - Les relations entre μDWI, μMRE et μUS et les densités scanographiques aux différents temps seront explorées de manière empirique (linéaire, logarithmique, quadratique...) - La répétabilité des mesures d’élasticité sera évaluée par la méthode de Bland-Altmann et par le coefficient de corrélation intra-classe (ICC) - Les associations entre les prédictions du modèle scanographique, μDWI, μMRE et μUS, les variables patients et lésionnelles potentiellement confondantes seront étudiées, ainsi que les associations entre les prédictions du modèle scanographique, μDWI, μMRE et μUS et les caractéristiques cliniquement pertinentes des lésions rénales à l’aide de tests de Spearman (pour les couples de variables numériques), de tests de Mann-Whitney (pour les couples de variables catégorielle et numérique) et de tests de Chi-2 et de Fisher (pour les couples de variables catégorielles) |
| **Retombées attendues** | - Modèle prédictif de l’élasticité rénale par IRM selon les données scanners - Validation d’une séquence de diffusion-élastographie à 1.5-Tesla - Meilleure connaissance des facteurs physiologiques, pathologiques et artéfactuels influençant l’élasticité du tissu rénal sain et tumoral - Identification de biomarqueurs originaux du caractère malin de la lésion rénale, du type histologique, voire du grade histologique. - Brevet potentiel pour le modèle prédictif scanographique   - Les résultats de cette étude pourraient apporter de nouveaux arguments diagnostiques afin d’améliorer la caractérisation des lésions rénales et le réalisme de modèles imprimés en 3D à visées d’éducation thérapeutique et de formation |

Abstract

This research has been registered in <http://www.clinicaltrials.gov/> under the n° NCT06525831

**Multimodal imaging of the biomechanical properties of kidney tumors: feasibility, inter-modality correspondence and diagnostic value (UroCCR-115). R3DP-M**

The University Hospital of Bordeaux is the sponsor of this research.

This research will be conducted with the support of ANR-21-RHUS-0015.

- **Brief summary:**

The goal of this innovative project is to evaluate the correspondence between several imaging modalities for characterizing the elasticity of healthy and pathological renal tissue which could help improve the realism of 3D prints used by urological surgeons and allow the identification of new, complementary imaging biomarkers. The main objective is to develop a predictive model of the biomechanical properties of normal and pathological kidney tissue, as assessed by the reference method (MR-elastography).

- **Detailed description :**

Medical imaging plays a key role in the diagnostic and therapeutic management of renal cell carcinomas. It can be used to confirm the presence of a tumor, localize it, suggest malignancy or even histological subtype, guide sampling, perform TNM staging, assist in surgical scheduling, monitor therapeutic efficacy in the event of systemic treatment, guide ablathermy procedures and look for relapses after curative treatments have ended. Imaging of kidney tumors relies on three complementary imaging modalities: ultrasound (US), CT based on X-ray absorption (CT-scan) and magnetic resonance imaging (MRI). The most commonly performed examination remains the CT scan, which is used to print 3D models. However, the correlation between renal parenchyma densities and renal tumors (before and during the scan acquisition times after injection) and elasticity parameters measured by US and MRI has never been explored.

This trial aim to evaluate the correspondence between several imaging modalities for characterizing the elasticity of healthy and pathological kidney tissue. It will also improve the realism of 3D models used by surgeons, and identify new complementary imaging biomarkers.

To achieve this aim, 50 patients will undergo DWI-elastography and MR-elastography sequences, as well as an US before the surgery. **Primary outcome:**

Measurement of the root mean square error (RMSE). The aim is to successfully predict μMRE from dCT-, dCT40s, dCT90s, dCT10min (naming μCT the scan model prediction) with the lowest possible error.

- **Secondary outcomes:**

1. Measure of Spearman's rho, aiming for the highest possible value (maximum = 1).
2. Measure of the qualitative assessment based on a 5-point ordinal qualitative scale. Calculation of contrast using the contrast-to-noise ratio. Evaluation of noise using the signal-to-noise ratio. The presence of artifacts will be annotated for each examination.
3. Identification of limiting factors (patient morphotype [body mass index, BMI], sarcopenia, lesion location, size and architecture) (count situations where the examination has no clinical diagnostic value, and associate with potential limiting factors).
4. Evaluation of the repeatability of MR-elastography, DWI-elastography and US-elastography on renal parenchyma and renal tumors: on the whole sample, according to patient morphotype, lesion location, size and architecture (calculation of intra-class correlation coefficients and Bland-Altmann plot traces).
5. Identification of situations of de-correlation (or non-correlation) and potential bias between biomechanical properties obtained by MR-elastography, DWI-elastography and US-elastography (extreme elasticity values, patient morphotype, ...).
6. Carry out associations between biomechanical properties obtained by CT model predictions, MR-elastography, DWI-elastography, US-elastography and final histological type of lesion (if CCCR: associations with histological grade) (associations between histological categorical variables and numerical elasticity variables - comparison of area under the ROC curve, identification of cut-offs where appropriate).

- **Study design :** Exploratory, monocentric, prospective, imaging-based diagnostic evaluation

study

- **Eligibility criteria:**
- Inclusion criteria:
  - Adult patients (≥ 18 years of age)
  - Scheduled for surgical management with nephrectomy for kidney tumor in the urology department of Bordeaux University Hospital
  - CT scan available or scheduled for surgery
  - Consent expressed for integration of the UroCCR database
  - Expressed consent for participation in the Rein 3D Print Mechanics study
  - Patients affiliated or benefiting from social security system
- Exclusion criteria:
  - Pregnant or breast-feeding women
  - Contraindication to MRI
  - Contraindication to injection of gadoline contrast agents
  - Biopsy performed within 15 days prior to MRI, CT and ultrasound scans (risk of artifactual alteration, via iatrogenic hemorrhagic changes, of the biomechanical properties of the renal tumor and parenchyma).
  - Presence of thoracolumbar arthrodesis material
  - Obese patient (body mass index ≥ 30 kg/m²)
  - Cystic renal tumors with solid component (corresponding to either parietal thickening or tumor bud) < 2 cm
  - Necrotic renal tumors with solid component (corresponding to either parietal thickening or tumor bud) < 2 cm
  - Ascites
  - Person under legal protection
  - Difficulty understanding and expressing in French

*.*

- **Interventions** : We will add a MR-elastography and Ultrasound exams for the research. The exams will

be performed between the inclusion of the patient and the day before the surgery.

- **Number of subjects:** 50
- **Statistical analysis:**

After imaging acquisitions and a co-registration step, we will obtain for each region of interest, each anatomical region of the kidney and each voxel, the following variables:

- MR-elastography: μMRE
- DWI-elastography: ADCb200, ADCb1500, μDWI
- US-elastography: μUS (only in ROI analysis)
- CT-Scan: dCT-, dCT40sec, dCT90sec, dCT10min

We will also have a set of possibly confounding variables related to the patient, the lesion and the final diagnosis of the lesions: benign/malignant, histological type, and for RCC the histological grade.

The main objective consists in developing a predictive model of y = μMRE according to the variables X = dCT-, dCT40sec, dCT90sec, dCT10min (so-called scanographic model): we will have several observations per patient, of different natures (tumor, healthy parenchyma), obtained by different means (voxels, ROI, anatomical regions).

The sample will be split into a training sample and a test sample (70%-30% of the population). Several regression algorithms will be trained in repeated nested cross-validation (linear regression, elasticnet penalized linear regression, k-nearest neighbors, support vector machine, random forest, artificial neural network...) and selected according to RMSE. The performance of the best models will then be independently evaluated on the test sample.

- **Condition:** Kidney cancer surgery
- **Key-words:** Renal-Cell Carcinoma, 3D Printing, Renal Elasticity, Biomarkers, Ultrasound, CT-scan, Magnetic Resonance Imaging, AFRI, Diffusion weighted imaging

justification scientifique et description générale

- 1. Etat actuel des connaissances
     1. Sur la pathologie

Les carcinomes rénaux représentent 3 à 5% de l’ensemble des tumeurs malignes de l’adulte, la 7^ème^ cause de mortalité par cancer chez l’homme et la 10^ème^ chez la femme (1). Plus de 50% des carcinomes rénaux sont découverts de manière fortuite, au cours d’un bilan d’imagerie pour une autre indication (2). Selon la classification de l’Organisation Mondiale de la Santé (OMS), les principaux types histologiques suivants sont distingués : (i) les carcinomes à cellules claires rénales, qui représentent 80% des carcinomes rénaux, (ii) les carcinomes papillaires de types 1 et 2 (le dernier ayant une présentation phénotypique plus agressive) et (iii) les carcinomes chromophobes.

L’imagerie médicale joue un rôle central dans la prise en charge diagnostique et thérapeutique des carcinomes rénaux. En effet, elle permet de confirmer la présence d’une tumeur, de la localiser, d’orienter vers la malignité voire vers le sous-type histologique, de guider les prélèvements, de réaliser le staging TNM, d’aider à la planification opératoire, de suivre l’efficacité thérapeutique en cas de traitement systémique, de guider des gestes d’ablathermie et de rechercher des rechutes après la fin des traitements curatifs.

Si ces différents cancers présentent certains traits radiologiques permettant d’évoquer leur diagnostic, en particulier sur l’IRM multi-paramétrique (IRM-mp), ces traits radiophénotypiques restent insuffisamment sensibles et spécifiques pour permettre un diagnostic précis systématique. En effet, selon une récente méta-analyse regroupant 1239 lésions rénales solides, le pourcentage de lésions bien classées en terme de malignité par l’IRM est de 64%, la sensibilité de 95% et la spécificité de 63% (3). Dans une autre étude multicentrique et prospective rassemblant 250 tumeurs de moins de 4 cm, la sensibilité et la spécificité de l’IRM était de 75% et 79%, respectivement, pour le diagnostic de carcinome à cellules claires (CCCR), avec une reproductibilité inter-observateur restant modeste (kappa = 0.58) (4).

Ainsi, 30% des néphrectomies partielles et totales sont réalisées pour des tumeurs bénignes (notamment, angiomyolipomes pauvres en graisse et oncocytomes) – qui sont autant de gestes invasifs qui auraient pu être évités, avec des complications locales non-négligeables (hémorragies, fistules, infections – dans 16% des cas) (2,5).

Il apparaît donc essentiel d’améliorer la caractérisation des tumeurs rénales par l’imagerie, notamment au travers d’informations complémentaires et indépendantes. L’évaluation des propriétés biomécaniques des tumeurs rénales, par échographie ou IRM, et même scanner, pourrait ainsi enrichir l’analyse radiologique et améliorer le réalisme des modèles 3D du rein sain et pathologique.

- - 1. Sur les procédures de référence et à l’étude

Trois modalités d’imagerie complémentaires sont employées pour l’analyse du parenchyme rénal sain, pathologique et les tumeurs rénales : l’échographie, le scanner et l’IRM.

L’examen apportant le plus d’information pour caractériser une tumeur rénale est l’IRM à l’aide de protocoles dits multiparamétriques (IRM-mp) comprenant des séquences morphologiques simples (T1, T2), des séquences permettant d’identifier la graisse microscopique (T1 en inversion-opposition de phase), la vascularisation tumorale (acquisitions dynamiques après injection intraveineuse de produit de contraste), d’analyser la cellularité et l’architecture tumorale (diffusion). Néanmoins, comme expliqué précédemment, l’IRM-mp, même en s’aidant d’algorithme de machine-learning pour mieux intégrer les données extraites dans des modèles prédictifs, ne permet pas d’assurer systématiquement un diagnostic correct (accuracy = 64% pour le diagnostic de malignité d’après une méta-analyse sur 1239 patients (3) – 81-98% selon les sous-types histologiques en s’appuyant sur l’intelligence artificielle dans des travaux exploratoires non validés cliniquement (6–8)).

De plus, l’IRM, via des séquences de diffusion spécifiquement paramétrées (DWI-E) et des séquences d’élastographie IRM (MRE), peut quantifier l’élasticité des tissus avec des résultats préliminaires encourageants sur le rein sain et pathologique (tumoral, maladie rénale chronique et fibrose). Ainsi, des travaux ont montré que la dureté du parenchyme rénal était inversement corrélée au pourcentage de fibrose tubulo-interstitielle, au volume de matrice extracellulaire et à la proportion de glomerulosclérose chez des patients atteints de lésions rénales chroniques (9,10). D’autres études ont montré une association entre des valeurs d’élasticité intra-tumorale et certains types histologiques (11). Ces travaux suggèrent l’intérêt diagnostic de cette quantification des propriétés biomécaniques du rein sain et pathologique.

Cependant, l’examen de référence utilisé pour segmenter les différents éléments de l’anatomie rénale (veine, artère, cortex et médullaire du rein, voies excrétrices) et ainsi permettre la réalisation de modèles 3D à visée planificatrice, éducationnelle et informative, est le scanner, à l’aide d’acquisition multiphasique avant et après injection mais pour lequel, jusqu’à présent, aucune relation n’a été identifiée avec l’élasticité des tissus. Etre en mesure de prédire les valeurs d’élasticité d’un tissu (en prenant pour référence les valeurs de MRE), grâce au scanner multiphasique, pourrait permettre d’améliorer le réalisme des modèles 3D fabriqués qui s’appuieraient donc sur les densités des voxels en plus des données anatomiques issues du scanner, voire d’améliorer l’information prédictive (de malignité, d’histotype) apportée par le scanner.

- 1. Hypothèses de la recherche et résultats attendus

Dans ce projet, nous faisons les hypothèses suivantes :

- qu’il existe une corrélation entre les élasticités rénales mesurées par IRM et par échographie, mais qu’elle pourrait être imparfaite et dépendre de facteurs indépendants (par exemple, des facteurs morphométriques du patient)
- qu’il existe une relation entre densités obtenues sur scanner multiphasique et valeurs d’élasticité mesurées par MRE.
  1. Rapport bénéfice / risque

**Bénéfices pour les patients :**

Les bénéfices pour le patient individuel sont limités en dehors de lui donner la certitude qu’il bénéficiera d’un bilan d’imagerie pré-opératoire exhaustif et optimal. Les constatations biomécaniques réalisées au cours des examens d’imagerie ne sont pas susceptibles de modifier la prise en charge du patient réalisée en soin courant. La séquence d’élasto-IRM réalisée dans le cadre du protocole peut néanmoins permettre d’apporter des informations complémentaires en termes de caractérisation tumorale et d’aide à la chirurgie future.

En revanche, il existe un potentiel bénéfice collectif futur. Ce projet permettra de mieux comprendre les liens entre les propriétés biomécaniques du rein et des tumeurs rénales selon les différentes modalités d’imagerie. A terme, ce projet pourra permettre (i) d’apporter des éléments de caractérisation supplémentaire face à une tumeur rénale (permettant éventuellement d’éviter des biopsies et néphrectomies inutiles) et (ii) de réaliser de meilleurs modèles 3D pour le chirurgien urologue afin d’améliorer la qualité de l’information transmise au patient ainsi que la préparation et l’entraînement au geste opératoire.

**Risques attendus :**

La prise en charge (consultations urologiques, biopsie voire chirurgie d’exérèse) se fait intégralement dans le cadre du soin courant à l’exception de l’échographie pour élastographie et de l’élasto-IRM, En effet, même si actuellement l’IRM tient une place prépondérante dans le bilan pré-thérapeutique des lésions rénales et est fréquemment réalisée en complément du scanner pour mieux les caractériser, cet examen ne fait pas partie de la prise en charge recommandée à ce jour.

Ces examens ne sont ni douloureux, ni irradiants, ni invasifs. La réalisation d’une élasto-IRM avec ou sans injection de produit de contraste est un examen sans risque si l’on respecte les contre-indications.

L’élasto-IRM est un examen bruyant mais un casque de protection auditive est systématiquement proposé. L’examen nécessite également un dispositif de vibration lombaire pendant toute la durée de la séquence, jugée comme largement supportable par les patients d’études antérieures (12). En cas de gêne trop importante pour le patient, le choix des séquences à réaliser sera adapté afin de réduire au maximum le temps de l’examen. En priorité seront réalisées la séquence T2 pour le repérage, la séquence d’élasto-IRM sur le rein tumoral et la séquence DWI.

L’échographie est un examen ne présentant aucun risque. Ces examens ne sont uniquement contraignants de par le temps qu’ils nécessitent (environ 20 minutes pour l’échographie, 20 à 40 minutes pour l’IRM).

Le rapport bénéfice/risque est en faveur de la conduite de l’étude. En effet, les bénéfices potentiels indirects sont nombreux en termes de connaissance scientifique, d’implémentation de techniques innovantes et prometteuses au CHU de Bordeaux et de familiarisation des équipes médicales et paramédicales avec de nouvelles techniques d’imagerie. Il n’y a pas de risque pour le patient lié aux pratiques cliniques ou à un éventuel retard de prise en charge puisque l’échographie et l’IRM surnuméraire seront réalisées sans délai.

L’investigateur principal doit constamment surveiller, évaluer et documenter les risques et doit s’assurer qu’ils pourront être gérés de manière satisfaisante.

- 1. Retombées attendues

Les retombées attendues sont :

- d’un point de vue scientifique : l’amélioration des connaissances sur l’imagerie des propriétés biomécaniques du rein sain et pathologique (les précédentes études dédiées à la technique MRE n’ayant inclus que moins de 50 patients au total, aucune n’ayant formellement étudié spécifiquement la technique DWI-E, aucune n’ayant mis en parallèle l’ensemble des méthodes d’imagerie pour le rein, aucune n’ayant étudié les relations avec les densités scanographiques). Ainsi, nous prévoyons au moins une publication scientifique en imagerie, et une en urologie sur des applications de ces quantifications sur les modèles 3D, et les communications orales correspondantes (Journées Françaises de Radiologie, Congrès Européen de Radiologie).
- d’un point de vue éducationnel : ce projet est l’occasion d’implémenter en routine clinique des techniques d’imagerie innovantes du rein et de familiariser les équipes médicales et paramédicales à leur utilisation.
- d’un point de vue relationnel : ce projet est l’occasion de faire interagir des équipes médicales et scientifiques et des partenaires industriels autour d’une thématique commune, susceptible de renforcer une communauté médico-scientifique autour de thématiques communes
- d’un point de vue clinique : en cas de résultats positifs, cette étude apportera de nouveaux arguments diagnostiques pour améliorer la caractérisation des lésions rénales et le réalisme de modèles imprimés en 3D à visées d’éducation thérapeutique et de formation.

Objectifs de la recherche

- 1. Objectif principal

L’objectif principal est de développer un modèle prédictif des propriétés biomécaniques du tissu rénal normal et pathologique évaluées par la méthode de référence (MRE) à partir des densités obtenues sur les différentes phases du scanner. Les modèles prédictifs seront évalués selon plusieurs indices (R2, MSE, RMSE). Nous sélectionnerons les meilleurs modèles selon la RMSE.

Cette évaluation est réalisée avant le diagnostic histologique définitif.

- 1. Objectifs secondaires

Les objectifs secondaires sont :

1. Corréler voxel à voxel, ROI à ROI, région anatomique à région anatomique, l’élasticité des tumeurs rénales et du tissu rénal sain environnant obtenu par MR-élastographie et par US-élastographie, avec les valeurs de densité des différentes phases de l’uro-scanner
2. Etudier la faisabilité et le paramétrage de la DWI-élastographie sur une IRM 1.5-Tesla clinique :
   - évaluation de la qualité (selon une échelle qualitative ordinale en 5 points),
   - du contraste (calcul du ratio contraste sur bruit),
   - du bruit (calcul du ratio signal sur bruit),
   - des artefacts (annotation des artefacts rencontrés);
3. Etudier la faisabilité de la MR-élastographie, DWI-élastographie, US-élastographie pour mesurer l’élasticité du parenchyme rénal et de tumeurs rénales ; identification des facteurs limitants (morphotype patient [body mass index, BMI], sarcopénie, localisation, taille et architecture de la lésion) (dénombrement de situations où l’examen n’a pas de valeur clinique diagnostique et associations aux potentiels facteurs limitant)
4. Evaluer la répétabilité de la MR-élastographie, DWI-élastographie, et US-élastographie sur le parenchyme rénal et de tumeurs rénales : sur l’ensemble de l’échantillon, selon le morphotype du patient, la localisation, la taille et l’architecture de la lésion (calcul des coefficients de corrélation intra-classe et traces des plots de Bland-Altmann)
5. Identifier des situations de dé-corrélation (ou non-corrélation) et biais potentiels entre propriétés biomécaniques obtenues par MR-élastographie, DWI-élastographie et US-élastographie (valeurs d’élasticité extrêmes, morphotype patient...)
6. Réaliser des associations entre propriétés biomécaniques obtenues par prédictions du modèle scanographique, MR-élastographie, DWI-élastographie, US-élastographie et le type histologique final de la lésion (si CCCR : associations avec le grade histologique) (associations entre variables catégorielles histologiques et numériques d’élasticité – comparaison d’aire sous la courbe ROC, identification de cut-off le cas échéant)

CRITERES DE JUGEMENT

- 1. Critère de jugement principal

L’objectif principal étant de développer un modèle dit scanographique permettant de prédire la dureté des tissus biologiques rénaux sains et pathologiques selon la technique MRE sur la base des densités de l’uro-scanner, nous nous positionnons donc dans un problème de régression.

Le critère de jugement principal est la racine de l’erreur quadratique moyenne (ou « root mean square error », RMSE – sans unité). L’objectif est de réussir à prédire **μ_MRE_** à partir de **d_CT-_, d_CT40s_, d_CT90s_, d_CT10min_** (en nommant μ_CT_ la prédiction du modèle scanographique) avec l’erreur la plus faible possible. En supposant que nous ayons **‘n’** observations (voxels, ROIs, régions), nous souhaitons donc développer le modèle minimisant :


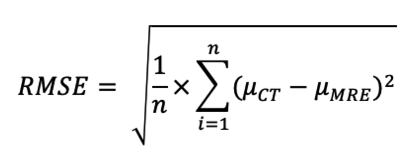


**μ_MRE_** sera mesurée lors de l’IRM protocolaire via la séquence MRE à 1.5 T en kPa (Philips Achieva dStream de l’hôpital Saint André).

Les personnes impliquées dans le recueil des données seront les radiologues séniors du département d’imagerie uro-vasculaire du CHU de Bordeaux.

Cette mesure est réalisée deux fois au cours d’une même session, pour évaluer la reproductibilité de la séquence. La séquence qualitativement la plus réussie par les radiologues est utilisée dans le développement de l’algorithme de régression.

Il n’y a pas de traitement différentiel, donc pas de besoin d’aveugle.

- 1. Critères de jugement secondaires

Les critères de jugements secondaires sont définis pour répondre aux différents objectifs secondaires :

1. Le critère de jugement sera le rho de Spearman, avec pour objectif qu’il soit le plus élevé possible (maximum = 1).
2. L’évaluation de la qualité sera basée sur une échelle qualitative ordinale en 5 points. Le contraste sera calculé grâce au ratio contraste sur bruit. Le bruit sera évalué par le calcul du ratio signal sur bruit. La présence d’artefacts sera annotée pour chaque examen.
3. Identification des facteurs limitants (morphotype patient [body mass index, BMI], sarcopénie, localisation, taille et architecture de la lésion) (dénombrement de situations où l’examen n’a pas de valeur clinique diagnostique et associations aux potentiels facteurs limitant)
4. Les critères de jugement seront les coefficients de corrélation intra-classe et les traces des plots de Bland-Altmann. L’objectif est que le score intra-classe soit le plus élevé possible (maximum = 1, idéalement >0.90)
5. Les situations de décorrélation entre les mesures de dureté obtenues par les différentes modalités d’imagerie seront identifiées visuellement en traçant pour chaque patient et situation anatomique avec appariement possible, les nuages de points avec en X la dureté par une modalité d’imagerie et en Y la

dureté obtenue par une autre modalité d’imagerie. Les caractéristiques descriptives des points (voxel ou segmentation d’un patient) avec décorrelation seront ensuite analysés.

1. Les critères de jugement reposeront sur les associations entre variables catégorielles histologiques et numériques d’élasticité avec des comparaisons d’aires sous la courbe ROC ou l’identification de cut-off le cas échéant. Le caractère malin / bénin (variable binaire) et le type histologique (variable catégorielle non ordinale) seront évalués dans le cadre du soin courant par l’anatomopathologiste sénior expert en urologie du CHU

Conception de la recherche

- 1. **Justification des choix methodologiques**

Il s’agit d’une étude prospective exploratoire d’imagerie multimodale des propriétés biomécaniques des tumeurs rénales. Ainsi, les patients atteints de tumeur rénale remplissant les critères d’éligibilité participeront à l’étude et des modalités d’imagerie pré-opératoires spécifiques à la recherche (élastographie IRM et ultrasons) seront utilisées en plus des modalités de routine (CT Scan et IRM multiparamétrique).

Le schéma suit un principe d’étude transversale, avec une fenêtre pré-opératoire autorisée pour les différentes imageries.

La taille de l’échantillon (50 patients) est compatible avec le caractère exploratoire de l’étude. Les unités d’analyse pour l’imagerie seront le voxel (IRM et scanner) et la ROI (US).

- 1. Schéma de la recherche

Etude exploratoire prospective monocentrique


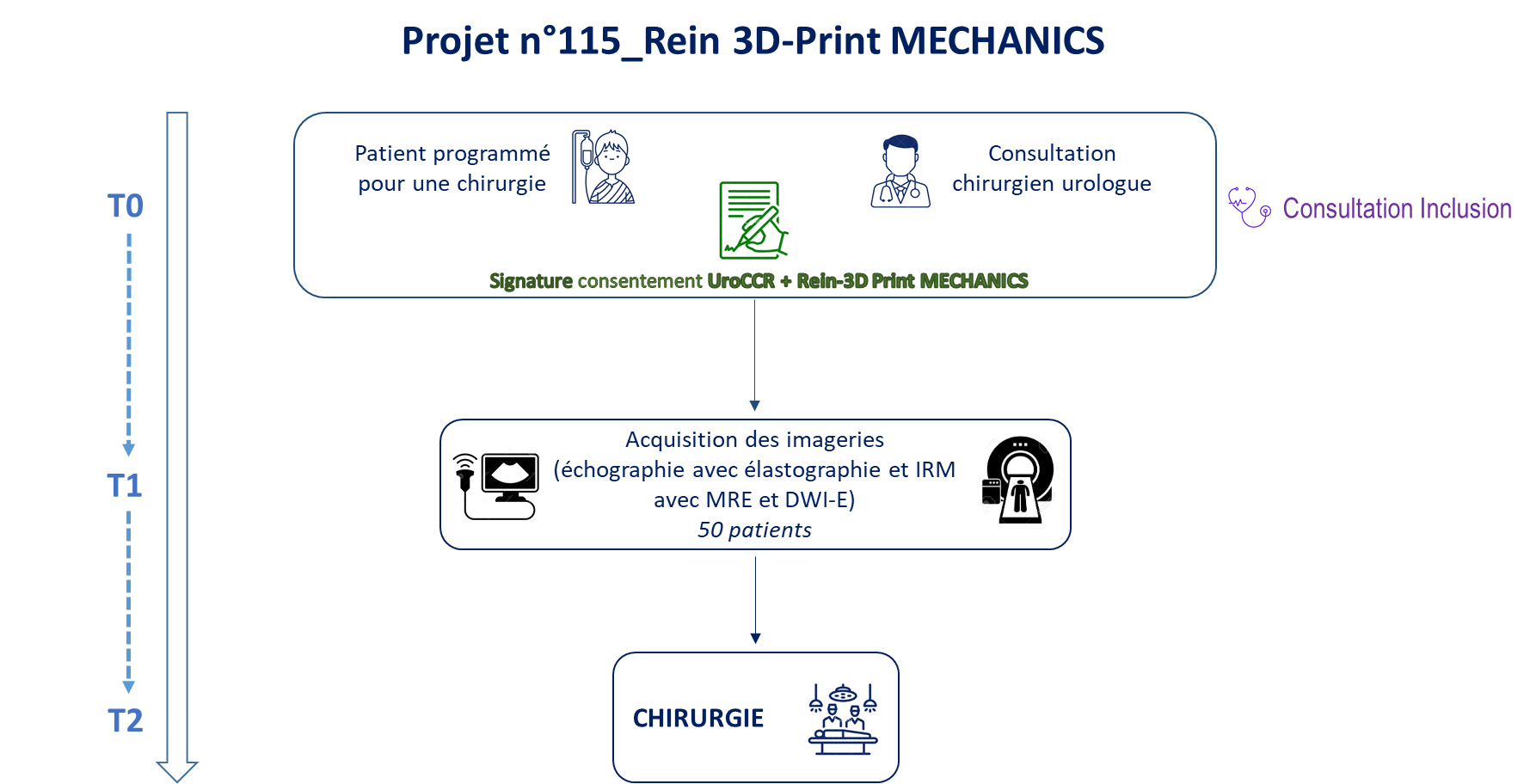


critères d’Éligibilité

- 1. Critères d’inclusion
  - Age ≥ 18 ans,
  - Prise en charge chirurgicale programmée pour néphrectomie pour tumeur du rein dans le département d’urologie du CHU de Bordeaux,
  - Uro-scanner disponible ou programmé pour la chirurgie,
  - Consentement exprimé pour intégration de la cohorte UroCCR,
  - Consentement exprimé pour participation à l’étude Rein-3D Print Mechanics,
  - Personne affiliée ou bénéficiaire d’un régime de sécurité sociale
  1. Critères de non inclusion
  - Femme enceinte ou allaitante,
- Contre-indication à l’IRM
  - Présence d’un dispositif médical électronique contre-indiquant l’IRM (pacemaker, défibrillateur, implants cochléaires, neurostimulateur), stent datant de moins de 6 mois, valve cardiaque incompatible, présence de corps étranger métallique intra-oculaires, grossesse en cours.
  - Contre-indication à l’injection de produits de contraste gadoliné,
  - Réalisation d’une éventuelle biopsie dans les 15 jours avant la réalisation des IRM, scanners et échographie protocolaires (donc à risque d’altération artéfactuelle, via des remaniements hémorragiques iatrogènes, des propriétés biomécaniques de la tumeur rénale et du parenchyme rénal)
  - Présence de matériel d’arthrodèse thoraco lombaire
  - Patient obèse (body mass index ≥ 30 kg/m2)
  - Tumeurs rénales kystiques avec composante solide (correspondant soit à un épaississement pariétal ou bourgeon tumoral) < 2 cm
  - Tumeurs rénales nécrotiques avec composante solide (correspondant soit à un épaississement pariétal ou bourgeon tumoral) < 2 cm
  - Ascite
  - Personne faisant l’objet d’une mesure de protection légale (sauvegarde de justice, tutelle ou curatelle)
  - Difficultés à comprendre et à s’exprimer en français
  1. Faisabilité et Modalités de recrutement

Le nombre de patients adressé aux départements d’urologie et de radiologie du CHU de Bordeaux pour primo-diagnostic de lésions rénales est d’environ 250 / an.

Ces deux départements sont habitués à travailler en partenariat aussi bien en clinique qu’en recherche et participent aux mêmes Réunions de Concertation Pluridisciplinaires RCP où sont discutées les prises en charge diagnostiques et thérapeutiques des tumeurs rénales.

Les patients seront recrutés par les urologues du département d’urologie de l’hôpital Pellegrin, lors d’une consultation avec un patient extérieur au CHU de Pellegrin adressé pour une tumeur rénale répondant aux critères d’inclusion ou après adressage par les radiologues du département de radiologie adulte de l’hôpital Pellegrin (lors de leurs vacations d’imagerie, s’ils rencontrent un patient adressé pour caractérisation d’une tumeur rénale répondant aux critères d’inclusion).

Ainsi, inclure 50 patients sur une durée de 18 mois, complété par une période de suivi de 4 mois après inclusion apparaît faisable.

PROCEDURE(S) de la recherche

- 1. procédure(S) expérimentale(S)
     1. **L’élastographie par échographie via la méthode ARFI**

L’élastographie par échographie via la méthode ARFI (Acoustic Radiation Force Impulsion, disponible sur les appareils cliniques Siemens avec module ARFI du CHU) utilise des pulses courts (0.03-0.4 ms) pour exciter les tissus et créer une onde de cisaillement qui sera analysée dans un volume d’échantillonnage pour en inférer une mesure focale de la vitesse de cisaillement et secondairement de l’élasticité

Cette région d’excitation correspond en échographie à une ROI rectangulaire de 1×0,5cm que l’on peut déplacer librement dans les tissus jusqu’à une profondeur maximale de 8cm par rapport au plan cutané. La mesure est proportionnelle à l’élasticité tissulaire et s’exprime en mètre par seconde, correspondant à la vitesse de l’onde de cisaillement perpendiculaire à l’impulsion source.

En pratique, lors de l’échographie les patients sont installés en décubitus latéral. La ROI est positionnée au sein de la tumeur sous contrôle visuel du mode bidimensionnel. Dix mesures sont effectuées puis la médiane est utilisée comme référence.

La durée supplémentaire de l’examen est de moins de 5 minutes. Il est parfaitement indolore, non-invasif et ne comporte aucune contre-indication.

- - 1. **L’élastographie par IRM via la méthode DWI-E et MRE**

Les IRM seront réalisées au CHU de Bordeaux sur l’IRM 1.5 T Philips Achieva dStream de l’hôpital Saint André. Cette IRM est équipée du module Resoundant® (Resoundant Inc., Mayo Clinic)

Concernant la méthode DWI-E, nous appliquerons la technique proposée par Le Bihan et al. dans Radiology en 2017, basée sur une formule empirique reliant l’élasticité des tissus aux valeurs du coefficient apparent de diffusion ADCb=200 et ADCb=1500, obtenues pour des acquisitions via des séquences de diffusion avec une pondération b = 200 s.mm-2 et b = 1500 s.mm-2, respectivement (13). La séquence DWI ne nécessite pas d’injection de produit de contraste gadoliné.

La séquence MRE permet d’étudier les propriétés mécaniques des tissus en 3 étapes : d'abord, une membrane externe envoie une onde mécanique au corps humain; ensuite, une séquence d'IRM capture les images de la propagation de cette onde dans les tissus; finalement, un algorithme analyse la propagation de cette onde et en calcule des cartes d'élasticité des tissus. La mesure proportionnelle à l’élasticité tissulaire s’exprime en kPa.

En pratique, lors de l'IRM abdominale standard, les patients sont placés en décubitus dorsal avec une membrane passive de 19 cm de diamètre placée sous le dos, contre la table d’examen. L'antenne abdominale est alors placée au-dessus d’eux. Des vibrations acoustiques continues sont ensuite transmises du dispositif localisé en salle technique à la membrane passive via un tuyau d'air, puis au rein.

Une séquence écho de gradient 2D est utilisée pour collecter des images des ondes de cisaillement dans le parenchyme rénal. 4 coupes sont acquises, à l'aide de 4 apnées successives, préalablement définies et centrées sur la tumeur rénale étudiée.

Le temps nécessaire pour installer la membrane et acquérir les images est d'environ 5-10 minutes (en plus de l’examen standard d’une durée d’environ 30 minutes).

Les contre-indications sont celles de l'IRM. Il n'y a aucune restriction supplémentaire liée à ces séquences particulières qui sont parfaitement non invasives et indolores pour le patient.

- 1. procédure de comparaison
- La programmation d’un uro-scanner de planification en vue de la chirurgie est une condition d’inclusion. Les données de cet uro-scanner serviront d’inputs (données d’entrée) au modèle scanographique*.*
- L’uro*-*scanner de planification est réalisé dans le cadre du soin courant, au CHU de Bordeaux, dans le service d’imagerie de l’Hôpital Pellegrin. Il comporte 5 acquisitions dont 4 serviront de comparaison pour l’analyse de texture (temps sans injection, temps artériel à 40 secondes, temps néphrographique à 90 secondes et temps tardif à 10 minutes après injection de 20 mg de Furosémide (soin courant). Une hélice est uniquement à visée pré-opératoire. Il s’agit de l’acquisition au temps artériel précoce, déclenchée automatiquement par détection de l’arrivée de produit de contraste iodé au niveau de l’aorte.
- L’IRM-mp est maintenant régulièrement réalisée en complément du scanner afin de mieux caractériser les lésions rénales. Cet examen représente environ 450 IRM par an au CHU de Bordeaux, soit un tiers de l’activité d’IRM pour indication urologique. L’IRM-mp est réalisée selon un protocole standardisé correspondant aux publications, sujet de recherche pour lequel le service d’Imagerie uro-vasculaire du CHU de Bordeaux est actif (14–16).
- Les tumeurs rénales des patients inclus seront opérées dans le cadre du soin courant et selon l’approche la plus adaptée pour RCP urologique selon l’ensemble des données à disposition. Cette procédure ne fait pas partie des pratiques évaluées par le projet (elle serait dans tous les cas réalisée dans le cadre du soin), mais elle permettra d’obtenir le diagnostic anatomopathologique final qui est un des paramètres évalués en objectif secondaire.
  1. Post-traitement des images obtenues

La **Figure 1** illustre les imageries réalisables dans la population d’étude :

*
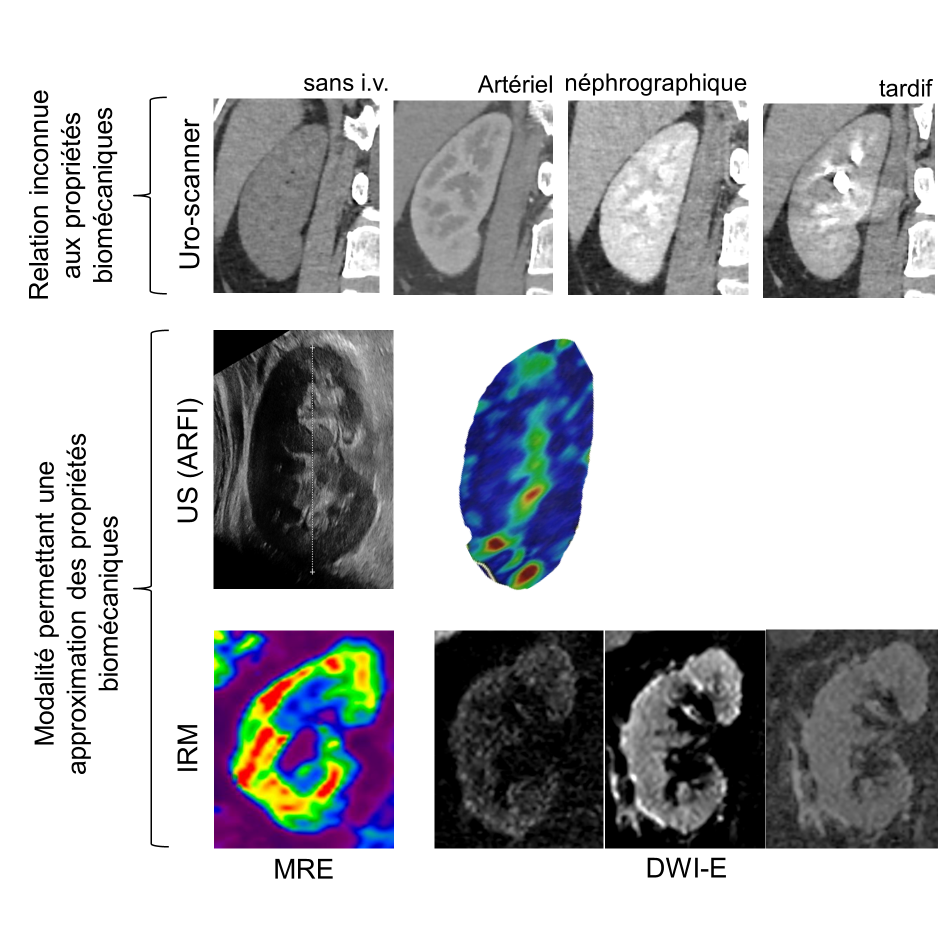
*

**Figure 1.** Différentes modalités d’imagerie réalisables sur le rein (exemple d’un rein sain)

Les cartes d’élasticité des tissus obtenues par DWI-E et MRE et les phases de l’uroscanner seront toutes co-registrées afin d’obtenir un bon appariement voxel-à-voxel.

Les localisations des mesures d’élasticité par échographie seront standardisées.

Les observations x en un même point de l’espace des densités scanographiques et des mesures d’élasticité par DWI-E et MRE pourront correspondre :

- soit à des voxels de même taille (après standardisation des tailles de voxel et interpolation des valeurs des voxels)
- soit à des régions anatomiques (segmentées automatiquement par deep learning, ou manuellement)
- soit à des régions d’intérêt dessinées manuellement selon un protocole standardisé (cas de l’échographie)

Nous explorerons ces trois méthodes afin d’obtenir les valeurs de référence d’élasticité par MRE (variable à prédire) et les valeurs d’entrée du modèle (densités aux différentes phases, i.e., prédicteurs), qui serviront à entraîner différents algorithmes de machine learning dédiés à la régression afin d’obtenir in fine le modèle scanographique le plus performant.

La **figure 2** illustre le pipeline global de l’étude.

*
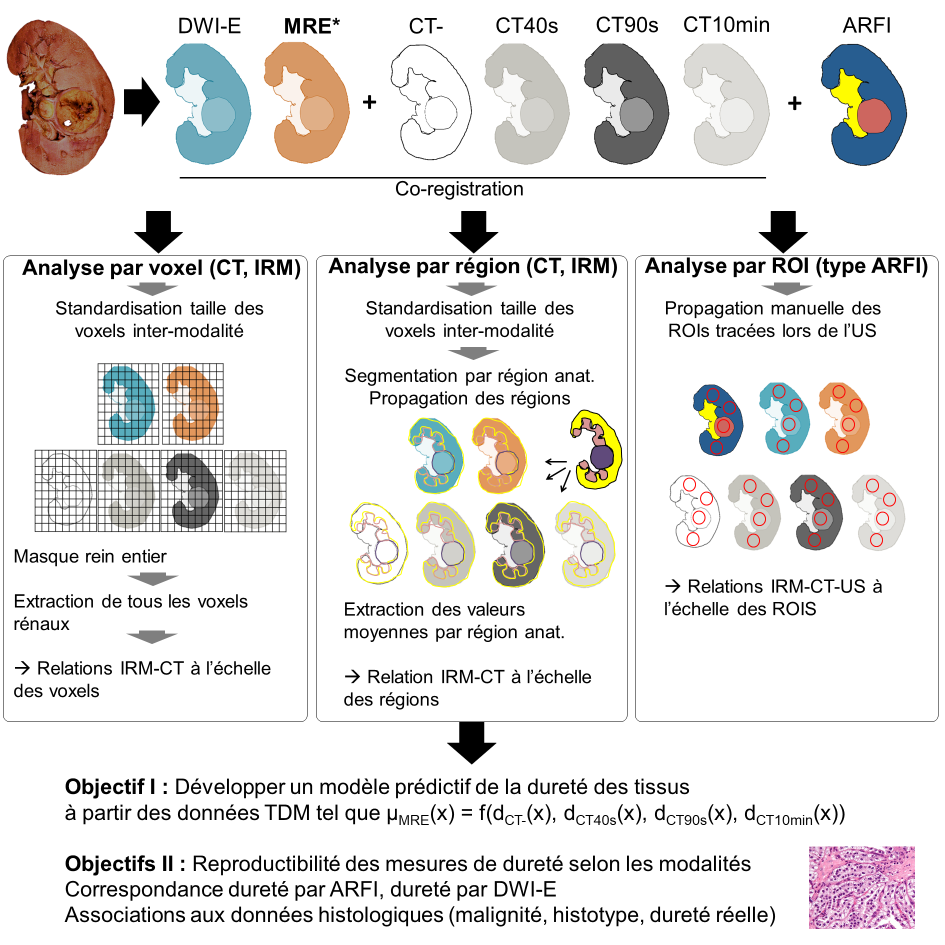
*

**Figure 2**. Schéma global de l’analyse et des moyens d’obtention des données pour le développement du modèle prédictif scanographique de la dureté des tissus rénaux sains et pathologiques.

DEROULEMENT DE la recherche

- 1. Calendrier de la recherche
- Durée de la période d’inclusion : 18 mois
- Durée de participation maximale de chaque participant : entre 15 jours et 4 mois
- Durée totale de la recherche: 22 mois
  1. Tableau récapitulatif du suivi participant

|  | Inclusion | Acquisition Imageries | Chirurgie (S) |
| --- | --- | --- | --- |
|  | T0 | T1  Entre T0 et la veille de la chirurgie au plus tard | T2  Entre T0 et T0 + 4 mois |
| Vérification éligibilité (R) | ✓ |  |  |
| Information et recueil du consentement éclairé (R) | ✓ ^3^ |  |  |
| Uro-scanner injecté disponible ou programmé avant chirurgie (S) ^1^ | ✓ ^2^ |  |  |
| Echographie avec élastographie (ARFI) (R) |  | ✓ ^2^ |  |
| IRM avec MRE et DWI-E (R) |  | ✓ ^2^ |  |
| Recueil des EI / EIG (R) |  | ✓ | ✓ |
|  |  |  |  |

^1^Si l’Uro-scanner du soin n’est pas disponible à la visite T0, il sera programmé entre la visite d’inclusion et la veille de la chirurgie.

^2^ Les examens Uro-scanner, échographie et IRM devront être programmés afin de respecter idéalement un intervalle de 2 jours entre le scanner et l’IRM (et maximum 28 jours)

*^3^* Le recueil du consentement pourra se faire entre la consultation d’inclusion et le premier examen d’imagerie de la recherche (IRM ou échographie)

*R :examen réalisé spécifiquement dans le cadre de la recherche
S :examen réalisé en soin courant.*

- 1. T0 : Visite d’/inclusion
     1. Recueil du consentement

Lors de la visite d’inclusion, le médecin investigateur informe le participant et répond à toutes ses questions concernant l'objectif, la nature des contraintes, les risques prévisibles et les bénéfices attendus de la recherche. Il précise également les droits du participant dans le cadre d’une recherche et vérifie les critères d’éligibilité.

Un exemplaire de chaque note d’information (REIN 3D-PRINT MECHANICS UroCCR n°115 et UroCCR) est alors remis au participant par le médecin investigateur. Après cette séance d’information, le participant dispose d’un délai de réflexion.

Si le participant donne son accord de participation, ce dernier et l’investigateur inscrivent leurs noms et prénoms en clair, datent et signent les formulaires de consentement en deux exemplaires originaux. Ceux-ci doivent être signés AVANT LA REALISATION DE TOUT EXAMEN CLINIQUE OU PARACLINIQUE NECESSITE PAR LA RECHERCHE.

Les différents exemplaires des notes d’information et des formulaires de consentement sont alors répartis comme suit :

- Un exemplaire original de chaque note d'information et de chaque consentement signé est remis au participant.
- Les autres exemplaires originaux seront conservés par l’investigateur (même en cas de déménagement du participant pendant la durée de la recherche) dans un lieu sûr inaccessible à des tiers.
  - 1. Déroulement de la visite

La visite d’inclusion est assurée par l’investigateur qui vérifie l’ensemble des critères d’éligibilité. Avant tout examen lié à la recherche, l’investigateur recueille le consentement libre et éclairé du participant (ou de son représentant légal le cas échéant).

Un test de grossesse urinaire sera réalisé pour les femmes en âge de procréer. Si le test est positif, la patiente sera exclue.

Dans le cadre du soin courant, le patient aura un uro-scanner injecté qui sera réalisé avant la chirurgie si non disponible lors de la visite d’inclusion.

Après avoir donné son accord de participation, le patient recevra des informations sur les modalités d’organisation et de déroulement des examens d’échographie et d’IRM réalisés dans le cadre de la recherche.

- 1. T1_ ACQUISITION D’IMAGERIE

Les examens seront réalisés entre l’inclusion et la chirurgie par les radiologues séniors du département d’imagerie uro-vasculaire, toujours sur le même échographe.

- Réalisation de l’élasto-IRM

L’IRM sera réalisée un jour différent du scanner, comme ce qui est fait en pratique courante, du fait de l’injection de produit de contraste au scanner et si besoin à l’IRM. Elle sera réalisée dans le département d’imagerie de Saint-André et durera 20-minutes.

Les produits de contraste utilisés dans le cadre de la recherche sont ceux habituellement utilisés au CHU de Bordeaux, le DOTAREM^®^ et le CLARISCAN^®^. Le dosage utilisé correspond à la recommandation de l’AMM, soit un dosage de 0,1 mmol/kg pour l’injection de solutions de DOTAREM ou CLARISCAN 0,5 mmol/mL.

L’uro-scanner et l’IRM devront être programmés afin de respecter idéalement un intervalle de 2 jours minimum entre le scanner et l’IRM. Le délai maximum ne devra pas être de plus de 28 jours.

- Réalisation de l’échographie

L’échographie sera réalisée sur le site de l’hôpital Pellegrin et pourra être combiné à l’uro-scanner de planification. L’examen durera 15 minutes.

- 1. T2 _ Chirurgie

La visite T2 correspond à l’intervention chirurgicale programmée dans le cadre de la prise en charge du soin courant. Aucune intervention en lien avec la recherche n’est effectuée lors de cette visite.

- 1. Visite de SUIVI

Aucune visite de suivi n’est prévue dans le cadre de la recherche.

- 1. Visite de fin de la recherche

La fin de participation à l’étude se fera lors du recueil des résultats de l’examen anatomopathologique réalisé sur la pièce opératoire dans le cadre du soin (date du compte-rendu anatomopathologique, jusqu'à 1 mois après la chirurgie).

Il n’y a pas de visite de fin de recherche proprement dite, le patient est suivi pendant le délai requis pour réaliser les examens d’imagerie. La visite de fin de recherche correspond à la fin du dernier examen (échographie ou IRM) et au plus tard la veille de la chirurgie. Le patient est ensuite suivi dans le cadre du soin courant pour la prise en charge de sa pathologie.

- 1. Règles d’arrêt
     1. Arrêt de la participation d’une personne a la recherche

Le participant qui souhaite abandonner ou retirer son consentement de participation à la recherche (comme il est en droit de le faire à tout moment) n’est plus suivi dans le cadre du protocole, mais doit faire l’objet de la meilleure prise en charge possible compte tenu de son état de santé et de l’état des connaissances du moment.

Un **abandon** est une décision d’un participant inclus de faire valoir son droit d’interrompre sa participation à une recherche, à tout moment au cours du suivi, sans qu'il n'encoure aucun préjudice de ce fait et sans avoir à se justifier.

Un **retrait de consentement** est une décision d’un participant de revenir sur sa décision de participer à une recherche et de faire valoir son droit d’annuler son consentement éclairé, à tout moment au cours du suivi et sans qu'il n'encoure aucun préjudice de ce fait et sans avoir à se justifier.

L’investigateur doit identifier la cause de l’abandon/du retrait de consentement et évalue s’il est possible de recueillir la variable sur laquelle porte le critère de jugement principal au moment de l’abandon/du retrait de consentement. Les abandons/retraits de consentement doivent être notifiés rapidement au centre investigateur coordonnateur, au promoteur et au centre de méthodologie et de gestion des données. Leurs raisons et leur date doivent être documentées dans le cahier d’observation et dans le dossier médical du participant.

- - 1. Arrêt de la recherche

**Fin de la recherche ou arrêt prévu de la recherche** : terme de la participation de la dernière personne qui se prête à la recherche aussi appelé dernière visite du dernier participant inclus dans la recherche.

Lorsque la recherche a atteint son terme prévu (arrêt prévu), la fin de la recherche doit être déclarée à l’ANSM et au CPP dans un délai de 90 jours.

**Arrêt anticipé de la recherche :** la recherche clinique est arrêtée (définitivement) de façon anticipée. C’est le cas, notamment, lorsque le promoteur décide :

- de ne pas commencer la recherche malgré l'obtention de l'autorisation de l'ANSM et de l'avis favorable d’un CPP ;
- de ne pas reprendre la recherche après l’avoir interrompu temporairement ou après sa suspension par l’ANSM.

Lorsque la recherche est arrêtée (définitivement) de façon anticipée, la fin de la recherche doit être déclarée au CPP et à l’ANSM dans un délai de 15 jours en indiquant les raisons qui motivent cet arrêt.

**Arrêt temporaire de la recherche :** l'arrêt temporaire d'une recherche clinique consiste en :

- l'arrêt de l'inclusion de nouvelles personnes dans cette recherche;
- et/ou l’arrêt de la pratique des actes prévus par le protocole de la recherche.

Toute décision du promoteur d'interrompre temporairement la recherche doit faire l'objet d'une information immédiate à l'ANSM et au CPP concerné et dans un second temps et dans un délai maximum de 15 jours calendaires suivant la date de cette interruption, d'une demande d’autorisation de modification substantielle concernant cet arrêt temporaire soumise à l'ANSM et d’une demande d’avis au CPP concerné.

- 1. Déviations au protocole

Les déviations peuvent concerner tous les aspects d’un protocole de recherche : processus d’inclusion, suivi, mesure des critères de jugement, traitements. Toutes doivent être documentées par l’investigateur et discutées en Conseil Scientifique.

Seuls les abandons entraînent un arrêt du suivi. Même en cas de déviation au protocole, le suivi du participant doit être mené jusqu’au terme prévu dans le protocole.

- - 1. Arrêt prématuré et définitif de la procédure de la recherche

Les participants en arrêt prématuré continuent à être suivis comme prévu par le protocole. En aucun cas, le suivi prévu ne doit être modifié.

Le participant doit faire l’objet de la meilleure prise en charge possible compte tenu de son état de santé et de l’état des connaissances du moment.

- - 1. Participant perdu de vue

Un participant est considéré comme perdu de vue quand il arrête le suivi prévu dans le cadre du protocole sans raison connue de l’investigateur, de sorte que le recueil des données ne peut pas être effectué comme prévu.

Les participants perdus de vue doivent faire l’objet d’une recherche active de la part de l’investigateur.

- - 1. Participant inclus à tort

Un participant est considéré comme inclus à tort lorsqu’il a effectivement été inclus dans la recherche alors qu’il ne vérifiait pas tous les critères d’éligibilité. Les participants inclus à tort doivent faire l’objet d’une discussion en Conseil Scientifique. Ils doivent continuer à être suivis comme prévu par le protocole jusqu’à ce qu’une décision soit prise par le Conseil Scientifique.

- 1. Participation simultanée à d’autres recherches, période d’exclusion, indemnisation
- Les patients pourront participer simultanément à une autre recherche, sans période d’exclusion.
- Il n’y a pas d’indemnisation prévue

Gestion des ÉVÉNEMENTS INDÉSIRABLES, des grossesses et des faits nouveaux

- 1. Définitions

**Evénement indésirable** (article R1123-46 du code de la santé publique)

Toute manifestation nocive survenant chez une personne qui se prête à une recherche impliquant la personne humaine, que cette manifestation soit liée ou non à la recherche ou au produit sur lequel porte cette recherche.

**Effet indésirable** (article R1123-46 du code de la santé publique)

Evénement indésirable survenant chez une personne qui se prête à une recherche impliquant la personne humaine, lorsque cet événement est lié à la recherche ou au produit sur lequel porte cette recherche.

**Evénement ou effet indésirable grave** (article R1123-46 du code de la santé publique et guide ICH E2B)

Tout événement ou effet indésirable qui :

- entraîne la mort,
- met en danger la vie de la personne qui se prête à la recherche,
- nécessite une hospitalisation ou la prolongation de l'hospitalisation,
- provoque une incapacité ou un handicap important ou durable,
- se traduit par une anomalie ou une malformation congénitale,
- ou tout événement considéré médicalement grave,

et s'agissant du médicament, quelle que soit la dose administrée.

L’expression «mettre en danger la vie de la personne» est réservée à une menace vitale immédiate, au moment de l’événement indésirable.

**Effet indésirable inattendu pour les études hors produit de santé** (article R1123-46 du code de la santé publique)

Tout effet indésirable dont la nature, la sévérité ou l’évolution ne concorde pas avec les informations relatives aux produits, actes pratiqués et méthodes utilisées au cours de la recherche.

**Fait nouveau** (article R1123-46 du code de la santé publique)

- Toute nouvelle donnée pouvant conduire à une réévaluation du rapport des bénéfices et des risques de la recherche ou du produit objet de la recherche, à des modifications dans l’utilisation de ce produit, dans la conduite de la recherche, ou des documents relatifs à la recherche, ou à suspendre ou interrompre ou modifier le protocole de la recherche ou des recherches similaires.
- Pour les essais portant sur la première administration ou utilisation d’un produit de santé chez des personnes qui ne présentent aucune affection: tout effet indésirable grave.
  1. Description des événements indésirables attendus

Il n’y a pas d’événements indésirables spécifiques aux examens d’imagerie réalisés pour ce protocole.

Il s’agit des mêmes évènements indésirables propres à n’importe quel examen IRM avec injection de produit de contraste réalisé en routine clinique. tels que listés dans le résumé des caractéristiques du produit utilisé. Les évènements indésirables figurant dans la dernière version du résumé des caractéristiques du produit de contraste utilisé (disponible sur le site <https://base-donnees-publique.medicaments.gouv.fr/index.php>) [sont](https://base-donnees-publique.medicaments.gouv.fr/index.php) considérés comme attendus.

Il n’y a pas d’événements indésirables attendus en lien avec l’échographie.

Tout évènement indésirable ne figurant ni dans la liste des événements attendus ni dans la dernière version du document de référence du produit de contraste utilisé] est qualifié d’inattendu.

- 1. Conduite à tenir par l’investigateur en cas d’evenement indesirable, de fait nouveau ou de grossesse
     1. Recueil des évènements indésirables (EI)

L’investigateur est responsable du recueil des évènements indésirables qui surviennent entre la date de signature du consentement et la fin de participation du participant, soit au plus tard la veille de la chirurgie, le patient ayant alors réalisé les examens de la recherche et étant désormais pris en charge dans le cadre du soin courant.

Dans le cadre de ce protocole, l’investigateur rapporte les évènements indésirables cliniques et biologiques de grades ≥3 (selon échelle CTCAE version 5.0) dans le cahier d’observation.

Ces évènements indésirables sont recherchés à chaque visite au cours de l’étude par un interrogatoire et lors de l’examen clinique du participant.

**Exception au recueil :**

Les circonstances suivantes ne seront pas à recueillir :

- admission pour raison sociale ou administrative,
- hospitalisation prédéfinie par le protocole,
- hospitalisation pour traitement médical ou chirurgical programmé avant la recherche.
  - 1. Notification sans délai des évènements indésirables graves (EIG)et des faits nouveaux

L’investigateur évalue chaque événement indésirable au regard de sa gravité et de sa sévérité (échelle CTCAE version 5.0).

L’investigateur doit notifier à l’unité de sécurité et de vigilance par fax/mail, **sans délai** à compter du jour où il en a connaissance, tout EIG et tout fait nouveau.

Si l'investigateur prend connaissance d'un EIG, dont il suspecte un lien de causalité avec la recherche, survenant après la fin de la recherche chez un participant qu'il a traité, il en informe l’unité de sécurité et de vigilance sans délai.

**L’investigateur doit documenter au mieux l’événement, en donner si possible, le diagnostic médical.** L’investigateur doit s’assurer que les informations pertinentes de suivi soient communiquées l’unité de sécurité et de vigilance dès que possible.

L’investigateur doit transmettre, en plus du formulaire de notification des EIG, les copies des résultats de laboratoire ou des comptes rendus d’examens ou d’hospitalisation renseignant l’EIG, y compris les résultats négatifs pertinents **sans omettre de rendre ces documents anonymes** et d’inscrire le numéro et le code du participant.

**L’investigateur doit suivre le participant ayant présenté un EIG jusqu’à sa résolution, sa stabilisation à un niveau jugé par lui comme médicalement acceptable ou le retour à l’état antérieur, même si le participant a arrêté la procédure de la recherche.** Un complément d’information concernant l’évolution de l’événement, si elle n’est pas mentionnée dans le premier rapport, sera envoyé à l’unité de sécurité et de vigilance par l’investigateur.

L’investigateur et le promoteur doivent évaluer, indépendamment l’un de l’autre, le lien de causalité entre l’EIG, les traitements expérimentaux, les traitements associés et la recherche.

Tous les événements indésirables graves pour lesquels l’investigateur ou le promoteur estime qu’une relation de causalité peut être raisonnablement envisagée sont considérés comme des suspicions d’effets indésirables graves.

L’investigateur doit notifier tout fait nouveau dont il a connaissance au promoteur.

**Remarque :** En accord avec les articles R1413-67 (et suivant) et L1413-14, le promoteur rappelle que tout professionnel de santé ou tout représentant légal d’établissement de santé, d’établissement ou de service médico-social qui constate une infection nosocomiale ou un événement indésirable grave lié à des soins le déclare au directeur général de l’Agence régionale de santé. **Cette déclaration est indépendante de la déclaration des EIG au promoteur.**

- - 1. Notification des grossesses

La survenue d’une grossesse dans la période ou au décours immédiat d’une recherche, ne constitue pas un EIG. Cependant, si une femme débute une grossesse dans le cadre de la recherche, la grossesse doit être notifiée selon les mêmes modalités qu’un EIG car elle fera l’objet d’un suivi particulier jusqu’à son issue.

Pour cela l’investigateur informe l’unité de sécurité et de vigilance du promoteur grâce au formulaire de déclaration d’une grossesse.

L’investigateur doit suivre la participante jusqu’au terme de la grossesse ou de son interruption et en notifier l’issue à l’unité de sécurité et de vigilance. Toute anomalie constatée sur le fœtus ou l’enfant doit être notifiée. Toute interruption volontaire de grossesse (IVG), interruption médicale de grossesse (IMG) ou fausse couche doit faire l’objet d’une notification de grossesse, et si un critère de gravité est présent elle doit faire l’objet d’une notification d’EIG.

- - 1. Tableau récapitulatif du circuit de notification par type d’évènement

| **Type d’événement** | **Modalités de notification** | **Délai de notification à l’unité de sécurité et de vigilance** |
| --- | --- | --- |
| Evénement indésirable **non grave de grade ≥3** | Fiche « EI » du cahier d’observation | Pas de notification immédiate |
| Evénement indésirable **grave** | Fiche « EI » du cahier d’observation et Formulaire de déclaration d’EIG initiale  (+ Follow up si nécessaire)  + rapport écrit si nécessaire | **Notification sans délai** |
| Fait nouveau | Rapport écrit | **Notification sans délai** |
| Grossesse | Formulaire de déclaration d’une grossesse et cahier d’observation | **Dès confirmation de la grossesse** |

**Unité de sécurité et de vigilance de la recherche clinique-CHU de Bordeaux**

**Tél :** 05 57 82 16 26

**Fax :** 05 57 82 12 62

Courriel : [vigilance.essais-cliniques@chu-bordeaux.fr](mailto:vigilance.essais-cliniques@chu-bordeaux.fr)

- 1. Déclaration par le promoteur des effets indesirables graves inattendus, des faits nouveaux et autres evenements

L’unité de sécurité et de vigilance évalue si l’effet indésirable grave est attendu ou inattendu en se basant sur la liste des évènements indésirables graves attendus décrits dans le paragraphe 9.2 du protocole et sur le document de référence tel que défini dans le protocole.

Le promoteur/l’unité de vigilance déclare selon les délais en vigueur les informations de sécurité aux autorités compétentes et au CPP selon les exigences réglementaires spécifiques à chaque type d’essai.

L’unité de sécurité et de vigilance déclare aux autorités compétentes toute suspicion d’effet indésirable grave inattendu survenue en France et en dehors du territoire national et dans les délais suivants :

1) Dans le cas d’effet indésirable grave inattendu ayant entrainé la mort ou mis la vie en danger, sans délai à compter du jour où le promoteur en a eu connaissance,

2) Dans le cas des autres effets indésirables graves inattendus, au plus tard dans un délai de 15 jours à compter du jour où le promoteur en a connaissance.

L’unité de sécurité et de vigilance déclare sous forme d’un rapport de suivi à l’ANSM les informations complémentaires pertinentes concernant les effets indésirables graves inattendus :

- Dans le cas de suspicion d’effet indésirable grave inattendu ayant entrainé la mort ou mis la vie en danger, ces informations sont notifiées dans un délai de 8 jours à compter de la déclaration mentionnée au point 1)
- Dans les autres cas de suspicion d’effet indésirable grave inattendu et en cas de fait nouveau, les informations complémentaires pertinentes sont transmises dans un nouveau délai de 8 jours à compter du délai mentionné au point 2).

L’unité de sécurité et de vigilance déclare sans délai les faits nouveaux survenus au cours de la recherche :

- à l’ANSM,
- au Comité de Protection des Personnes.

Le promoteur et l’investigateur prennent les mesures urgentes appropriées. Le promoteur en informe sans délai l’autorité compétente et le comité de protection des personnes.

- 1. Rapport annuel de sécurité

A la date anniversaire de la première inclusion*,* l’unité de sécurité et de vigilance rédige un rapport de sécurité comprenant :

- la liste des effets indésirables graves susceptibles d’être liés au(x) traitement(s) expérimental(aux) de la recherche incluant les effets graves attendus et inattendus, survenus dans l’essai concerné pendant la période couverte par le rapport,
- une analyse concise et critique de la sécurité des participants se prêtant à la recherche.
- les tableaux de synthèse de tous les effets/événements indésirables graves survenus dans l’essai concerné depuis le début de la recherche

Ce rapport est envoyé à l’ANSM et au CPP dans les 60 jours suivant la date anniversaire de la première inclusion.

ASPECTS STATISTIQUES

- 1. Calcul de la taille d’étude

Il s’agit d’une étude exploratoire preuve de concept sans référence bibliographique similaire, la taille de l’étude a donc été fixé empiriquement à n = 50 patients. Il n’y a pas de base statistique préalable pour établir un effectif. Dans les études de SOPHiA Genetics, les tailles d’études sont fixées à 50 patients pour avoir un premier signal.

- 1. Méthodes statistiques employées

A l’issue des acquisitions d’imagerie et d’une étape de co-registration, nous obtiendrons pour chaque région d’intérêt (ROI) les variables suivantes :

- MR-élastographie : μMRE
- DWI-élastographie : ADCb200, ADCb1500, μDWI
- US-élastographie : μUS
- Scanner : d-, d+artériel, d+néphro, d+tardif

Nous aurons aussi un ensemble de variables possiblement confondantes liées au patient (âge, sexe, BMI, sarcopénie, DFG, hauteur de chaque rein rein, épaisseur du cortex de chaque rein, distance peau-mesure) et lié à la lésion (taille, volume, localisation antérieure/postérieure, polaire supérieure/équatoriale/inférieure, endophytique/exophytique)

Ainsi que le diagnostic final des lésions : bénin / malin, le type histologique, et pour les CCCR le grade histologique.

Toutes ces variables seront décrites en terme de nombre absolu et de pourcentages pour les variables catégorielles et en moyenne, écart-type, mediane, minimum, maximum et écart inter-quartile pour les variables numériques

- Les corrélations pour chaque paire de variables d’imagerie sur l’ensemble des ROIs seront évaluées par le test des rangs de Spearman.
- Le cas échéant, les relations entre μDWI, μMRE et μUS seront explorées de manière empirique (linéaire, logarithmique, quadratique, ... etc.)
- La répétabilité des mesures d’élasticité sera évaluée par la méthode de Bland-Altmann et par le coefficient de corrélation intra-classe (ICC)
- Les associations entre μDWI, μMRE et μUS, les variables patients et lésionnelles potentiellement confondantes seront étudiées, ainsi que les associations entre μDWI, μMRE et μUS et les caractéristiques cliniquement pertinentes des lésions rénales à l’aide de tests de Spearman (pour les couples de variables numériques), de tests de Mann-Whitney (pour les couples de variables catégorielle et numérique) et de tests de Chi-2 et de Fisher (pour les couples de variables catégorielles)
- La dernière partie consistera à développer un modèle prédictif de y = μMRE selon les variables X = d-, d+artériel, d+néphro, d+tardif. Nous disposerons de plusieurs observations par patients (lésion(s) rénale(s), parenchymes sains). Le patient et type de ROI (sain, tumoral) seront ajoutés comme co-variables dans le modèle.

L’échantillon sera splitté en un échantillon d’entrainement et un échantillon de test (70%/30% de la population). Plusieurs algorithmes de régression seront entraînés en cross-validation répétées (régression linéaire, régression linéaire pénalisée elasticnet, k-nearest neighbors, support vector machine, random forest, artificial neural network, ...) et sélectionnés selon la RMSE. Les performances des meilleurs modèles seront ensuite évaluées de manière indépendante sur l’échantillon de test (par nested cross-validation). Les analyses seront effectuées en collaboration entre l’équipe de SOPHiA Genetics et le Pr Crombé.

- 1. Plan d’analyse statistique

Un plan d’analyse statistique détaillé sera défini et fera l’objet d’une validation par le Conseil Scientifique de l’étude.

SURVEILLANCE DE La recherche

- 1. Conseil scientifique
     1. Composition

Il est composé des personnes suivantes : Dr Eva FOURAGE (investigateur principal), Dr Yann LE BRAS (co-investigateur), Pr Jean-Christophe BERNHARD (responsable scientifique), Pr. Amandine CROMBE (co-responsable scientifique), Dr Magalie CASTOREO (Pharmacien Unité de sécurité et vigilance), Pr Thierry COLIN (Centre de méthodologie), Pr Laura RICHERT (Méthodologiste), Solène RICARD et Manon JAFFREDO (Cheffes de projet), Julie DUMAS (ARC support investigateur), Guillaume HERMAN (Data manager), et un représentant du promoteur.

- - 1. Rythme des réunions

Le Conseil Scientifique de la recherche se réunit une fois par an.

- - 1. Rôles
- Il a pour mission de prendre toute décision importante à la demande de l’investigateur coordonnateur concernant la bonne marche de la recherche et le respect du protocole.
- Il vérifie le respect de l’éthique.
- Il s’informe auprès du Centre de Méthodologie et de Gestion des données et du centre investigateur coordonnateur de la recherche de l’état d’avancement de la recherche, des problèmes éventuels et des résultats disponibles.
- Il décide de toute modification pertinente du protocole nécessaire à la poursuite de la recherche, notamment :
  - - les mesures permettant de faciliter le recrutement dans la recherche,
    - les modifications au protocole avant leur présentation au CPP et à l’autorité de santé compétente,
    - les décisions d’ouvrir ou de fermer des sites participant à la recherche,
    - les mesures qui assurent aux personnes participant à la recherche la meilleure sécurité (dont modification des documents d’information et de recueil de consentement),
    - la discussion des résultats et la stratégie de publication de ces résultats.
- Le Conseil Scientifique peut proposer (après avis du Comité Indépendant) de prolonger ou d’interrompre la recherche en cas de rythme d’inclusion trop lent, d’un trop grand nombre de perdus de vue, de violations majeures du protocole ou bien pour des raisons médicales et/ou administratives. Il précise les modalités éventuelles du suivi prolongé des participants inclus dans la recherche.
- S’il est proposé de réaliser de nouvelles recherches biologiques à partir du matériel de la recherche par les personnes y participant et lorsqu’elles n’ont pas été prévues par le protocole, le Conseil Scientifique les étudie et définit les conditions d’information des participants, d’accès aux données et les règles de publication des résultats.
- A l’issue de la réunion, le président du Conseil Scientifique doit informer le promoteur des décisions arrêtées. Les décisions concernant une modification majeure ou une modification de budget doivent être approuvées par le promoteur.
  1. Comité indépendant de Surveillance

La conception de l'étude suit la prise en charge du patient, à l'exception de l'ajout d'une échographie, examen sans aucun risque, et d’une imagerie IRM avec injection de produit de contraste utilisé dans le cadre de son autorisation de mise sur le marché dont les risques sont mineurs si les contre-indications sont respectées. Nous considérons donc que la réalisation de ce projet de recherche ne justifie pas la constitution d'un comité indépendant de surveillance. Il pourrait cependant être mis en place au cours de la recherche en cas de survenue de problèmes de sécurité et/ou sur décision des investigateurs et/ou de l’unité de vigilance du promoteur.

GESTION ET TRAITEMENT DES données et documents source

- 1. Données et documents source

Les données source correspondent à l’ensemble des informations figurant dans des documents originaux, ou dans des copies authentifiées de ces documents, relatif aux examens cliniques, aux observations ou à d’autres activités menées dans le cadre d’une recherche et nécessaires à la reconstitution et à l’évaluation de la recherche. Les documents dans lesquels les données sources sont enregistrées sont appelés les documents sources.

Les documents sources utilisés seront le dossier médical informatisé du CHU de Bordeaux (DxCare®),
le réseau d’imagerie PACS du CHU de Bordeaux ainsi que des feuilles de recueil
de données dédiées et les résultats biologique sur DxCare. Elles correspondent aussi aux examens
d’imagerie ainsi que le compte rendu de ces examens.

- 1. Consignes pour le recueil des données

Toutes les informations requises par le protocole doivent être consignées sur les cahiers d’observation et une explication doit être apportée pour chaque donnée manquante.

Les données devront être recueillies au fur et à mesure qu'elles sont obtenues, et transcrites de façon nette et lisible.

Les données médicales seront collectées dans la base UroCCR.

- 1. Gestion et circuit des données
     1. Logiciel de gestion de données
        1. *Logiciel utilisé*

Le logiciel utilisé pour la gestion des données est un eCRF accessible à l’adresse suivante : <https://uroccr.fr>

La maintenance et le développement informatique de l’eCRF sont gérés par le CREDIM (Centre de Recherche et Développement en Informatique Médicale) qui est une plateforme informatique créée au sein de l'Université de Bordeaux.

- - - 1. *Hébergement des données*

Les données sont stockées sur un serveur dédié aux bases de données au sein du CHU de Bordeaux. Le système de gestion de base de données utilisé est Microsoft SQL server.

- - - 1. *Sécurité des données*

Le serveur se trouve dans une pièce dédiée, sans fenêtre. L’entrée dans le local sécurisé se fait au moyen d’un badge. Les portes du service sont sécurisées et fermées à clé le soir. Aucun ordinateur n’est en accès libre, l’authentification sur domaine est obligatoire.

La gestion des droits d’accès est gérée par le CREDIM pour les études du service.

Seuls les gestionnaires de la base, l’équipe projet et les auditeurs ont des droits d’accès direct à la base de données.

Les statisticiens et les data managers auront accès aux données pseudonymisées transmises via le serveur sécurisé NextCloud administré par la DSIN du CHU de Bordeaux.

- - 1. Saisie des données

La saisie des données est sous la responsabilité de l’investigateur du centre dans l’eCRF. Toute autre personne que l’investigateur effectuant la saisie dans l’eCRF doit être préalablement formée et déléguée par l’investigateur pour le faire.

- - 1. Codage des données

Les traitements prescrits et les événements cliniques sont codés dans l’eCRF afin de pouvoir effectuer le contrôle et l’analyse des données.

Les dictionnaires suivants sont utilisés pour le codage des termes médicaux :

- MedDRA (version en cours) FR/US

- ATC version

L’équipe investigatrice UroCCR est en charge du codage des données, sous responsabilité de l’investigateur*.*

- - 1. Contrôles des données

Des contrôles sont programmés afin de vérifier la cohérence et la complétude des données saisies dans l’eCRF. La liste des contrôles à mettre en place est définie conjointement entre l’investigateur coordonnateur et le responsable des analyses statistiques, dans le plan de validation des données de l’étude.

Le data manager d’UroCCR et l’ARC coordonnateur est responsable de la gestion des demandes de correction, qu’il lance régulièrement.

L’investigateur fait les corrections nécessaires à la résolution des demandes de corrections.

- - 1. Réconciliation des bases EI/EIG

Si nécessaire, le croisement des bases est effectué par l’Unité de Sécurité et de Vigilance (USV) du CHU de Bordeaux selon la procédure en vigueur. Le Data Manager est responsable de la transmission de la table des événements indésirables à l’USV. Les données sont exportées sous forme de fichier Excel qui est déposé sur la plateforme d'échanges sécurisés Nextcloud.

- - 1. Transfert des données

Pour des raisons de sécurité, les données d’imagerie ainsi que les données cliniques seront pseudonymisées et hébergées sur les serveurs sécurisés et contrôlés du CHU de Bordeaux pendant toute la durée de l’étude. Ces données (imagerie et données cliniques d’intérêts) pourront être transférées via la plateforme sécurisée NextCloud à la société SOPHiA Genetics pour la réalisation des analyses statistiques.

- - 1. Archivage de la base de données

L’archivage de la base de données est sous la responsabilité du promoteur de l’étude. Les données de
l’étude restent stockées dans le serveur de la DSI du CHU de Bordeaux, conformément à la
réglementation en vigueur pour l’étude. Une copie physique est conservée par le promoteur
conformément à la réglementation en vigueur.

- 1. Confidentialité des données

Conformément aux dispositions législatives en vigueur, les personnes ayant un accès direct aux données source prendront toutes les précautions nécessaires en vue d'assurer la confidentialité des informations relatives aux médicaments expérimentaux, aux recherches, aux personnes qui s'y prêtent et notamment en ce qui concerne leur identité ainsi qu’aux résultats obtenus. Ces personnes, au même titre que les investigateurs eux-mêmes, sont soumises au secret professionnel.

Pendant la recherche ou à son issue, les données recueillies sur les personnes qui s’y prêtent et transmises au promoteur par les investigateurs (ou tous autres intervenants spécialisés) seront pseudonymisées. Elles ne doivent en aucun cas faire apparaître en clair les noms des personnes concernées ni leur adresse.

Chaque participant se verra attribuer un code confidentiel d’identification composé d’un numéro de participant (3 chiffres).

Le promoteur s’assurera que chaque personne qui se prête à la recherche a donné son accord par écrit pour l’accès aux données individuelles la concernant et strictement nécessaires au contrôle de qualité de la recherche.

- 1. Conservation des documents et des donNees relatifs à la recherche

Les documents suivants relatifs à cette recherche sont archivés par l’investigateur conformément aux Bonnes Pratiques Cliniques, à l’arrêté du 11 août 2008 fixe la durée de conservation des documents relatifs aux recherches en santé et au règlement européen sur les médicaments:

- **pour une durée de 20 ans suivant la fin de la recherche**
  - Le protocole et les modifications éventuelles au protocole
    - Les cahiers d’observation (copies papiers ou électroniques)
    - Les dossiers source des participants ayant signé un consentement
    - Tous les autres documents et courriers relatifs à la recherche
    - L’exemplaire original des consentements éclairés signés des participants

Tous ces documents sont sous la responsabilité de l’investigateur pendant la durée réglementaire d’archivage.

Aucun déplacement ou destruction ne pourra être effectué sans l’accord du promoteur. Au terme de la durée réglementaire d’archivage, le promoteur sera consulté pour destruction. Toutes les données, tous les documents et rapports pourront faire l’objet d’audit ou d’inspection.

Les données recueillies pour l’étude seront accessibles aux personnes habilitées par le CHU de Bordeaux, pendant deux ans après la dernière publication des résultats de la recherche. Elles feront l’objet d’un archivage pendant 20ans après la fin de l’étude conformément à la réglementation en vigueur.

- 1. Cession des données

La gestion des données est supervisée par l’équipe d’urologie, en collaboration avec la sociéte SOPHiA Genetics. Les conditions de cession de tout ou partie de la base de données de la recherche sont décidées par le promoteur de la recherche et font l’objet d’un contrat écrit.

contrôle et assurance qualité

- 1. Accès aux données

L’acceptation de la participation au protocole implique que les investigateurs mettront à disposition les documents et données individuelles strictement nécessaires au suivi, au contrôle de qualité et à l’audit de la recherche, à la disposition des personnes ayant un accès à ces documents conformément aux dispositions législatives et réglementaires en vigueur.

- 1. Contrôle Qualité

Le contrôle qualité sera effectué par un attaché de recherche clinique mandaté par le promoteur conformément au plan de monitorage basé sur le risque (participant, logistique, impact, ressources) défini pour la recherche.

Ce dernier défini la nature des éléments à vérifier, les modalités et le rythme de visite dans le centre investigateur.

Toute visite fera l’objet d’un rapport de monitorage par compte-rendu écrit transmis à l’investigateur principal du centre.

- 1. Audit et inspection

Un audit peut être réalisé à tout moment par des personnes mandatées par le [promoteur et](http://www.chusa.jussieu.fr/urcest/sous_cadre.php?fich=Lexique/new_index.php?isphp=0&fich=EC/legislation/DispositionslegislativesPromoteur.htm) indépendantes des personnes menant la recherche. Il a pour objectif de vérifier la sécurité des participants et le respect de leurs droits, le respect de la réglementation applicable et la fiabilité des données s

Une inspection peut également être diligentée par une autorité compétente (ANSM pour la France ou autre autorité réglementaire dans le cadre d’une recherche européenne par exemple).

L’audit, aussi bien que l’inspection, pourront s’appliquer à tous les stades de la recherche, du développement du protocole à la publication des résultats et au classement des données utilisées ou produites dans le cadre de la recherche.

Les investigateurs acceptent de se conformer aux exigences du promoteur en ce qui concerne un audit et à l’autorité compétente pour une inspection de la recherche.

Considérations éthiques et réglementaires

- 1. Conformité aux textes de référence

Le promoteur et l’(es) investigateur(s) s’engagent à ce que cette recherche soit réalisée en conformité avec la loi n°2012-300 du 5 mars 2012 relative aux recherches impliquant la personne humaine, ainsi qu’en accord avec les Bonnes Pratiques Cliniques (I.C.H. E6 (R2) du 1er décembre 2016 et décision du 24 novembre 2006) et la déclaration d’Helsinki (qui peut être retrouvée dans sa version intégrale sur le site [www.wma.net](https://www.wma.net/fr/ce-que-nous-faisons/ethique/declaration-dhelsinki/)).

La recherche est conduite conformément au présent protocole. Hormis dans les situations d’urgence nécessitant la mise en place d’actes thérapeutiques précis, l’(es) investigateur(s) s’engage(nt) à respecter le protocole en tous points en particulier en ce qui concerne le recueil du consentement et la notification et le suivi des événements indésirables graves.

Cette recherche a reçu l’avis favorable du Comité de Protection des Personnes (CPP) IdF1 et l’autorisation de l’ANSM.

Le CHU de Bordeaux promoteur de cette recherche, a souscrit un contrat d’assurance en responsabilité civile auprès de Lloyd’s Insurance Company SA (représentée par BEAH, mandataire) conformément aux dispositions du code de la santé publique.

Les données nécessaires à cette recherche sont enregistrées dans la base de données UroCCR qui a obtenu l’autorisation de la Commission Nationale de l'Informatique et des Libertés (CNIL) en date du 12/04/2013 (demande d’autorisation n°912578, décision DR-2013-206). Les données de la base UroCCR font l’objet d’un traitement informatisé au CREDIM dans le respect de la loi n°78-17 du 6 janvier 1978 relative à l’informatique, aux fichiers et aux libertés modifiée par la loi 2004-801 du 6 août 2004.

Cette recherche entre dans le cadre de la « Méthodologie de référence » MR-001 en application des dispositions de l’article 54 de la loi du 6 janvier 1978 modifiée relative à l’information, aux fichiers et aux libertés. Le CHU de Bordeaux a signé un engagement de conformité à cette « Méthodologie de référence ».

Cette recherche est enregistrée dans la base ID-RCB sous le n° 2024-A00959-38.

Cette recherche est enregistrée sur le site http://clinicaltrials.gov/

- 1. Modifications au protocole

Toute modification substantielle, c’est à dire toute modification de nature à avoir un impact significatif sur la protection des personnes, sur les conditions de validité et sur les résultats de la recherche, sur la qualité et la sécurité des produits expérimentés, sur l’interprétation des documents scientifiques qui viennent appuyer le déroulement de la recherche ou sur les modalités de conduite de celle-ci, fait l’objet d’un amendement écrit qui est soumis au promoteur ; celui-ci doit obtenir, préalablement à sa mise en œuvre, un avis favorable du CPP et, le cas échéant, une autorisation de l’ANSM.

Les modifications non substantielles, c'est à dire celles n’ayant pas d’impact significatif sur quelque aspect de la recherche que ce soit, sont communiquées au CPP à titre d’information.

Toutes les modifications sont validées par le promoteur, et par tous les intervenants de la recherche concernés par la modification, avant soumission au CPP et, le cas échéant, à l’ANSM. Cette validation peut nécessiter la réunion de tout comité constitué pour la recherche.

Toutes les modifications au protocole doivent être portées à la connaissance de tous les investigateurs qui participent à la recherche. Les investigateurs s’engagent à en respecter le contenu.

Toute modification qui modifie la prise en charge des participants ou les bénéfices, risques et contraintes de la recherche fait l’objet d’une nouvelle note d’information et d’un nouveau formulaire de consentement dont le recueil suit la même procédure que celle précitée.

Rapport final

Dans un délai d'un an suivant la fin de la recherche ou son interruption, un rapport final sera établi et signé par le promoteur et l'investigateur. Ce rapport sera tenu à la disposition de l'autorité compétente. Le promoteur transmettra au CPP et, le cas échéant, à l'ANSM les résultats de la recherche sous forme d'un résumé du rapport final dans un délai d'un an après la fin de la recherche.

Regles relatives à la publication

- 1. Communications scientifiques

L’analyse des données fournies par le centre investigateur est réalisée par la société SOPHiA Genetics. Cette analyse donne lieu à un rapport écrit qui est soumis au promoteur, qui transmettra au Comité de Protection des Personnes et à l’autorité compétente.

Toute communication écrite ou orale des résultats de la recherche doit recevoir l’accord préalable de l’investigateur coordonnateur et, le cas échéant, de tout comité constitué pour la recherche.

L’investigateur coordonnateur s’engage à mettre à disposition du public les résultats de la recherche aussi bien négatifs et non concluants que positifs.

Pour la publication des résultats principaux, il faudra mentionner le nom du promoteur, de tous les investigateurs ayant inclus ou suivi des participants dans la recherche, des méthodologistes, biostatisticiens et data managers ayant participé à la recherche, des membres du(des) comité(s) constitué(s) pour la recherche, la participation de la société Sophia Genetics et la mention « *ce travail a bénéficié d’une aide de l’Etat gérée par l’Agence Nationale de la Recherche au titre du troisième PIA intégré à France 2030 portant la référence ANR-21-RHUS-0015*». Il sera tenu compte des règles internationales d’écriture et de publication (The Uniform Requirements for Manuscripts de l’ICMJE, avril 2010).

- 1. Communication des résultats aux participants

Conformément à la loi n°2002-303 du 4 mars 2002, les participants sont informés, à leur demande, des résultats globaux de la recherche.

Références Bibliographiques

1. Siegel RL, Miller KD, Fuchs HE, Jemal A. Cancer Statistics, 2021. CA Cancer J Clin. janv 2021;71(1):7‑33.

2. Escudier B, Porta C, Schmidinger M, Rioux-Leclercq N, Bex A, Khoo V, et al. Renal cell carcinoma: ESMO Clinical Practice Guidelines for diagnosis, treatment and follow-up†. Ann Oncol Off J Eur Soc Med Oncol. 1 mai 2019;30(5):706‑20.

3. Frank RA, Dawit H, Bossuyt PMM, Leeflang M, Flood TA, Breau RH, et al. Diagnostic Accuracy of MRI for Solid Renal Masses: A Systematic Review and Meta-analysis. J Magn Reson Imaging JMRI. avr 2023;57(4):1172‑84.

4. Schieda N, Davenport MS, Silverman SG, Bagga B, Barkmeier D, Blank Z, et al. Multicenter Evaluation of Multiparametric MRI Clear Cell Likelihood Scores in Solid Indeterminate Small Renal Masses. Radiology. juin 2022;303(3):590‑9.

5. Bauman TM, Potretzke AM, Wright AJ, Knight BA, Vetter JM, Figenshau RS. Partial Nephrectomy for Presumed Renal-Cell Carcinoma: Incidence, Predictors, and Perioperative Outcomes of Benign Lesions. J Endourol. avr 2017;31(4):412‑7.

6. Cornelis F, Tricaud E, Lasserre AS, Petitpierre F, Bernhard JC, Le Bras Y, et al. Routinely performed multiparametric magnetic resonance imaging helps to differentiate common subtypes of renal tumours. Eur Radiol. mai 2014;24(5):1068‑80.

7. Kay FU, Canvasser NE, Xi Y, Pinho DF, Costa DN, Diaz de Leon A, et al. Diagnostic Performance and Interreader Agreement of a Standardized MR Imaging Approach in the Prediction of Small Renal Mass Histology. Radiology. mai 2018;287(2):543‑53.

8. Vendrami CL, Velichko YS, Miller FH, Chatterjee A, Villavicencio CP, Yaghmai V, et al. Differentiation of Papillary Renal Cell Carcinoma Subtypes on MRI: Qualitative and Texture Analysis. AJR Am J Roentgenol. déc 2018;211(6):1234‑45.

9. Zhang J, Yu Y, Liu X, Tang X, Xu F, Zhang M, et al. Evaluation of Renal Fibrosis by Mapping Histology and Magnetic Resonance Imaging. Kidney Dis Basel Switz. mars 2021;7(2):131‑42.

10. Güven AT, Idilman IS, Cebrayilov C, Önal C, Kibar MÜ, Sağlam A, et al. Evaluation of renal fibrosis in various causes of glomerulonephritis by MR elastography: a clinicopathologic comparative analysis. Abdom Radiol N Y. janv 2022;47(1):288‑96.

11. Prezzi D, Neji R, Kelly-Morland C, Verma H, OʼBrien T, Challacombe B, et al. Characterization of Small Renal Tumors With Magnetic Resonance Elastography: A Feasibility Study. Invest Radiol. juin 2018;53(6):344‑51.

12. Rouvière O, Souchon R, Pagnoux G, Ménager JM, Chapelon JY. Magnetic resonance elastography of the kidneys: feasibility and reproducibility in young healthy adults. J Magn Reson Imaging JMRI. oct 2011;34(4):880‑6.

13. Le Bihan D, Ichikawa S, Motosugi U. Diffusion and Intravoxel Incoherent Motion MR Imaging-based Virtual Elastography: A Hypothesis-generating Study in the Liver. Radiology. nov 2017;285(2):609‑19.

14. Toffoli T, Saut O, Etchegaray C, Jambon E, Le Bras Y, Grenier N, et al. Differentiation of Small Clear Renal Cell Carcinoma and Oncocytoma through Magnetic Resonance Imaging-Based Radiomics Analysis: Toward the End of Percutaneous Biopsy. J Pers Med. 28 sept 2023;13(10):1444.

15. Cornelis F, Grenier N. Multiparametric Magnetic Resonance Imaging of Solid Renal Tumors: A Practical Algorithm. Semin Ultrasound CT MR. févr 2017;38(1):47‑58.

16. Cornelis F, Tricaud E, Lasserre AS, Petitpierre F, Bernhard JC, Le Bras Y, et al. Multiparametric magnetic resonance imaging for the differentiation of low and high grade clear cell renal carcinoma. Eur Radiol. janv 2015;25(1):24‑31.
