# Supplementary material for: Multimodal imaging to analyze the biomechanical properties of kidney tumors, evaluating feasibility, inter-modality correspondence, and diagnostic value (UroCCR-115)
Source: PLoS One. 2026 Jul 8;21(7):e0351477. doi: 10.1371/journal.pone.0351477 (PMC13345387; doi:10.1371/journal.pone.0351477)
Supplement: S2 File — (DOCX) [file pone.0351477.s002.docx]

**Multimodal imaging of the biomechanical properties of renal tumors: feasibility study, intermodality correlation of elasticity values, and diagnostic value (UroCCR-115).
Kidney 3D-Print Mechanics**

**Sponsor code:** CHUBX 2022/11

**INTERVENTIONAL RESEARCH PROTOCOL INVOLVING HUMAN SUBJECTS (Category 1 – HPS)**

Version n°2.0 du 16/10/2025

**Numéro ID-RCB** : 2024-A00959-38

# This research received funding from the ANR as part of the RHU Digital Urology 3D program.

Promoteur :

**Centre Hospitalier Universitaire de Bordeaux**

12, rue Dubernat

33400 Talence FRANCE

Investigateur principal :

**Dr Eva JAMBON**

Imagerie diagnostique et interventionnelle de l’adulte CHU Bordeaux - Hôpital Pellegrin

Place Amélie Raba Léon 33076 Bordeaux Cedex

Tel : 05 56 79 59 88 – Fax : 05 57 82 16 50

Mail : [eva.jambon@chu-bordeaux.fr](mailto:eva.jambon@chu-bordeaux.fr)

Responsable scientifique :

**Pr Jean-Christophe BERNHARD**

CHU de Bordeaux - Hôpital Pellegrin Place Amélie Rabat Léon

33076 Bordeaux Cedex

Tel : 05 57 82 06 87 – Fax : 05 56 79 56 51

Mail : [jean-christophe.bernhard@chu-bordeaux.fr](mailto:jean-christophe.bernhard@chu-bordeaux.fr)

Centre de Méthodologie :

**Pr Thierry COLIN**

SOPHiA GENETICS

Technopôle Izarbel 374 Allée d’Abbadie

64210 Bidart

Mail: [TColin@sophiagenetics.com](mailto:TColin@sophiagenetics.com)

Unité de sécurité et de vigilance de la recherche clinique :

**Dr Caroline LACOSTE**

Direction de la Recherche Clinique et de l’Innovation 12, rue Dubernat

33404 Talence Cedex

Tel : 05 57 82 16 26 – Fax : 05 57 82 12 62

Mail : [vigilance.essais-cliniques@chu-bordeaux.fr](mailto:vigilance.essais-cliniques@chu-bordeaux.fr)

**This protocol was designed and drafted based on version 4.0 dated 18/01/2022 of the GIRCI SOHO template protocol.**

**HISTORIQUE DES MISES A JOUR DU PROTOCOLE**

| Version | Date | Raison de la mise a jour |
| --- | --- | --- |
| 1.0 | 16/04/2024 | Initial version submitted to the CPP |
| 1.1 | 07/06/2024 | Version revised following the comments of the ANSM |
| 1.2 | 26/06/2024 | Version revised following the comments of the CPP dated 18/06/2024 |
| 1.3 | 22/07/2024 | Version revised following the comments of the CPP dated 18/07/2024 |

**PAGE DE SIGNATURE DU PROTOCOLE**

**Multimodal imaging of the biomechanical properties of renal tumors: feasibility study, intermodality correspondence of elasticity values, and diagnostic value (UroCCR-115).**

**Kidney 3D-Print Mechanics**

**Promoter code :** CHUBX 2022/18

**Promoteur**

Centre Hospitalier Universitaire de Bordeaux at Talence, the : 12, rue Dubernat

33400 Talence

**The Acting Chief Executive Officer of Bordeaux University Hospital**
 A. THOMAS

**And by delegation, the Director of Clinical Research and Innovation**
 G. DULUC

à Bordeaux, le *:*

### Investigateur principal Dr Eva JAMBON

Imagerie diagnostique et interventionnelle de l’adulte CHU de Bordeaux – Hôpital Pellegrin

Place Amélie Raba Léon 33076 Bordeaux Cedex

Tel : 05 56 79 59 88 – Fax : 05 57 82 16 50

Mail : [eva.jambon@chu-bordeaux.fr](mailto:eva.jambon@chu-bordeaux.fr)

**MAIN CONTACTS**

### Principal Investigator

Dr Eva JAMBON

Diagnostic and interventional imaging in adults, CHU Bordeaux

Place Amélie Raba Léon 33076 Bordeaux Cedex

Tel : 05 56 79 59 88 – Fax : 05 57 82 16 50

Mail : [eva.jambon@chu-bordeaux.fr](mailto:eva.jambon@chu-bordeaux.fr)

### Co-investigator

Dr Yann LE BRAS

Mail : [yann.lebras@chu-bordeaux.fr](mailto:yann.lebras@chu-bordeaux.fr)

### Scientific Responsable

Pr Jean-Christophe BERNHARD CHU de Bordeaux - Hôpital Pellegrin Place Amélie Rabat Léon

33076 Bordeaux Cedex

Tel : 05 57 82 06 87 – Fax : 05 56 79 56 51

Mail : [jean-christophe.bernhard@chu-bordeaux.fr](mailto:jean-christophe.bernhard@chu-bordeaux.fr)

### Co-responsables scientifiques

Pr Amandine CROMBE

Mail : [amandine.crombe@chu-bordeaux.fr](mailto:amandine.crombe@chu-bordeaux.fr) Dr Gaëlle MARGUE

Mail : [gaelle.margue@chu-bordeaux.fr](mailto:gaelle.margue@chu-bordeaux.fr)

Sylvie GEORGEVAIL (Attachée de recherche clinique) Valérie MARTY (Chargée de vigilance)

Direction de la recherche clinique et de l’innovation 12 rue Dubernat

33404 Talence Cedex

Tel: 05 57 82 16 26 - Fax: 05 57 82 12 62

[vigilance.essais-cliniques@chu-bordeaux.fr](mailto:vigilance.essais-cliniques@chu-bordeaux.fr)

### Promoter

Centre Hospitalier Universitaire de Bordeaux 12 rue Dubernat

33400 Talence FRANCE

### Person Responsible for Research at the Sponsor Level

M. Gilles DULUC – Directeur de la Recherche Clinique et de l’Innovation

Mail : [gilles.duluc@chu-bordeaux.fr](mailto:gilles.duluc@chu-bordeaux.fr)

Dr Anne GIMBERT – Responsable « Promotion interne » Direction de la Recherche Clinique et de l’Innovation du CHU de Bordeaux

12 rue Dubernat

33404 Talence Cedex

Tel : 05 57 82 08 34 – Fax : 05 56 79 49 26

Mail : [anne.gimbert@chu-bordeaux.fr](mailto:anne.gimbert@chu-bordeaux.fr)

### ARC support investigator

Mme Joséphine GAY

Service d’Urologie et Transplantation Rénale CHU de Bordeaux – Pellegrin

Tel : 05 57 82 23 94 – Fax : 05 56 79 56 51

Mail : [josephine.gay@chu-bordeaux.fr](mailto:josephine.gay@chu-bordeaux.fr)

### Data Manager UroCCR

M. Guillaume HERMAN

Service d’Urologie et Transplantation Rénale CHU de Bordeaux - Pellegrin

Tel : 05 56 79 60 50 – Fax : 05 56 79 56 51

Mail : [guillaume.herman@chu-bordeaux.fr](mailto:guillaume.herman@chu-bordeaux.fr)

### Unit for Methodological Support in Clinical and Epidemiological Research

Pr Laura RICHERT

Service d'information médicale, Pôle Santé publique CHU Bordeaux

146 rue Léo Saignat, case n°75 33076 Bordeaux Cedex

Tel : 05 57 57 11 29 / 14 42 - Fax : 05 57 57 15 78

Mail : [laura.richert@chu-bordeaux.fr](mailto:laura.richert@chu-bordeaux.fr)

### Clinical Study Manager

Mme Corinne CASTERMANS

Direction de la Recherche Clinique et de l’Innovation du CHU de Bordeaux

12 rue Dubernat

33404 Talence Cedex

Tel : 05 57 82 08 53– Fax : 05 56 79 49 26

Mail : [corinne.castermans@chu-bordeaux.fr](mailto:corinne.castermans@chu-bordeaux.fr)

**Statistic Centre**

Pr Thierry COLIN SOPHiA GENETICS

Technopôle Izarbel 374 Allée d’Abbadie

64210 Bidart

Email: [TColin@sophiagenetics.com](mailto:TColin@sophiagenetics.com)

**Clinical Research Monitoring Unit** Dr Caroline LACOSTE (Médecin responsable) Dr Marine ROUSSET (Pharmacien évaluateur)

Dr Magalie CASTOREO (Pharmacien évaluateur)

**TABLE OF CONTENTS**

1. [RESEARCH SUMMARY 9](#_TOC_250064)

[ABSTRACT 14](#_TOC_250063)

1. [SCIENTIFIC JUSTIFICATION AND GENERAL DESCRIPTION 17](#_TOC_250062)
   1. [CURRANT STATE OF KNOWLEDGE 17](#_TOC_250061)
      1. On the pathology 17
      2. On standard and study procedures 17
   2. [research hypotheses and expected results 18](#_TOC_250060)
   3. [benefit/risk ratio 18](#_TOC_250059)
   4. [expected outcomes 19](#_TOC_250058)
2. [RESEARCH OBJECTIVES 19](#_TOC_250057)
   1. [PRIMARY OBJECTIVE 19](#_TOC_250056)
   2. [SECONDARY OBJECTIVES 19](#_TOC_250055)
3. [ENDPOINTS 20](#_TOC_250054)
   1. [PRIMARY ENDPOINT 20](#_TOC_250053)
   2. [SECONDARY ENDPOINT 20](#_TOC_250052)
4. [STUDY DESIGN 21](#_TOC_250051)
   1. [JUSTIFICATION OF METHODOLOGICAL CHOICES 21](#_TOC_250050)
   2. [STUDY SCHEME 21](#_TOC_250049)
5. [ELEGIBILITY CRITERIA 22](#_TOC_250048)
   1. [INCLUSION CRITERIA 22](#_TOC_250047)
   2. [NON INCLUSION CRITERIA 22](#_TOC_250046)
   3. [FEASIBILITY AND RECRUITMENT PROCEDURES 22](#_TOC_250045)
6. [STUDY PROCEDURE(S) 23](#_TOC_250044)
   1. [EXPERIMENTAL PROCEDURE(S) 23](#_TOC_250043)
      1. Ultrasound elastography using the ARFI method 23
      2. MRI elastography using DWI-E and MRE methods 23
   2. [COMPARISON PROCEDURE 23](#_TOC_250042)
   3. [Post-PROCESSING OF ACQUIRED IMAGING 24](#_TOC_250041)
7. [STUDY CONDUCT 25](#_TOC_250040)
   1. [STUDY TIMELINE 25](#_TOC_250039)
   2. [SUMMARY TABLE OF PARTECIPANT FOLLOW-UP 26](#_TOC_250038)
   3. [T0 : PRE-INCLUSION/INCLUSION VISIT 26](#_TOC_250037)
      1. Informed consent collection 26
      2. Visit procedures 27
   4. [T1_ IMAGING ACQUISITION 27](#_TOC_250036)
   5. T2 _ MECHANICAL TESTS ON FRESG SURGICAL SPECIMEN (R) 27
   6. [FOLLOW UP VISIT 27](#_TOC_250035)
   7. [END OF STUDY VISIT 28](#_TOC_250034)
   8. [STOPPING RULES 28](#_TOC_250033)
      1. Discontinuation of a partecipant’s involvement in the study 28
      2. Study termination 28
   9. [PROTOCOL DEVIATIONS 29](#_TOC_250032)
      1. Early and definitive discontinuation of the study procedure 29
      2. Participant lost follow-up 29
      3. Participant included in error 29

**9 SIMULTANEOUS PARTICIPATION IN OTHER STUDIES, WASHOUT PERIOD, COMPENSATION**  29

1. **MANAGEMENT OF ADVERSE EVENTS, PREGNANCIES, AND NEW INFORMATION** 29
   9.1. **DEFINITIONS**  29
   9.2. **DESCRIPTION OF EXPECTED ADVERSE EVENTS**  30
   9.3. **INVESTIGATOR ACTIONS IN THE EVENT OF AN ADVERSE EVENT, NEW INFORMATION, OR PREGNANCY**  30
   9.3.1. Collection of adverse events (AEs) 30
   9.3.2. Immediate reporting of serious adverse events (SAEs) and new information 30
   9.3.3. Reporting of pregnancies 31
   9.3.4. Summary table of the reporting pathway by event type 31

9.4. **SPONSOR REPORTING OF UNEXPECTED SERIOUS ADVERSE EFFECTS, NEW INFORMATION, AND OTHER EVENTS**  32
9.5. **ANNUAL SAFETY REPORT**  32

1. **STATISTICALCONSIDERATIONS**  34
   10.1. **SAMPLE SIZE CALCULATION**  34
   10.2. **STATISTICAL METHODS USED**  34
   10.3. **STATISTICAL ANALYSIS PLAN**  34
2. **STUDY OVERSIGHT**  35
   11.1. **SCIENTIFIC ADVISORY BOARD**  35
   11.1.1. Composition 35
   11.1.2. Meeting schedule 35
   11.1.3. Roles 35
   11.2. **INDEPENDENT DATA MONITORING COMMITTEE**  35
3. **DATA AND SOURCE DOCUMENT MANAGEMENT**  36
   12.1. **DATA AND SOURCE DOCUMENTS**  36
   12.2. **DATA COLLECTION GUIDELINES**  36
   12.3. **DATA MANAGEMENT AND FLOW**  36
   12.3.1. Data management software 36
   12.3.1.1. Software used 36
   12.3.1.2. Data hosting 36
   12.3.1.3. Data security 36
   12.3.2. Data entry 36
   12.3.3. Data coding 37
   12.3.4. Data validation and checks 37
   12.3.5. Reconciliation of AE/SAE databases 37
   12.3.6. Data transfer 37
   12.3.7. Database archiving 37

12.4. **DATA CONFIDENTIALITY**  37
12.5. **RETENTION OF STUDY DOCUMENTS AND DATA**  38
12.6. **DATA TRANSFER / SHARING.**  38

1. **QUALITY CONTROL AND QUALITY ASSURANCE**  38
   13.1. **ACCESS TO DATA**  38
   13.2. **QUALITY CONTROL**  38
   13.3. **AUDIT AND INSPECTION** 38

**14 ETHICAL AND REGULATORY CONSIDERATIONS**  39

**14.1 COMPLIANCE WITH REFERENCE TEXT**

- 1. COMPLIANCE WITH REFERENCE TEXTS  [39](#_TOC_250005)

14.2 [AMENDMENTS TO THE PROTOCOL 39](#_TOC_250004)

1. [FINAL REPORT 40](#_TOC_250003)
2. [REGLES RELATIVES A LA PUBLICATION 40](#_TOC_250002)
   1. SCIENTIFIC COMMUNICATIONS  [40](#_TOC_250001)

16.2. COMMUNICATIONS OF THE RESULTS TO THE PARTECIPANTS. 40

REFERENCES BIBLIOGRAPHIQUES 41

**LIST OF ABBREVIATIONS**

**ADC:** Apparent Diffusion Coefficient

**ANSM:** French National Agency for the Safety of Medicines and Health Products

**ARFI:** Acoustic Radiation Force Impulse

**BMI**: Body Mass Index

**CCCR:** Clear Cell Renal Carcinoma

**REDIM:** Research and Development Center for Medical Informatics

**DFG:** Glomerular Filtration Rate

**DWI:** Diffusion Weighted Imaging

**DWI-E:** DWI Elastography

**e-CRF:** Electronic Case Report Form

**EI:** Adverse Event

**EIG:** Serious Adverse Event

**ICC:** Intraclass Correlation Coefficient

**ICH:** International Council for Harmonisation

**IRM/MRI:** Magnetic Resonance Imaging

**IRM-mp:** Multi-Parametric MRI

**MR:** Magnetic Resonance

**MRE:** Magnetic Resonance Elastography

**MSE:** Mean Squared Error

**OMS:** World Health Organization

**R3DP-M:** Rein 3D Print-Mechanics Study

**RCP:** Multidisciplinary Consultation Meeting

**RMSE:** Root Mean Square Error

**ROI:** Region of Interest

**US:** Ultrasound (Echography)

Let me know if you need further translation or assistance!

# RESEARCH SUMMARY

| **SPONSOR** | Centre Hospitalier Universitaire de Bordeaux 12, rue Dubernat  33400 Talence FRANCE |
| --- | --- |
| **PRINCIPAL INVESTIGATOR** | Dr Eva JAMBON  Imagerie diagnostique et interventionnelle de l’adulte CHU Bordeaux – Hôpital Pellegrin  Place Amélie Raba Léon, 33076 Bordeaux Cedex  Tel : 05 56 79 59 88  Mail : [eva.jambon@chu-bordeaux.fr](mailto:eva.jambon@chu-bordeaux.fr) |
| **SCIENTIFIC DIRECTOR OR PRINCIPAL SCIENTIST** | Pr Jean-Christophe BERNHARD  Service de Chirurgie Urologique et Transplantation CHU de Bordeaux – Hôpital Pellegrin  Place Amélie Raba Léon 33076 Bordeaux Cedex  Tel : 05 57 82 03 50  Mail : [jean-christophe.bernhard@chu-bordeaux.fr](mailto:jean-christophe.bernhard@chu-bordeaux.fr) |
| **TITLE** | **3D-Printed Kidney Mechanics** **Multimodal Imaging of Biomechanical Properties of Renal Tumors: Feasibility Study, Intermodality Correspondence of Elasticity Values, and Diagnostic Value**  This seems to describe a study or research focusing on the biomechanical properties of kidney tumors using different imaging modalities. The study explores how different imaging techniques correspond to each other in measuring the elasticity of kidney tumors, and evaluates their diagnostic potential. The reference to "3D-print" might suggest the use of 3D printed models to better visualize and study the mechanical properties of kidney tissues or tumors. |
| **Justification / context** | **Medical Imaging in Renal Carcinomas: Role, Modalities, and the Potential for 3D Printing**  Medical imaging plays a central role in the diagnostic and therapeutic management of renal carcinomas. It is essential for confirming the presence of a tumor, localizing it, assessing its malignancy, and even identifying the histological subtype. Imaging also guides biopsies, helps with TNM staging, aids in surgical planning, monitors the effectiveness of systemic treatments, guides ablation procedures, and detects recurrences after curative treatments.  Imaging of renal tumors relies on three complementary modalities: ultrasound (US), which is based on sound waves; computed tomography (CT), which uses X-rays; and magnetic resonance imaging (MRI), which is based on nuclear magnetic resonance.  The most commonly performed imaging test is the CT scan, which is also used to prepare 3D impressions. However, the correlation between the density of renal parenchyma and renal tumors (before and at various acquisition times after contrast injection) on CT, and the elasticity parameters measured by ultrasound and MRI, has never been explored. Being able to accurately predict tissue stiffness based on CT scans could enhance the predictive performance of CT and the quality of 3D renal tumor prints.  This innovative project aims to evaluate the correspondence between different imaging modalities for characterizing the elasticity of both healthy and pathological renal tissue. It will also improve the realism of 3D prints used by urological surgeons and identify new complementary imaging biomarkers. |
| **OBJECTIVES** | The primary objective of this project is to **develop a predictive model** for the biomechanical properties of normal and pathological renal tissue, evaluated using the reference method (MR-elastography), based on the densities obtained during the different phases of the CT scan. These predictive models will be evaluated using several indices (R², MSE, RMSE), and the best-performing models will be selected based on the RMSE (Root Mean Square Error). This evaluation will be conducted prior to the definitive histological diagnosis.  This model aims to **integrate imaging data from CT scans** with biomechanical properties measured by MR-elastography to provide a more accurate, non-invasive means of assessing renal tissue. By doing so, we aim to improve the predictive accuracy for distinguishing between healthy and pathological renal tissues and to assist in better treatment planning, particularly in terms of preoperative assessments and prediction of tumor behavior. |

|  | Secondary Objectives:  1. Voxel-to-Voxel, ROI-to-ROI, and Anatomical Region-to-Region Correlation:  Correlate voxel by voxel, ROI (Region of Interest) by ROI, and anatomical region by anatomical region the elasticity of renal tumors and surrounding healthy renal tissue obtained by MR-elastography, DWI-elastography, and US-elastography with density values from the different phases of uro-CT scans.  2. Feasibility and Parameterization of DWI-Elastography on a Clinical 1.5-Tesla MRI:  - Evaluate the quality (using a 5-point ordinal qualitative scale),  - Contrast (calculating the contrast-to-noise ratio),  - Noise (calculating the signal-to-noise ratio),  - Artifacts (annotation of encountered artifacts).   1. Feasibility of MR-Elastography, DWI-Elastography, and US-Elastography for Measuring Elasticity in Renal Parenchyma and Tumors:  - Identify limiting factors (patient morphotype [BMI], sarcopenia, location, size, and architecture of the lesion), - Count situations where the examination has no diagnostic clinical value and correlate with potential limiting factors.   4. Evaluate the Repeatability of MR-Elastography, DWI-Elastography, and US-Elastography on Renal Parenchyma and Tumors:   - Assess repeatability across the entire sample, considering patient morphotype, lesion location, size, and architecture, - Calculate intra-class correlation coefficients and plot Bland-Altman graphs.   5.Identify Decorrelation or Non-Correlation and Potential Biases:**   - Identify situations of decorrelation (or non-correlation) and potential biases between biomechanical properties obtained by MR-elastography, DWI-elastography, and US-elastography (extreme elasticity values, patient morphotype, etc.).   6. Association Between Biomechanical Properties and Histological Type of Lesion:  - Analyze associations between biomechanical properties predicted by the CT scan model, MR-elastography, DWI-elastography, and US-elastography with the final histological type of the lesion (for RCC: associations with histological grade),  - Perform associations between categorical histological variables and numerical elasticity values – compare ROC curve areas, and identify cut-off values where applicable.  7. Verify the Correlation Between Real Hardness and Hardness Evaluated by Different Imaging Methods:   - Perform direct evaluation on fresh operative specimens to verify the correlation between actual hardness and the hardness assessed by the different imaging methods. |
| --- | --- |
| **Criteres de jugement** | **Critère de jugement principal :**  Le critère de jugement principal est la racine de l’erreur quadratique moyenne (ou « root mean square error », RMSE – sans unité). L’objectif est de réussir à prédire **μMRE** à partir de **dCT-, dCT40s, dCT90s, dCT10min** (en nommant μCT la prédiction du modèle scanographique) avec l’erreur la plus faible possible.  **Critères de jugement secondaires :**   1. Le critère de jugement sera le rho de Spearman, avec pour objectif qu’il soit le plus élevé possible (maximum = 1). 2. L’évaluation de la qualité sera basée sur une échelle qualitative ordinale en 5 points. Le contraste sera calculé grâce au ratio contraste sur bruit. Le bruit sera évalué par le calcul du ratio signal sur bruit. La présence d’artefacts sera annotée pour chaque examen. 3. Identification des facteurs limitants (morphotype patient [body mass index, BMI], sarcopénie, localisation, taille et architecture de la lésion) |

|  | counting situations where the examination has no diagnostic clinical value and associations with potential limiting factors)  4. The outcome measures will be the intra-class correlation coefficients and the Bland-Altman plot traces. The goal is to achieve the highest possible intra-class score (maximum = 1, ideally >0.90).  5. Situations of decoupling between hardness measurements obtained from different imaging modalities will be visually identified by plotting scatterplots for each patient and anatomical situation where matching is possible, with hardness values on the X-axis from one imaging modality and hardness values on the Y-axis from another modality. Descriptive characteristics of the points (voxel or patient segmentation) with decoupling will then be analyzed.  6. The outcome measures will rely on associations between categorical histological variables and numerical elasticity values, with comparisons of areas under the ROC curve or the identification of cut-offs if applicable. The malignant/benign nature (binary variable) and histological type (non-ordinal categorical variable) will be evaluated in the current clinical care framework by the senior expert urological pathologist at the CHU.  7. This evaluation will be conducted directly on a fragment of the fresh surgical specimen. The real hardness in kPa will be assessed by the physicist immediately post-operatively on the bench using a destructive method on a fragment of the specimen, with the remaining part being sent for histological analysis. |
| --- | --- |
| **Schema de la recherche** | Exploratory Diagnostic Evaluation Study, Monocentric, Prospective, Based on Imaging Exams |
| **Criteres d’inclusion** | - Age ≥ 18 ans, - Prise en charge chirurgicale programmée pour néphrectomie pour tumeur du rein dans le département d’urologie du CHU de Bordeaux, - Uro-scanner disponible ou programmé pour la chirurgie, - Consentement exprimé pour intégration de la cohorte UroCCR, - Consentement exprimé pour participation à l’étude Rein-3D Print Mechanics, - Personne affiliée ou bénéficiaire d’un régime de sécurité sociale |
| **Criteres de non inclusion** | - Pregnant or breastfeeding women  - Contraindication to MRI  - Presence of an electronic medical device contraindicating MRI (pacemaker, defibrillator, cochlear implants, neurostimulator), stent less than 6 months old, incompatible heart valve, presence of intraocular metallic foreign bodies, ongoing pregnancy  - Contraindication to the injection of gadolinium contrast agents  - Potential biopsy performed before scheduled MRI, CT scans, and ultrasound (which could alter the mechanical properties of the renal tumor and renal parenchyma due to iatrogenic hemorrhagic changes)  - Presence of thoracolumbar arthrodesis material  - Obese patient (body mass index ≥ 30 kg/m²)  - Cystic renal tumors with solid components (either wall thickening or tumor budding) < 2 cm  - Necrotic renal tumors with solid components (either wall thickening or tumor budding) < 2 cm  - Ascites  - Person under legal protection measures (safeguard of justice, guardianship, or curatorship)  - Difficulties in understanding and expressing oneself in French |

|  |  |
| --- | --- |
| **Strategie/ procedures de la recherche** | In all patients: renal MRI with DWI-elastography and MR-elastography sequences according to standardized acquisition and interpretation protocols, coupled with ultrasound with US-elastography → within 28 days following the pre-operative planning CT scan and prior to surgery or any other invasive diagnostic procedure (biopsy, ablation, embolization...).  Anatomopathological analysis of the excised specimen coupled with a measurement of the physical hardness of the tissue upon receipt in its fresh state. |
| **Taille d’etude** | 50 patients |
| **Duree de la recherche** | Durée de la période d’inclusion : 18 mois  Durée de participation de chaque participant : entre 15 jours et 4 mois Durée totale de la recherche : 22 mois |
| **Statistical Analysis of the data** | At the end of the imaging acquisitions and a co-registration step, we will obtain the following variables for each region of interest (ROI, manually traced – i.e., ROI analysis), each anatomical region of the kidney (cortex, medulla, tumor – i.e., regional analysis), and each voxel (i.e., voxel analysis):   - MR-elastography: μMRE - DWI-elastography: ADCb200, ADCb1500, μDWI - US-elastography: μUS (only in the ROI analysis) - Scanner: dCT-, dCT40sec, dCT90sec, dCT10min   We will also collect a set of potential confounding variables related to the patient (age, sex, BMI, sarcopenia, glomerular filtration rate [GFR], height of each kidney, thickness of the cortex of each kidney, skin-to-measurement distance) and related to the lesion (size, volume, anterior/posterior location, superior/equatorial/inferior pole, endophytic/exophytic).  Additionally, the final diagnosis of the lesions will be recorded: benign / malignant, histological type, and for clear cell renal carcinoma (CCCR), the histological grade.  All these variables will be described in terms of absolute count and percentages for categorical variables and in terms of mean, standard deviation, median, minimum, maximum, and interquartile range for numerical variables.  **Regarding the primary objective** of developing a predictive model for y = μMRE according to the variables X = dCT-, dCT40sec, dCT90sec, dCT10min (known as the scanographic model):  We will have several observations per patient, of different natures (tumor, healthy parenchyma), obtained through various methods (voxels, ROIs, anatomical regions). The sample will be split into a training set and a test set (70%-30% of the population). Several regression algorithms will be trained using repeated nested cross-validation (linear regression, penalized linear regression elasticnet, k-nearest neighbors, support vector machine, random forest, artificial neural network, etc.) and selected based on RMSE. The performance of the best models will then be independently evaluated on the test sample.  **Regarding the secondary objectives**:   - The correlations for each pair of imaging variables across all ROIs will be evaluated using the Spearman rank test. - The relationships between μDWI, μMRE, and μUS and the scanographic densities at different time points will be empirically explored (linear, logarithmic, quadratic, etc.). - The repeatability of the elasticity measurements will be evaluated using the Bland-Altman method and the intra-class correlation coefficient (ICC).   **Associations between the predictions of the scanographic model**, μDWI, μMRE, and μUS, and potentially confounding patient and lesion variables will be studied. Additionally, associations between the predictions of the scanographic model, μDWI, μMRE, and μUS, and clinically relevant characteristics of the renal lesions will be explored using:  - Spearman tests (for pairs of numerical variables),  - Mann-Whitney tests (for pairs of categorical and numerical variables),  - Chi-square and Fisher's tests(for pairs of categorical variables).  **Feasibility of actual tissue hardness measurements** of tumor tissue in the immediate postoperative phase (i.e., reliable measurements that do not damage the tissue) will be assessed. If feasible, **correlations** between the real tissue hardness measurements and those obtained through imaging will be explored. |

|  |  |
| --- | --- |
| **Retombees attendues** | - Predictive model of renal elasticity via MRI based on scanner data.  - Validation of a diffusion-elastography sequence at 1.5 Tesla.  - Better understanding of the physiological, pathological, and artifact-related factors influencing the elasticity of healthy and tumoral renal tissue.  - Identification of novel biomarkers for the malignant nature of renal lesions, histological type, and potentially histological grade.  - Potential patent for the predictive scanographic model.  - The results of this study could provide new diagnostic insights to improve the characterization of renal lesions and the realism of 3D printed models used for therapeutic education and training purposes. |

# ABSTRACT

This research has been registered in <http://www.clinicaltrials.gov/> under the n° NCT06525831

# Multimodal imaging of the biomechanical properties of kidney tumors: feasibility, inter-modality correspondence and diagnostic value (UroCCR-115). R3DP-M

The University Hospital of Bordeaux is the sponsor of this research.

This research will be conducted with the support of ANR-21-RHUS-0015.

### Brief summary:

The goal of this innovative project is to evaluate the correspondence between several imaging modalities for characterizing the elasticity of healthy and pathological renal tissue which could help improve the realism of 3D prints used by urological surgeons and allow the identification of new, complementary imaging biomarkers. The main objective is to develop a predictive model of the biomechanical properties of normal and pathological kidney tissue, as assessed by the reference method (MR-elastography).

### Detailed description :

Medical imaging plays a key role in the diagnostic and therapeutic management of renal cell carcinomas. It can be used to confirm the presence of a tumor, localize it, suggest malignancy or even histological subtype, guide sampling, perform TNM staging, assist in surgical scheduling, monitor therapeutic efficacy in the event of systemic treatment, guide ablathermy procedures and look for relapses after curative treatments have ended. Imaging of kidney tumors relies on three complementary imaging modalities: ultrasound (US), CT based on X-ray absorption (CT-scan) and magnetic resonance imaging (MRI). The most commonly performed examination remains the CT scan, which is used to print 3D models. However, the correlation between renal parenchyma densities and renal tumors (before and during the scan acquisition times after injection) and elasticity parameters measured by US and MRI has never been explored.

This trial aim to evaluate the correspondence between several imaging modalities for characterizing the elasticity of healthy and pathological kidney tissue. It will also improve the realism of 3D models used by surgeons, and identify new complementary imaging biomarkers.

To achieve this aim, 50 patients will undergo DWI-elastography and MR-elastography sequences, as well as an US before the surgery. After surgery, a fragment of the resected specimen will be used to perform mechanical tests to determine the real hardness of the tissue.

### Primary outcome:

Measurement of the root mean square error (RMSE). The aim is to successfully predict μMRE from dCT-, dCT40s, dCT90s, dCT10min (naming μCT the scan model prediction) with the lowest possible error.

### Secondary outcomes:

1. Measure of Spearman's rho, aiming for the highest possible value (maximum = 1).
2. Measure of the qualitative assessment based on a 5-point ordinal qualitative scale. Calculation of contrast using the contrast-to-noise ratio. Evaluation of noise using the signal-to-noise ratio. The presence of artifacts will be annotated for each examination.
3. Identification of limiting factors (patient morphotype [body mass index, BMI], sarcopenia, lesion location, size and architecture) (count situations where the examination has no clinical diagnostic value, and associate with potential limiting factors).
4. Evaluation of the repeatability of MR-elastography, DWI-elastography and US-elastography on renal parenchyma and renal tumors: on the whole sample, according to patient morphotype, lesion location, size and architecture (calculation of intra-class correlation coefficients and Bland-Altmann plot traces).
5. Identification of situations of de-correlation (or non-correlation) and potential bias between biomechanical properties obtained by MR-elastography, DWI-elastography and US-elastography (extreme elasticity values, patient morphotype, ...).
6. Carry out associations between biomechanical properties obtained by CT model predictions, MR- elastography, DWI-elastography, US-elastography and final histological type of lesion (if CCCR: associations with histological grade) (associations between histological categorical variables and numerical elasticity variables - comparison of area under the ROC curve, identification of cut-offs where appropriate).
7. Verification of correlation between actual hardness and hardness assessed by the various imaging methods. This assessment is carried out directly on the fresh surgical specimen.
   - **Study design :** Exploratory, monocentric, prospective, imaging-based diagnostic evaluation study

### Eligibility criteria:

- - - Inclusion criteria:
  - Adult patients (≥ 18 years of age)
  - Scheduled for surgical management with nephrectomy for kidney tumor in the urology department of Bordeaux University Hospital
  - CT scan available or scheduled for surgery
  - Consent expressed for integration of the UroCCR database
  - Expressed consent for participation in the Rein 3D Print Mechanics study
  - Patients affiliated or benefiting from social security system
    - Exclusion criteria:
  - Pregnant or breast-feeding women
  - Contraindication to MRI
  - Contraindication to injection of gadoline contrast agents
  - Biopsy prior to MRI, CT and ultrasound scans (risk of artifactual alteration, via iatrogenic hemorrhagic changes, of the biomechanical properties of the renal tumor and parenchyma).
  - Presence of thoracolumbar arthrodesis material
  - Obese patient (body mass index ≥ 30 kg/m²)
  - Cystic renal tumors with solid component (corresponding to either parietal thickening or tumor bud)

< 2 cm

- - Necrotic renal tumors with solid component (corresponding to either parietal thickening or tumor bud) < 2 cm
  - Ascites
  - Person under legal protection
  - Difficulty understanding and expressing in French

*.*

- **Interventions** : We will add a MR-elastography and Ultrasound exams for the research. The exams will be performed between the inclusion of the patient and the day before the surgery.

### Number of subjects: 50

- **Statistical analysis:**

After imaging acquisitions and a co-registration step, we will obtain for each region of interest, each anatomical region of the kidney and each voxel, the following variables:

- - MR-elastography: μMRE
  - DWI-elastography: ADCb200, ADCb1500, μDWI
  - US-elastography: μUS (only in ROI analysis)
  - CT-Scan: dCT-, dCT40sec, dCT90sec, dCT10min

We will also have a set of possibly confounding variables related to the patient, the lesion and the final diagnosis of the lesions: benign/malignant, histological type, and for RCC the histological grade.

The main objective consists in developing a predictive model of y = μMRE according to the variables X = dCT-, dCT40sec, dCT90sec, dCT10min (so-called scanographic model): we will have several observations per patient, of different natures (tumor, healthy parenchyma), obtained by different means (voxels, ROI, anatomical regions).

The sample will be split into a training sample and a test sample (70%-30% of the population). Several regression algorithms will be trained in repeated nested cross-validation (linear regression, elasticnet penalized linear regression, k-nearest neighbors, support vector machine, random forest, artificial neural network...) and selected according to RMSE. The performance of the best models will then be independently evaluated on the test sample.

- **Condition:** Kidney cancer surgery
- **Key-words:** Renal-Cell Carcinoma, 3D Printing, Renal Elasticity, Biomarkers, Ultrasound, CT-scan, Magnetic Resonance Imaging, AFRI, Diffusion weighted imaging

# SCIENTIFIC JUSTIFICATION AND GENERAL DESCRIPTION

## CURRENT STATE OF KNOWLEDGE

- - 1. ON REFERENCE PROCEDURES UNDER STUDY

Renal carcinomas account for 3–5% of all adult malignant tumors, representing the 7th leading cause of cancer-related death in men and the 10th in women (1). More than 50% of renal carcinomas are discovered incidentally during imaging performed for another indication (2). According to the World Health Organization (WHO) classification, the main histological types are: (i) clear cell renal carcinomas, representing 80% of renal carcinomas; (ii) papillary carcinomas types 1 and 2 (with type 2 showing a more aggressive phenotypic presentation); and (iii) chromophobe carcinomas.

Medical imaging plays a central role in the diagnostic and therapeutic management of renal carcinomas. It allows for confirmation of tumor presence, localization, guidance toward malignancy or even histological subtype, biopsy guidance, TNM staging, surgical planning, monitoring of therapeutic efficacy in systemic treatment, guidance of ablative procedures, and detection of recurrences after completion of curative treatments.

Although these cancers exhibit certain radiological features that may suggest their diagnosis, particularly on multiparametric MRI (mpMRI), these radiophenotypic features remain insufficiently sensitive and specific for systematic precise diagnosis. Indeed, according to a recent meta-analysis including 1,239 solid renal lesions, the percentage of lesions correctly classified by MRI in terms of malignancy was 64%, with a sensitivity of 95% and specificity of 63% (3). In another prospective multicenter study including 250 tumors smaller than 4 cm, MRI sensitivity and specificity were 75% and 79%, respectively, for the diagnosis of clear cell carcinoma (CCRC), with modest inter-observer reproducibility (kappa = 0.58) (4).

As a result, 30% of partial and total nephrectomies are performed for benign tumors (notably, fat-poor angiomyolipomas and oncocytomas)—representing invasive procedures that could have been avoided, with significant local complications (hemorrhages, fistulas, infections – in 16% of cases) (2,5).

It is therefore essential to improve the characterization of renal tumors through imaging, particularly via complementary and independent information. Evaluation of the biomechanical properties of renal tumors—by ultrasound, MRI, or even CT—could enrich radiological analysis and enhance the realism of 3D models of healthy and pathological kidneys.

- - 1. Sur les procedures de reference et a l’etude

Three complementary imaging modalities are used to analyze healthy renal parenchyma, pathological tissue, and renal tumors: ultrasound, CT, and MRI.

The examination providing the most information for characterizing a renal tumor is MRI using so-called multiparametric protocols (mpMRI), including basic morphological sequences (T1, T2), sequences identifying microscopic fat (T1 in opposed-phase inversion), tumor vascularization (dynamic acquisitions after intravenous contrast injection), and sequences analyzing cellularity and tumor architecture (diffusion). However, as previously mentioned, mpMRI—even when assisted by machine-learning algorithms to better integrate extracted data into predictive models—cannot systematically ensure an accurate diagnosis (accuracy = 64% for malignancy diagnosis according to a meta-analysis of 1,239 patients (3), and 81–98% according to histological subtypes using AI in exploratory, clinically unvalidated studies (6–8)).

Furthermore, MRI using specifically parameterized diffusion sequences (DWI-E) and MR elastography sequences (MRE) can quantify tissue elasticity, with encouraging preliminary results on healthy and pathological kidneys (tumoral, chronic kidney disease, and fibrosis). Studies have shown that renal parenchyma stiffness is inversely correlated with the percentage of tubulointerstitial fibrosis, extracellular matrix volume, and glomerulosclerosis proportion in patients with chronic renal lesions (9,10). Other studies have shown an association between intra-tumoral elasticity values and certain histological types (11). These findings suggest the potential diagnostic value of quantifying the biomechanical properties of healthy and pathological kidneys.

However, the reference examination used to segment the different elements of renal anatomy (vein, artery, renal cortex and medulla, excretory tracts) to create 3D models for planning, educational, and informative purposes is CT, using multiphasic acquisition before and after contrast injection. To date, no correlation has been identified between CT and tissue elasticity. Being able to predict tissue elasticity values (using MRE values as reference) from multiphasic CT could improve the realism of 3D-printed models, which would incorporate voxel density data in addition to anatomical CT data, and potentially enhance predictive information (malignancy, histotype) provided by CT.

## RESEARCH HYPOTHESES AND EXPECTED RESULTS OF THE RESEARCH AND EXPECTED

In this project, we hypothesize that:

- There is a correlation between renal elasticity measured by MRI and ultrasound, though it may be imperfect and dependent on independent factors (e.g., patient morphometric factors).
- There is a relationship between densities obtained on multiphasic CT and elasticity values measured by MRE.

## BENEFIT/ RISK RATIO

### Benefits for patients :

The benefits for individual patients are limited, aside from providing assurance that they will receive a comprehensive and optimal preoperative imaging assessment. Biomechanical findings obtained during imaging examinations are unlikely to alter standard patient management. However, the elastography-MRI sequence performed as part of the protocol may provide additional information in terms of tumor characterization and future surgical planning.

There is also a potential future collective benefit. This project will improve understanding of the relationship between the biomechanical properties of kidneys and renal tumors across different imaging modalities. Ultimately, this project could:
(i) provide additional tumor characterization, potentially avoiding unnecessary biopsies or nephrectomies, and
(ii) create more accurate 3D models for urologic surgeons to enhance patient information and improve surgical preparation and training.

### Expected risks :

Patient care (urology consultations, biopsy, or excision surgery) is entirely part of standard care, except for elastography ultrasound and elastography-MRI. While MRI currently plays a major role in the pretherapeutic assessment of renal lesions and is often performed in addition to CT for better characterization, this examination is not yet standard care.

These examinations are neither painful, nor irradiating, nor invasive. Performing an elastography-MRI with contrast injection is considered safe if contraindications are respected. Elastography-MRI is noisy, but ear protection is provided. The examination also requires a lumbar vibration device during the sequence, which has been reported as well tolerated in previous studies (12). Ultrasound is risk-free. These procedures are only burdensome in terms of time required (approximately 20 minutes for ultrasound, 30–40 minutes for MRI).

The overall risk/benefit ratio favors conducting the study. Potential indirect benefits are numerous in terms of scientific knowledge, implementation of innovative techniques at Bordeaux University Hospital, and familiarization of medical and paramedical teams with new imaging technologies. There is no risk to patients regarding clinical practices or potential treatment delays, as the additional ultrasound and MRI will be performed without delay.

The principal investigator must continuously monitor, evaluate, and document risks, ensuring they can be managed satisfactorily.

## RESULTS EXPECTED

The expected outcomes are:

- **Scientific perspective:** improvement of knowledge on imaging of the biomechanical properties of healthy and pathological kidneys (previous studies on MRE included fewer than 50 patients in total, none specifically studied the DWI-E technique, none compared all imaging methods for the kidney, and none examined relationships with CT densities). We anticipate at least one scientific publication in imaging, one in urology on applications of these quantifications for 3D models, and related oral presentations (French Radiology Days, European Congress of Radiology).
- **Educational perspective:** this project provides an opportunity to implement innovative renal imaging techniques in routine clinical practice and to familiarize medical and paramedical teams with their use.
- **Collaborative perspective:** this project allows interaction between medical and scientific teams and industrial partners around a common theme, potentially strengthening a medico-scientific community on shared topics.
- **Clinical perspective:** if the results are positive, this study will provide new diagnostic arguments to improve the characterization of renal lesions and the realism of 3D-printed models for educational and training purposes.

# RESEARCH OBJECTIVES

## PRIMARY OBJECTIVE

The primary objective is to develop a predictive model of the biomechanical properties of normal and pathological renal tissue, evaluated by the reference method (MRE), based on densities obtained from the various phases of CT. Predictive models will be evaluated using multiple indices (R², MSE, RMSE). The best models will be selected based on RMSE.This evaluation is performed before the definitive histological diagnosis.

## SECONDARY OBJECTIVES

The secondary objectives are:

1. Correlate voxel-to-voxel, ROI-to-ROI, and anatomical region-to-region the elasticity of renal tumors and surrounding healthy renal tissue obtained by MR elastography, DWI elastography, and US elastography, with CT density values from different uro-CT phases.
2. Study the feasibility and parameterization of DWI elastography on a clinical 1.5 Tesla MRI:
   - evaluation of quality (5-point ordinal qualitative scale),
   - evaluation of contrast (contrast-to-noise ratio calculation),
   - evaluation of noise (signal-to-noise ratio calculation),
   - annotation of artifacts encountered.
3. Study the feasibility of MR elastography, DWI elastography, and US elastography to measure the elasticity of renal parenchyma and renal tumors; identify limiting factors (patient morphotype [BMI], sarcopenia, lesion location, size, and architecture) by recording cases where the exam has no diagnostic clinical value and associating them with potential limiting factors.
4. Evaluate the repeatability of MR elastography, DWI elastography, and US elastography on renal parenchyma and tumors: across the sample, according to patient morphotype, lesion location, size, and architecture (calculating intra-class correlation coefficients and Bland-Altman plots).
5. Identify situations of decorrelation (or non-correlation) and potential biases between biomechanical properties obtained by MR elastography, DWI elastography, and US elastography (extreme elasticity values, patient morphotype, etc.).
6. Perform associations between biomechanical properties obtained by CT model predictions, MR elastography, DWI elastography, US elastography, and the final histological type of the lesion (if CCRC: associations with histological grade). Associations will involve categorical histological variables and numerical elasticity values—comparing ROC areas and identifying cut-offs where applicable.
7. Verify the correlation between actual hardness and hardness assessed by the various imaging methods. This evaluation is performed directly on fresh surgical specimens.

# OUTCOME MEASURES

## PRIMARY OUTCOME MEASURE

The primary objective is to develop a CT-based predictive model to estimate the hardness of healthy and pathological renal tissues according to MRE measurements. Therefore, this is a regression problem.

The primary outcome measure is the root mean square error (RMSE – unitless). The goal is to predict μMRE from dCT-, dCT40s, dCT90s, dCT10min (with μCT representing the model’s predicted value) with the smallest possible error. Assuming ‘n’ observations (voxels, ROIs, regions), the model aims to minimize:


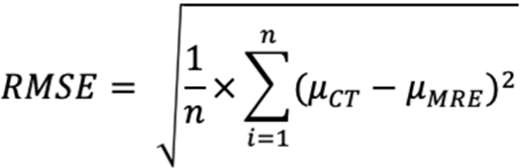


μMRE will be measured during the protocol MRI using the MRE sequence at 1.5 T in kPa (Philips Achieva dStream, Saint André Hospital). Senior radiologists from the uro-vascular imaging department at CHU Bordeaux will be responsible for data collection.

Each measurement is performed twice in the same session to assess sequence reproducibility. The sequence qualitatively deemed best by the radiologists will be used for regression algorithm development. There is no differential treatment, so no blinding is required.

μMRE will be measured during the protocol MRI using the MRE sequence at 1.5 T in kPa (Philips Achieva dStream, Saint André Hospital).
The data collection will be performed by senior radiologists from the uro-vascular imaging department at CHU Bordeaux.
Each measurement is performed twice in the same session to assess the reproducibility of the sequence. The sequence judged qualitatively best by the radiologists will be used for the development of the regression algorithm.
There is no differential treatment, so blinding is not required.

## SECONDARY JUDGMENT CRITERIA :

The secondary endpoints are defined to address the different secondary objectives:

1. The endpoint will be Spearman’s rho, with the aim of achieving the highest possible value (maximum = 1).
2. The quality assessment will be based on a 5-point ordinal qualitative scale. Contrast will be calculated using the contrast-to-noise ratio, and noise will be evaluated by calculating the signal-to-noise ratio. The presence of artifacts will be annotated for each examination.
3. Identification of limiting factors (patient morphotype [body mass index, BMI], sarcopenia, lesion location, size, and architecture) (counting situations where the examination has no diagnostic clinical value and associations with potential limiting factors).
4. The endpoints will include intraclass correlation coefficients and Bland–Altman plots. The objective is for the intraclass correlation score to be as high as possible (maximum = 1, ideally >0.90).
5. Situations of decorrelation between tissue stiffness measurements obtained by the different imaging modalities will be visually identified by plotting, for each patient and anatomical situation where matching is possible, scatter plots with X representing stiffness measured by one imaging modality and Y representing stiffness measured by another modality.

stiffness measured by another imaging modality. The descriptive characteristics of the points (voxel or patient segmentation) showing decorrelation will then be analyzed.

1. The endpoints will be based on associations between histological categorical variables and numerical elasticity values, using comparisons of areas under the ROC curve or identification of cut-offs when appropriate. Malignant/benign status (binary variable) and histological type (non-ordinal categorical variable) will be assessed as part of routine care by the senior urology pathologist at the CHU.
2. This assessment is performed directly on a fragment of the fresh surgical specimen. The actual tissue stiffness in kPa will be measured by the physicist immediately postoperatively at the bench using a destructive method on a fragment of the specimen, with the remainder sent for histological analysis.

# STUDY DESIGN

## JUSTIFICATION OF METHODOLOGICAL CHOICES

This is a prospective exploratory study of multimodal imaging of the biomechanical properties of renal tumors. Patients with renal tumors who meet the eligibility criteria will participate in the study, and preoperative imaging modalities specific to the research (MR elastography and ultrasound elastography) will be used in addition to routine modalities (CT scan and multiparametric MRI). Furthermore, the actual biomechanical properties of the surgical specimens from these patients will be measured.

The study follows a cross-sectional design, with a preoperative window allowed for the different imaging modalities.

The sample size (50 patients) is compatible with the exploratory nature of the study. The units of analysis for imaging will be the voxel (MRI and CT) and the ROI (ultrasound).

## STUDY SCHEME

Prospective single-center exploratory study


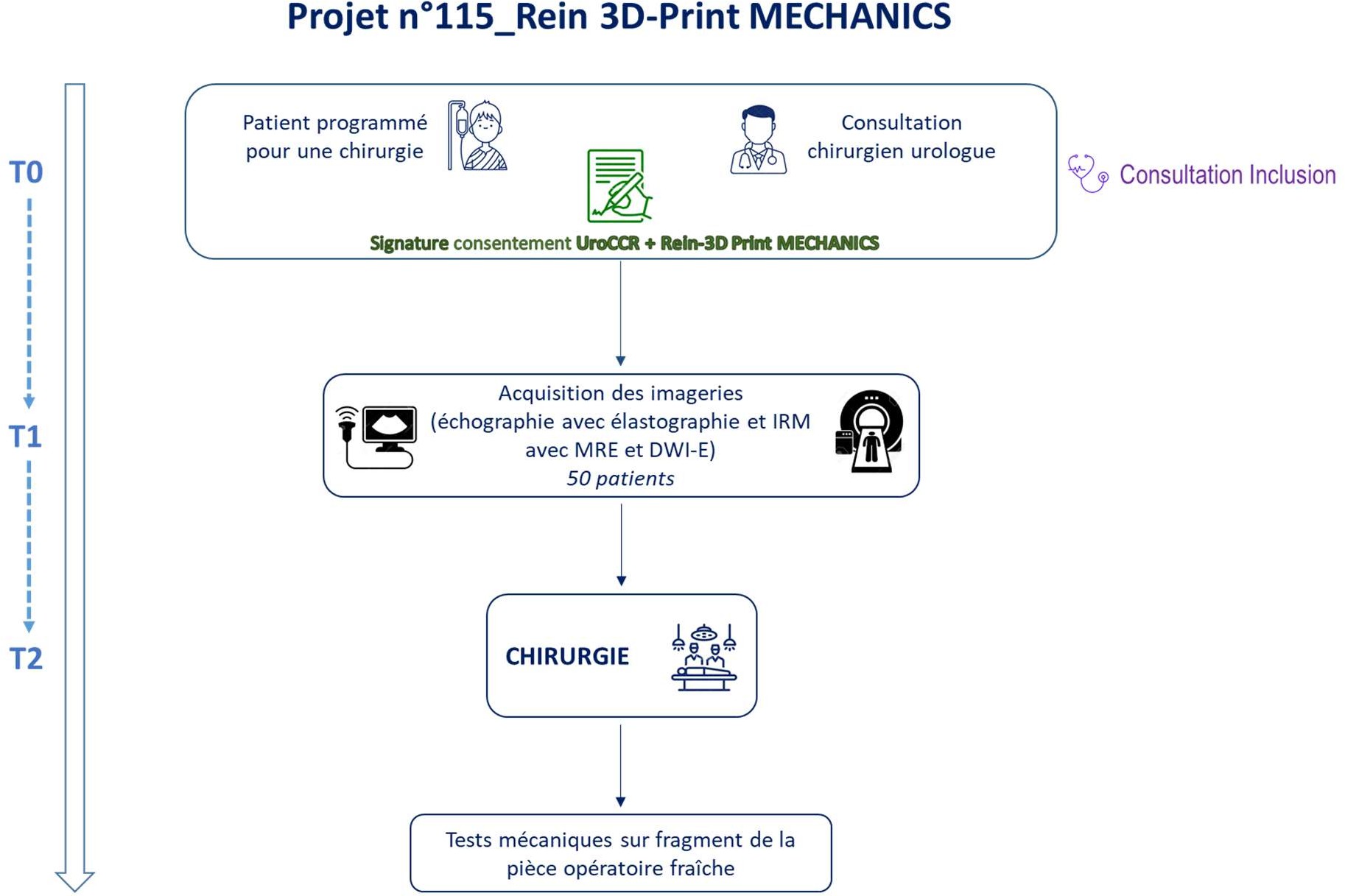


# ELEGIBILITY CRITERIA

## INCLUSION CRITERIA

- Age ≥ 18 ans,
- Scheduled surgical management for nephrectomy due to a renal tumor in the Urology Department of CHU de Bordeaux,
- Preoperative CT urography available or planned,
- Written consent for inclusion in the UroCCR cohort,
- Written consent for participation in the Rein-3D Print Mechanics study,
- Affiliation or coverage under a social security system.

## CRITERES DE NON INCLUSION

- Pregnant or breastfeeding women,
- Contraindication to MRI:
- Presence of electronic medical devices incompatible with MRI (pacemaker, defibrillator, cochlear implants, neurostimulator), stent <6 months old, incompatible cardiac valve, intraocular metallic foreign bodies, ongoing pregnancy,
- Contraindication to gadolinium-based contrast agents,
- Performance of a biopsy prior to protocol MRIs, CT scans, or ultrasound (risk of artifact alteration due to iatrogenic hemorrhagic changes affecting biomechanical properties of renal tumor and parenchyma),
- Presence of thoracolumbar arthrodesis hardware,
- Obesity (body mass index ≥ 30 kg/m²),
- Cystic renal tumors with solid component (wall thickening or nodular component) < 2 cm,
- Necrotic renal tumors with solid component < 2 cm,
- Ascites,
- Individuals under legal protection measures (guardianship, conservatorship),
- Difficulty understanding or communicating in French
- Pregnant or breastfeeding women,
- Contraindication to MRI:
- Presence of an electronic medical device incompatible with MRI (pacemaker, defibrillator, cochlear implants, neurostimulator), stent < 6 months old, incompatible cardiac valve, intraocular metallic foreign body, ongoing pregnancy,
- Contraindication to gadolinium-based contrast injection,
- Performance of a biopsy prior to protocol MRIs, CT scans, or ultrasound (risk of artifact alteration due to iatrogenic hemorrhagic changes affecting the biomechanical properties of the renal tumor and renal parenchyma),
- Presence of thoracolumbar arthrodesis hardware,
- Obese patients (body mass index ≥ 30 kg/m²),
- Cystic renal tumors with a solid component (wall thickening or nodular component) < 2 cm,
- Necrotic renal tumors with a solid component (wall thickening or nodular component) < 2 cm,
- Ascites,
- Individuals under legal protection measures (guardianship, conservatorship, or legal safeguard),
- Difficulty understanding or communicating in French.

## FEASIBILITY AND RECRUITMENT PROCEDURES

Approximately 250 patients per year are referred to the Urology and Radiology Departments of CHU de Bordeaux for initial evaluation of renal lesions.
These departments regularly collaborate in both clinical and research activities and participate in the same Multidisciplinary Team Meetings (RCP), where diagnostic and therapeutic management of renal tumors is discussed.

Patients will be recruited by urologists of the Pellegrin Hospital Urology Department during consultations with external patients referred for renal tumors meeting inclusion criteria, or following referral by radiologists of the Adult Radiology Department at Pellegrin Hospital during imaging sessions if a patient meeting inclusion criteria is identified.

Thus, recruiting 50 patients over an 18-month period, with an additional 4-month follow-up period, appears feasible.

# RESEARCH PROCEDURES

## EXPERIMENTAL PROCEDURES

### ULTRASOUND ELASTOGRAPHY USING THE ARFI METHOD

Ultrasound elastography using the ARFI method (Acoustic Radiation Force Impulse, available on Siemens clinical devices with ARFI module at CHU) uses short pulses (0.03–0.4 ms) to excite tissues and generate a shear wave. This wave is analyzed in a sampling volume to infer a focal measurement of shear wave velocity and, consequently, tissue elasticity.

This excitation region corresponds to a rectangular ROI of 1×0.5 cm in ultrasound, which can be freely positioned within the tissues up to a maximum depth of 8 cm from the skin surface. The measurement is proportional to tissue elasticity and is expressed in meters per second, corresponding to the speed of the shear wave perpendicular to the source impulse.

In practice, patients are positioned in lateral decubitus during the ultrasound. The ROI is placed within the tumor under visual guidance using the B-mode. Ten measurements are performed, and the median value is used as a reference.
The additional duration of the examination is less than 5 minutes. It is completely painless, non-invasive, and carries no contraindications.

### MR Elastography Using DWI-E and MRE Methods

MRIs will be performed at CHU de Bordeaux on a 1.5 T Philips Achieva dStream (Saint André Hospital). The system is equipped with the Resoundant® module (Resoundant Inc., Mayo Clinic).

For the DWI-E method, we will apply the technique proposed by Le Bihan et al. in Radiology (2017), based on an empirical formula linking tissue elasticity to apparent diffusion coefficient (ADC) values obtained with diffusion sequences at b = 200 s/mm² and b = 1500 s/mm², respectively. No gadolinium-based contrast injection is required for this sequence.

The MRE sequence allows the study of tissue mechanical properties in three steps:

1. An external membrane generates a mechanical wave in the body.
2. An MRI sequence captures images of wave propagation in the tissues.
3. An algorithm analyzes wave propagation to generate tissue elasticity maps.

The resulting measurement, proportional to tissue elasticity, is expressed in kPa.

In practice, during standard abdominal MRI, patients are placed in the supine position with a 19 cm passive membrane positioned under the back against the scanner table. The abdominal coil is positioned above the patient. Continuous acoustic vibrations are transmitted from the device in the technical room to the passive membrane via an air tube and then to the kidney.

A 2D gradient-echo sequence collects images of shear waves in the renal parenchyma. Four slices are acquired using four consecutive breath-holds, predefined and centered on the renal tumor under study.
The time required to install the membrane and acquire images is approximately 5–10 minutes, in addition to the standard MRI exam (~30 minutes).

Contraindications are those of MRI. There are no additional restrictions for these specific sequences, which are fully non-invasive and painless for the patient.

## Comparison Procedure

• Scheduling of a preoperative planning CT urography is an inclusion requirement. Data from this CT will serve as input for the CT-based predictive model.

• The planning CT urography is performed as part of routine care at CHU de Bordeaux, in the Imaging Department of Pellegrin Hospital. It includes five acquisitions, four of which will be used for texture analysis (non-contrast phase, arterial phase at 40 s, nephrographic phase at 90 s, and delayed phase at 10 min post-injection of 20 mg furosemide as part of standard care). A helical acquisition is solely for preoperative purposes: the early arterial phase is automatically triggered upon detection of iodinated contrast arrival in the aorta.

• Multiparametric MRI (mpMRI) is now regularly performed in addition to CT to better characterize renal lesions. Approximately 450 mpMRIs are performed annually at CHU de Bordeaux, representing one-third of urological MRI activity. mpMRI is performed according to a standardized protocol consistent with published literature, for which the Uro-Vascular Imaging Department of CHU de Bordeaux is actively involved.

• Renal tumors in included patients will be surgically treated as part of routine care according to the approach deemed most appropriate by the urological RCP based on all available data. This procedure is not part of the study evaluation (it would occur as part of standard care) but will provide the final histopathological diagnosis, which is a secondary surgerical outcome .This is the early arterial phase acquisition, automatically triggered by detection of the arrival of iodinated contrast in the aorta.

• Multiparametric MRI (mpMRI) is now routinely performed in addition to CT scans to better characterize renal lesions. This examination accounts for approximately 450 MRIs per year at CHU de Bordeaux, representing about one-third of MRI activity for urological indications. The mpMRI is performed according to a standardized protocol consistent with published studies, which is a research focus of the Uro-vascular Imaging Department at CHU de Bordeaux (14–16).

• The renal tumors of included patients will be surgically treated as part of routine care, following the most appropriate approach as decided by the urological multidisciplinary team (RCP) based on all available data. This procedure is not part of the practices being evaluated in the project (it would in any case be performed as standard care), but it will provide the final histopathological diagnosis, which is one of the secondary endpoints.

## POST-PROCESSING OF ACQUIRED IMAGES

La **Figure 1** illustrates the types of imaging achievable in the study population.


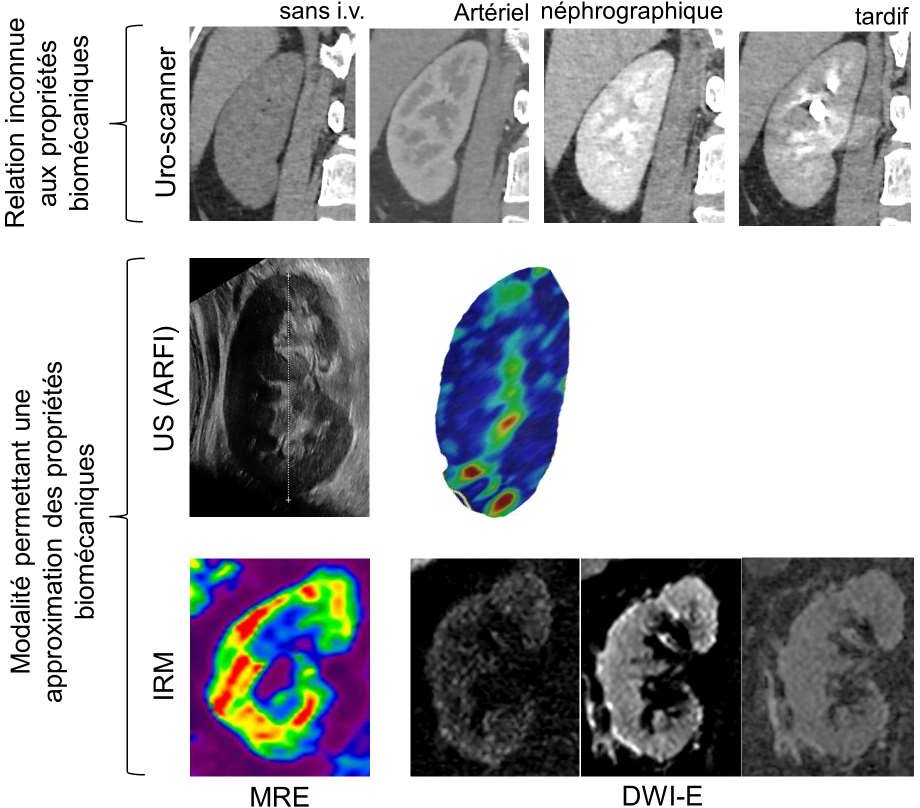


**Figure 1.** Différentes modalités d’imagerie réalisables sur le rein (exemple d’un rein sain)

The tissue elasticity maps obtained by DWI-E and MRE, along with the phases of the CT urography, will all be co-registered to achieve accurate voxel-to-voxel alignment.
The locations of the elasticity measurements by ultrasound will be standardized.
Observations $x$ at the same point in space for CT densities and elasticity measurements by DWI-E and MRE may correspond to:

- either voxels of the same size (after standardizing voxel sizes and interpolating voxel values),
- or anatomical regions (segmented automatically using deep learning or manually),
- or regions of interest manually drawn according to a standardized protocol (as in the case of ultrasound).

We will explore these three methods to obtain the reference elasticity values from MRE (the variable to be predicted) and the model input values (densities at different phases, i.e., predictors), which will be used to

train different machine learning algorithms dedicated to regression in order to ultimately obtain the most performant CT-based predictive model.

The **figure 2** illustrates the overall study pipeline.


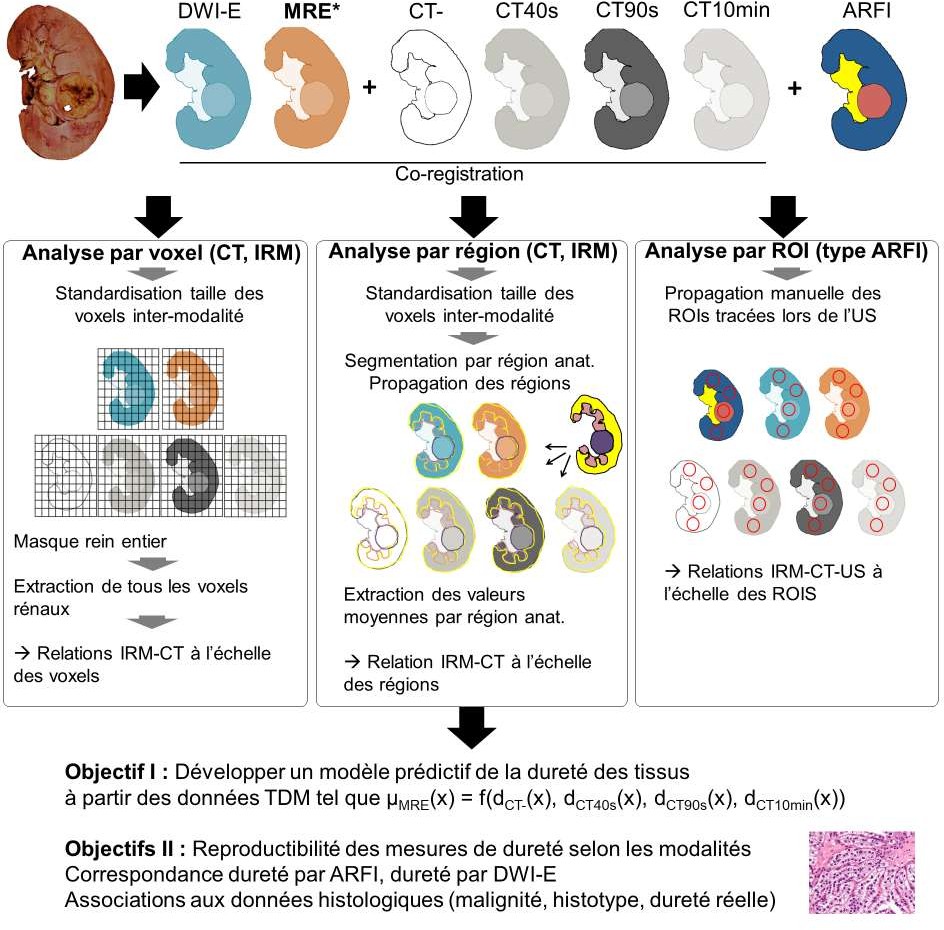


**Figure 2**. Overall schematic of the analysis and data acquisition methods for the development of the CT-based predictive model of the stiffness of healthy and pathological renal tissues

# CONDUCT OF THE STUDY

## STUDY TIMELINE

## - Duration of the inclusion period: 18 months

## - Maximum participation duration for each participant: between 15 days and 4 months

## - Total duration of the study: 22 months

## PARTECIPANT FOLLOW- UP SUMEMRY TABLE

|  | Inclusion | Images Acquisition | Surgery(S) |
| --- | --- | --- | --- |
|  | T0 | T1  Between T0 and the day before surgery at the latest | T2  Between T0 et T0 + 4 months |
| Eligibility verification (R) | ✓ |  |  |
| Information and collection of informed consent (R) | - 3 |  |  |
| Contrast-enganced CT urograohy available or scheduled before surgery S) ^1^ | - 2 |  |  |
| Ultrasound with elastography (ARFI) (R) |  | - 2 |  |
| MRI with MRE and DWI-E (R) |  | - 2 |  |
| Mechanical testing on fresh surgical specimen (R) |  |  |  |
| Collection of adverse events/serious adverse events (R) |  |  |  |

1. *If the standard-of-care CT urography is not available at the T0 visit, it will be scheduled between the inclusion visit and the day before surgery.*
2. *The CT urography, ultrasound, and MRI examinations should ideally be scheduled to maintain a 15-day interval between the CT and MRI (maximum 28 days).*
3. *The informed consent can be obtained between the inclusion consultation and the first research imaging examination (MRI or ultrasound).*

***R:****examination performed specifically as part of the research****S:****examination performed as part of routine clinical care*

## T0 : INCLUSION VISIT

- - 1. Collection OF consent

During the inclusion visit, the investigator informs the participant and answers all questions regarding the objective, nature of constraints, foreseeable risks, and expected benefits of the study. The investigator also explains the participant’s rights in the context of research and verifies eligibility criteria.
A copy of each information note (REIN 3D-PRINT MECHANICS UroCCR No. 115 and UroCCR) is provided to the participant by the investigator. After this information session, the participant is given time to reflect.
If the participant agrees to participate, both the participant and the investigator print their names, date, and sign two original copies of the consent forms. These must be signed **BEFORE ANY CLINICAL OR PARACLINICAL EXAMINATION REQUIRED BY THE RESEARCH**

The copies of the information notes and consent forms are distributed as follows:

- One original copy of each information note and signed consent form is given to the participant.
- The other original copies are retained by the investigator (even if the participant moves during the study) in a secure location inaccessible to third parties.
  - 1. CONDUCT OF THE visit

The inclusion visit is conducted by the investigator, who verifies all eligibility criteria. Before any research-related examination, the investigator obtains the participant’s free and informed consent (or that of their legal representative, if applicable).
A urine pregnancy test will be performed for women of childbearing age. If the test is positive, the patient will be excluded.
As part of routine care, the patient will have a contrast-enhanced CT urography performed before surgery if not available at the inclusion visit.
Once the participant agrees to participate, they will receive information about the organization and conduct of the ultrasound and MRI examinations performed as part of the study.

## T1 : IMAGE ACQUISITION

Examinations will be performed between inclusion and surgery by senior radiologists from the uro-vascular imaging department, always using the same ultrasound device.

- **MRI Elastography**
  The MRI will be performed on a different day than the CT scan, as is standard practice, due to the administration of contrast agents for both CT and MRI. It will be conducted in the Saint-André imaging department and will last 30–40 minutes.
  The contrast agents used in the study are those routinely used at CHU Bordeaux: DOTAREM® and CLARISCAN®. The dosage follows the marketing authorization recommendation: 0.1 mmol/kg for injection of DOTAREM or CLARISCAN 0.5 mmol/mL.
  The CT and MRI should ideally be scheduled to maintain a 15-day interval between the scans in case the tumor evolves (e.g., development of necrosis). The maximum interval should not exceed 28 days.
- **Ultrasound**
  Ultrasound will be performed at Pellegrin Hospital and can be combined with the planning CT urography. The examination will last approximately 15 minutes.

## MECHANICAL TESTS ON FRESH SURGICAL SPECIMEN

## The T2 visit corresponds to the scheduled surgery as part of routine care. Only the mechanical test performed on the surgical specimen is part of the research. Mechanical tests will be performed on the extracted specimen in the operating room by the surgeon. A biopsy punch will be used to obtain a sample of tumor tissue while preserving margins for standard histopathological analysis. This procedure has been validated by the reference pathologist at CHU Bordeaux (Dr. YACOUB). The sample will then undergo a compression test to measure tissue resistance. The tissue will be subjected to increasing compression until rupture. Data will be continuously recorded, analyzed, and compared to imaging data.

## VISITE DE SUIVI

No follow-up visit is planned as part of the study.

## VISITE DE FIN DE LA RECHERCHE

The end of participation in the study will occur upon collection of the results of the histopathological examination performed on the surgical specimen as part of standard care (date of the pathology report, up to 1 month after surgery).

There is no formal end-of-study visit; the patient is followed only for the duration necessary to complete the imaging examinations. The end-of-study point corresponds to the completion of the last examination (ultrasound or MRI) and, at the latest, the day before surgery. The patient is then followed within routine care for the management of their condition.

La fin de participation à l’étude se fera lors du recueil des résultats de l’examen anatomopathologique réalisé sur la pièce opératoire dans le cadre du soin (date du compte-rendu anatomopathologique, jusqu'à 1 mois après la chirurgie).

## REGLES D’ARRET

- - 1. Arret de la participation d’une personne a la recherche

Le participant qui souhaite abandonner ou retirer son consentement de participation à la recherche (comme il est en droit de le faire à tout moment) n’est plus suivi dans le cadre du protocole, mais doit faire l’objet de la meilleure prise en charge possible compte tenu de son état de santé et de l’état des connaissances du moment.

Un **abandon** est une décision d’un participant inclus de faire valoir son droit d’interrompre sa participation à une recherche, à tout moment au cours du suivi, sans qu'il n'encoure aucun préjudice de ce fait et sans avoir à se justifier.

Un **retrait de consentement** est une décision d’un participant de revenir sur sa décision de participer à une recherche et de faire valoir son droit d’annuler son consentement éclairé, à tout moment au cours du suivi et sans qu'il n'encoure aucun préjudice de ce fait et sans avoir à se justifier.

L’investigateur doit identifier la cause de l’abandon/du retrait de consentement et évalue s’il est possible de recueillir la variable sur laquelle porte le critère de jugement principal au moment de l’abandon/du retrait de consentement. Les abandons/retraits de consentement doivent être notifiés rapidement au centre investigateur coordonnateur, au promoteur et au centre de méthodologie et de gestion des données. Leurs raisons et leur date doivent être documentées dans le cahier d’observation et dans le dossier médical du participant.

- - 1. Arret de la recherche

**Fin de la recherche ou arrêt prévu de la recherche** : terme de la participation de la dernière personne qui se prête à la recherche aussi appelé dernière visite du dernier participant inclus dans la recherche.

Lorsque la recherche a atteint son terme prévu (arrêt prévu), la fin de la recherche doit être déclarée à l’ANSM et au CPP dans un délai de 90 jours.

**Arrêt anticipé de la recherche :** la recherche clinique est arrêtée (définitivement) de façon anticipée. C’est le cas, notamment, lorsque le promoteur décide :

- de ne pas commencer la recherche malgré l'obtention de l'autorisation de l'ANSM et de l'avis favorable d’un CPP ;
- de ne pas reprendre la recherche après l’avoir interrompu temporairement ou après sa suspension par l’ANSM.

Lorsque la recherche est arrêtée (définitivement) de façon anticipée, la fin de la recherche doit être déclarée au CPP et à l’ANSM dans un délai de 15 jours en indiquant les raisons qui motivent cet arrêt.

**Arrêt temporaire de la recherche :** l'arrêt temporaire d'une recherche clinique consiste en :

- l'arrêt de l'inclusion de nouvelles personnes dans cette recherche;
- et/ou l’arrêt de la pratique des actes prévus par le protocole de la recherche.

Toute décision du promoteur d'interrompre temporairement la recherche doit faire l'objet d'une information immédiate à l'ANSM et au CPP concerné et dans un second temps et dans un délai maximum de 15 jours calendaires suivant la date de cette interruption, d'une demande d’autorisation de modification substantielle concernant cet arrêt temporaire soumise à l'ANSM et d’une demande d’avis au CPP concerné.

## PROTOCOL DEVIATIONS

## Deviations can concern any aspect of the study: inclusion process, follow-up, endpoint measurement, treatments. All deviations must be documented and discussed in the Scientific Council. Only withdrawals lead to the cessation of follow-up. Even in case of protocol deviations, participant follow-up should continue as scheduled.

- - 1. PREMATURE AND DEFINITIVE TERMINATION OF RESEARCH PROCEDURE

Participants who discontinue prematurely continue to be followed as outlined in the protocol. Under no circumstances should the planned follow-up be altered.

The participant must receive the best possible care, taking into account their health status and the current state of medical knowledge.

- - 1. LOST PARTICIPANT TO FOLLOW UP

Un participant est considéré comme perdu de vue quand il arrête le suivi prévu dans le cadre du protocole sans raison connue de l’investigateur, de sorte que le recueil des données ne peut pas être effectué comme prévu.

Les participants perdus de vue doivent faire l’objet d’une recherche active de la part de l’investigateur.

- - 1. INCORRECTLY INCLUDED PARTICIPANT

A participant is considered incorrectly included if they do not meet all eligibility criteria. This must be discussed in the Scientific Council. They should continue follow-up until a decision is made.

**8.10. SIMULTANEOUS PARTICIPATION IN OTHER STUDIES, EXCLUSION PERIOD, COMPENSATION**

- Patients may participate simultaneously in another study, with no exclusion period.
- No compensation is provided.

# MANAGEMENT OF ADVERSE EVENTS, PREGNANCIES, AND NEW INFORMATION

## DEFINITIONS

**Adverse event (Article R1123-46 of the Public Health Code)**
Any harmful occurrence in a person participating in research involving human subjects, whether or not the event is related to the research or the product under investigation.

**Adverse effect (Article R1123-46 of the Public Health Code)**
An adverse event occurring in a person participating in research involving human subjects, when the event is related to the research or the product under investigation.

**Serious adverse event or effect (Article R1123-46 of the Public Health Code and ICH E2B guideline)**
Any adverse event or effect that:

- results in death,
- is life-threatening for the participant,
- requires hospitalization or prolongation of existing hospitalization,
- results in significant or permanent disability,
- causes a congenital anomaly or birth defect,
- or any event considered medically significant.

This applies to drugs regardless of the administered dose.
The expression “life-threatening” refers to an immediate risk to life at the time of the adverse event.

**Unexpected adverse effect for non-drug studies (Article R1123-46 of the Public Health Code)**
Any adverse effect whose nature, severity, or progression does not correspond with information regarding the products, procedures, or methods used during the research.

**New information (Article R1123-46 of the Public Health Code)**

- Any new data that may lead to a reassessment of the benefit-risk balance of the research or the product under investigation, to modifications in the use of this product, in the conduct of the research, or in the research-related documents, or to suspension, interruption, or modification of the protocol of the research or similar studies.
- For trials involving the first administration or use of a health product in individuals without any medical condition: any serious adverse effect.

## DESCRIPTION OF EXPECTED ADVERSE EVENTS

There are no adverse events specific to the imaging examinations performed under this protocol.
They are the same adverse events as for any MRI examination with contrast injection performed in routine clinical practice, as listed in the summary of product characteristics (SmPC) of the contrast agent used. Adverse events listed in the latest version of the SmPC (available at <https://base-donnees-publique.medicaments.gouv.fr/index.php>) are considered expected.

No adverse events are expected in relation to ultrasound.

Any adverse event not listed among the expected events or in the latest reference document for the contrast agent used is considered unexpected.

## 9.3. INVESTIGATOR PROCEDURE IN CASE OF ADVERSE EVENT, NEW INFORMATION, OR PREGNANCY

- - 1. COLLECTION OF ADVERSE EVENTS (AE)

The investigator is responsible for collecting adverse events occurring between the date of consent signature and the end of the participant’s study participation, i.e., no later than the day before surgery, once the participant has completed the research imaging examinations and is now under routine care. For this protocol, the investigator records clinical and biological adverse events of grade ≥3 (according to the CTCAE version 5.0) in the case report form. These adverse events are assessed at each study visit via participant interview and clinical examination.

**Exceptions to collection:**
The following circumstances do not need to be collected:

- Admission for social or administrative reasons,
- Hospitalization pre-defined by the protocol,
- Hospitalization for medical or surgical treatment planned before the research.

9.3.2. IMMEDIATE REPORTING OF SERIOUS ADVERSE EVENTS (SAE) AND NEW INFORMATION

The investigator evaluates each adverse event for severity and seriousness (CTCAE version 5.0).

The investigator must notify the Safety and Vigilance Unit by fax/email without delay from the day they become aware of any SAE or new information.
If the investigator becomes aware of an SAE suspected to be related to the research occurring after study completion in a participant they treated, they must notify the Safety and Vigilance Unit without delay.

The investigator must document the event as thoroughly as possible, providing the medical diagnosis if possible. Relevant follow-up information must be communicated to the Safety and Vigilance Unit as soon as possible.

In addition to the SAE notification form, the investigator must submit copies of laboratory results, examination reports, or hospitalization records relating to the SAE, including relevant negative results, anonymized and labeled with the participant’s number and code.

The investigator must follow the participant who experienced the SAE until resolution, stabilization at a medically acceptable level, or return to the prior state, even if the participant has withdrawn from the research. Additional information on event evolution, if not included in the initial report, must be sent to the Safety and Vigilance Unit.

The investigator and sponsor must independently assess causality between the SAE, experimental treatments, concomitant treatments, and the research.

All serious adverse events for which the investigator or sponsor considers a causal relationship reasonably possible are treated as suspected serious adverse effects.

The investigator must notify the sponsor of any new information they become aware of.

**Note:** In accordance with Articles R1413-67 et seq. and L1413-14, the sponsor reminds that any healthcare professional or legal representative of a health or medico-social institution who observes a nosocomial infection or a serious adverse event related to care must report it to the Regional Health Agency director. This reporting is independent of SAE reporting to the sponsor.

- - 1. PREGNANCY Notification

The occurrence of pregnancy during or immediately after the research does not constitute an SAE. However, if a woman becomes pregnant during the research, the pregnancy must be reported using the same procedure as for an SAE, as it will require specific follow-up until its outcome. The investigator informs the sponsor’s Safety and Vigilance Unit using the pregnancy reporting form. The investigator must follow the participant until the end of the pregnancy or its termination and report the outcome to the Safety and Vigilance Unit. Any abnormalities observed in the fetus or child must be reported. Any voluntary termination of pregnancy (VTP), medically indicated termination (MIT), or miscarriage must be reported, and if a seriousness criterion is present, it must be reported as an SAE.

- - 1. SUMMARY TABLE OF NOTIFICATION ROUTES BY TYPE OF EVENT

| **Type OF EVENT** | **NOTIFICATION PROCEDURE** | **NOTIFICATION TIMEFRAME TO THE SAFETY AND VIGILANCE UNIT** |
| --- | --- | --- |
| ADVERSE EVENT (AE) – non serious, grade **grade ≥3** | "AE" form in the case report form | No immediate notification |
| Serious Adverse EVent (SAE) | "AE" form in the case report form and Reporting Form | Immediate notification |

|  |  | Initial SAE report (+follow-up if necessary) + written report if necessary |  |  |
| --- | --- | --- | --- | --- |
|  | New finding | Written report | **Immediate notification** |  |
|  | Pregnancy | Pregnancy declaration form and case report form | **Upon confirmation of pregnancy** |  |

### Clinical Research Safety and Monitoring Unit CHU de Bordeaux

**Tél :** 05 57 82 16 26

**Fax :** 05 57 82 12 62

Courriel : [vigilance.essais-cliniques@chu-bordeaux.fr](mailto:vigilance.essais-cliniques@chu-bordeaux.fr)

**9.4 REPORTING OF UNEXPECTED SERIOUS ADVERSE EVENTS, NEW FINDINGS, AND OTHER EVENTS BY THE SPONSOR**

The safety and vigilance unit evaluates whether a serious adverse event is expected or unexpected based on the list of expected serious adverse events described in paragraph 9.2 of the protocol and the reference document as defined in the protocol.

The sponsor/safety unit reports safety information to the competent authorities and the Ethics Committee (CPP) according to the regulatory requirements specific to each type of trial.

The safety and vigilance unit reports to the competent authorities any suspicion of an unexpected serious adverse event that occurs within France and outside of the country, within the following timelines:

1. **In the case of an unexpected serious adverse event that led to death or life-threatening conditions**, without delay from the day the sponsor becomes aware of it,
2. **In the case of other unexpected serious adverse events**, no later than 15 days from the day the sponsor becomes aware of it.

The safety and vigilance unit submits a follow-up report to the ANSM (French National Agency for Medicines and Health Products Safety) with relevant additional information concerning unexpected serious adverse events:

- **In the case of suspected unexpected serious adverse events that led to death or life-threatening conditions**, this information must be reported within 8 days from the declaration mentioned in point 1),
- **In other cases of suspected unexpected serious adverse events and new findings**, relevant additional information must be provided within a new 8-day period from the deadline mentioned in point 2).

The safety and vigilance unit must report new findings that occur during the study:

- To the ANSM,
- To the Committee for the Protection of Persons (CPP).

The sponsor and investigator take appropriate urgent actions. The sponsor informs the competent authority and the Ethics Committee without delay.

**9.5 ANNUAL SAFETY REPORT**

On the anniversary of the first participant's inclusion, the safety and vigilance unit will prepare a safety report that includes:

- A list of serious adverse events that could be related to the experimental treatment(s) in the study, including both expected and unexpected serious adverse events that occurred during the period covered by the report,
- A concise and critical analysis of the safety of participants in the study,
- Summary tables of all serious adverse events that occurred in the trial since the start of the research.

This report is sent to the ANSM and the CPP within 60 days of the anniversary of the first inclusion.

**10 STATISTICAL ASPECTS**

**10.1 STUDY SIZE CALCULATION**

This is an exploratory proof-of-concept study with no similar bibliographic reference. The study size was empirically set at n = 50 patients. There is no prior statistical basis to establish a sample size. In SOPHiA Genetics studies, study sizes are set to 50 patients to identify a first signal.

**10.2 STATISTICAL METHODS USED**

After imaging acquisitions and a co-registration step, the following variables will be obtained for each region of interest (ROI):

- MR elastography**: μMRE

- DWI elastography**: ADCb200, ADCb1500, μDWI

- US elastography**: μUS

- CT scan**: d-, d+arterial, d+nephro, d+late phase

We will also have a set of potentially confounding variables related to the patient (age, gender, BMI, sarcopenia, GFR, height of each kidney, thickness of each kidney cortex, skin-to-measurement distance) and related to the lesion (size, volume, anterior/posterior location, upper/polar/equatorial/inferior, endophytic/exophytic) as well as the final diagnosis of the lesions: benign/malignant, histological type, and for RCC, histological grade.

All these variables will be described in terms of absolute numbers and percentages for categorical variables and as means, standard deviations, medians, minimum, maximum, and interquartile range for numerical variables.

* The correlations for each pair of imaging variables across all ROIs will be evaluated using the Spearman rank test.

* If applicable, the relationships between μDWI, μMRE, and μUS will be explored empirically (linear, logarithmic, quadratic, etc.).

* The repeatability of elasticity measurements will be evaluated using the Bland-Altman method and the intra-class correlation coefficient (ICC).

* Associations between μDWI, μMRE, μUS, potential confounding patient and lesion variables will be studied, as well as associations between μDWI, μMRE, μUS, and clinically relevant characteristics of renal lesions using Spearman tests (for pairs of numerical variables), Mann-Whitney tests (for pairs of categorical and numerical variables), and Chi-squared and Fisher tests (for pairs of categorical variables).

* The final step will involve developing a predictive model for y = μMRE based on the variables X = d-, d+arterial, d+nephro, d+late phase. Multiple observations per patient (renal lesion(s), healthy parenchyma) will be available. The patient and ROI type (healthy, tumoral) will be added as covariates in the model.

The sample will be split into a training set and a test set (70%/30% of the population). Several regression algorithms will be trained with repeated cross-validation (linear regression, penalized elasticnet linear regression, k-nearest neighbors, support vector machine, random forest, artificial neural network, etc.) and selected based on RMSE. The performance of the best models will then be independently evaluated on the test set (using nested cross-validation). Analyses will be conducted in collaboration between the SOPHiA Genetics team and Professor Crombé.

**10.3 STATISTICAL ANALYSIS PLAN**

A detailed statistical analysis plan will be defined and validated by the Scientific Advisory Board of the study.

**11 RESEARCH MONITORING**

11.1 SCIENTIFIC ADVISORY BOARD

11.1.1 COMPOSITION*

The board is composed of the following individuals: Dr. Eva JAMBON (Principal Investigator), Dr. Yann LE BRAS (Co-Investigator), Professor Jean-Christophe BERNHARD (Scientific Lead), Professor Amandine CROMBE (Co-Scientific Lead), Dr. Magalie CASTOREO (Pharmacist, Safety and Vigilance Unit), Professor Thierry COLIN (Methodology Center), Professor Laura RICHERT (Methodologist), Solène RICARD and Manon JAFFREDO (Project Leaders), Joséphine GAY (Clinical Research Associate supporting the investigator), Guillaume HERMAN (Data Manager), and a representative from the sponsor.

11.1.2 MEETING FREQUENCY

The Scientific Advisory Board meets once a year.

11.1.3 ROLES

* The Scientific Advisory Board is tasked with making any important decisions at the request of the coordinating investigator regarding the progress of the research and adherence to the protocol.

* It ensures compliance with ethical standards.

* It gathers information from the Methodology and Data Management Center and the coordinating investigator on the progress of the research, potential issues, and available results.

* It decides on any relevant protocol modifications necessary to continue the research, including:

* Measures to facilitate participant recruitment,

* Modifications to the protocol before they are presented to the CPP and the competent health authority,

* Decisions on opening or closing research sites,

* Measures ensuring the best safety for research participants (including modifications to information and consent documents),

* Discussions of results and publication strategy.

* The Scientific Advisory Board may propose (after consultation with the Independent Committee) to extend or interrupt the study in case of slow participant enrollment, a high number of lost to follow-up, major protocol violations, or for medical and/or administrative reasons. It will specify any conditions for the extended follow-up of participants included in the study.

* If new biological research is proposed using materials from the study and has not been previously planned in the protocol, the Scientific Advisory Board will assess it and define the conditions for participant information, data access, and result publication.

* After the meeting, the chair of the Scientific Advisory Board must inform the sponsor of the decisions taken. Major changes or modifications to the budget must be approved by the sponsor.

11.2 INDEPENDENT MONITORING COMMITTEE

The design of the study follows the patient's usual care, except for the addition of an ultrasound exam (which carries no risk) and MRI imaging with contrast agent injection (used within its marketing authorization, with minor risks as long as contraindications are respected). Therefore, we consider that the implementation of this research project does not justify the creation of an independent monitoring committee. However, such a committee may be established during the study if safety issues arise and/or upon decision by the investigators and/or the sponsor's vigilance unit.

**12 DATA MANAGEMENT AND SOURCE DOCUMENTS HANDLING**

**12.1 SOURCE DATA AND DOCUMENTS**

Source data refers to all the information contained in original documents or authenticated copies relating to clinical exams, observations, or other activities performed as part of a research project that are necessary for reconstructing and evaluating the study. The documents in which source data is recorded are called source documents.

The source documents used will include the electronic medical record of CHU of Bordeaux (DxCare®), the CHU of Bordeaux PACS imaging network, as well as dedicated data collection sheets and biological results on DxCare. They will also include imaging exams and their reports.

**12.2 DATA COLLECTION GUIDELINES**

All information required by the protocol must be recorded in the observation logs, with an explanation provided for any missing data.

Data should be collected as it becomes available and transcribed clearly and legibly.

Medical data will be collected in the UroCCR database.

**12.3 DATA MANAGEMENT AND FLOW**

12.3.1 DATA MANAGEMENT SOFTWARE

*12.3.1.1 SOFTWARE USED*

The software used for data management is an eCRF, accessible at the following address: [https://uroccr.fr](https://uroccr.fr/).
The maintenance and development of the eCRF are managed by CREDIM (Research and Development Center for Medical Informatics), a platform created at the University of Bordeaux.

*12.3.1.2 DATA HOSTING*

The data is stored on a dedicated database server managed by CREDIM. The database management system used is Microsoft SQL Server.

*12.3.1.3 DATA SECURITY*

The server is located in a dedicated room with no windows. Access to the secure room is granted using a badge. The service's doors are secured and locked at night. No computers are freely accessible; domain authentication is mandatory.

Access rights are managed by CREDIM for the service's studies.

Only database administrators, the project team, and auditors have direct access to the database.

Statisticians and data managers will have access to pseudonymized data transmitted via the secure NextCloud server managed by the DSIN (Department of Information Systems and Networks) at CHU Bordeaux.

*12.3.2 DATA ENTRY*

Data entry is the responsibility of the center’s investigator in the eCRF. Any other person entering data into the eCRF must be trained in advance and delegated by the investigator to do so.

*12.3.3 DATA CODING*

Prescribed treatments and clinical events are coded in the eCRF for the purpose of data monitoring and analysis.

The following dictionaries are used for medical term coding:

- MedDRA (current version) FR/US
- ATC version

The UroCCR investigative team is responsible for coding the data, under the investigator’s supervision.

*12.3.4 DATA MONITORING*

Monitoring is programmed to verify the consistency and completeness of the data entered into the eCRF. The list of required checks is defined jointly between the coordinating investigator and the statistical analysis manager in the study’s data validation plan.

The UroCCR data manager and the coordinating Clinical Research Associate (CRA) are responsible for managing correction requests, which are regularly initiated.

The investigator will make the necessary corrections to resolve the correction requests.

*12.3.5 RECONCILIATION OF SAEs/SAEs BASES*

If necessary, the reconciliation of databases is carried out by the Safety and Vigilance Unit (USV) of CHU Bordeaux according to the established procedure. The Data Manager is responsible for transmitting the adverse event table to the USV. The data is exported as an Excel file and uploaded to the secure exchange platform Nextcloud.

*12.3.6 DATA TRANSFER*

For security reasons, imaging and clinical data will be pseudonymized and hosted on the secure and controlled servers of CHU Bordeaux for the duration of the study. These data (imaging and relevant clinical data) may be transferred via the AZURE platform to SOPHiA Genetics for statistical analysis. The AZURE platform is certified HDS (Health Data Hosting).

*12.3.7 DATA ARCHIVING*

Data archiving is the responsibility of the study sponsor. The study data will remain stored on the CHU Bordeaux IT department's server, in compliance with the regulations in force for the study. A physical copy will be kept by the sponsor in accordance with current regulations.

**12.4 DATA CONFIDENTIALITY**

In compliance with applicable legal provisions, persons who have direct access to source data will take all necessary precautions to ensure the confidentiality of information related to experimental drugs, research activities, and participants, especially regarding their identity and obtained results. These individuals, as well as the investigators themselves, are bound by professional secrecy.

During or after the study, the data collected on participants and transmitted to the sponsor by the investigators (or any other specialized participants) will be pseudonymized. They must not reveal the participants' names or addresses.

Each participant will be assigned a confidential identification code consisting of a participant number (3 digits).

The sponsor will ensure that each participant has provided written consent for access to their individual data, which is strictly necessary for quality control purposes in the research.

**12.5 STORAGE OF RESEARCH DOCUMENTS AND DATA**

The following documents related to this research are archived by the investigator in accordance with Good Clinical Practice, the French decree of August 11, 2008, which sets the retention period for research documents in health, and the European regulation on medicinal products:

* For a period of 20 years after the end of the study:

* The protocol and any protocol amendments

* Observation logs (paper or electronic copies)

* Source documents of participants who have signed consent

* All other documents and correspondence related to the study

* The original signed informed consent forms from participants

These documents are under the investigator's responsibility for the regulatory retention period.

No relocation or destruction can take place without the sponsor’s consent. At the end of the regulatory retention period, the sponsor will be consulted for destruction. All data, documents, and reports may be subject to audit or inspection.

The data collected for the study will be accessible to authorized personnel at CHU Bordeaux for two years after the last publication of the research results. The data will be archived for 20 years after the study ends, in compliance with applicable regulations.

**12.6 DATA TRANSFER**

Data management is overseen by the urology team in collaboration with SOPHiA Genetics. The terms for transferring all or part of the research database are decided by the study sponsor and subject to a written contract.

**13. QUALITY CONTROL AND ASSURANCE**

13.3 ACCESS TO DATA

Acceptance of participation in the protocol implies that the investigators will make available the documents and individual data strictly necessary for monitoring, quality control, and audit of the research, to persons with access to these documents in accordance with the applicable legislative and regulatory provisions.

13.4 QUALITY CONTROL

Quality control will be carried out by a Clinical Research Associate (CRA) appointed by the sponsor, in accordance with the risk-based monitoring plan (participant, logistics, impact, resources) defined for the research.

The CRA will define the nature of the elements to be checked, as well as the procedures and frequency of site visits.

Each visit will result in a monitoring report, which will be transmitted to the principal investigator of the center.

13.5 AUDIT AND INSPECTION

An audit can be conducted at any time by individuals appointed by the sponsor, independent of those conducting the research. The purpose of the audit is to verify the safety of participants and the protection of their rights, compliance with applicable regulations, and the reliability of the data.

An inspection may also be carried out by a competent authority (e.g., ANSM in France or another regulatory authority for European research).

Both the audit and the inspection may apply at all stages of the research, from the protocol development to the publication of results and the filing of data used or produced during the research.

The investigators agree to comply with the sponsor's requirements regarding an audit and with the competent authority for an inspection of the research.

**14. ETHICAL AND REGULATORY CONSIDERATIONS**

**14.1 COMPLIANCE WITH REFERENCE TEXTS**

The sponsor and the investigator(s) commit to ensuring that this research is conducted in compliance with Law No. 2012-300 of March 5, 2012, related to research involving human subjects, as well as in accordance with Good Clinical Practices (ICH E6 (R2) of December 1, 2016, and the decision of November 24, 2006) and the Declaration of Helsinki (which can be found in its full version on the website [www.wma.net](http://www.wma.net)).

The research is conducted in accordance with this protocol. Except in emergency situations requiring specific therapeutic measures, the investigator(s) commit to adhering strictly to the protocol, particularly regarding obtaining informed consent and reporting and monitoring serious adverse events.

This research has received a favorable opinion from the Ethics Committee (CPP IdF1) and authorization from ANSM.

CHU Bordeaux, the sponsor of this research, has taken out a civil liability insurance contract with Lloyd’s Insurance Company SA (represented by BEAH, agent) in accordance with the provisions of the Public Health Code.

The data necessary for this research are recorded in the UroCCR database, which was authorized by the National Commission on Informatics and Liberty (CNIL) on April 12, 2013 (authorization request No. 912578, decision DR-2013-206). The data in the UroCCR database are processed electronically by CREDIM in compliance with Law No. 78-17 of January 6, 1978, related to data processing, files, and freedoms, as amended by Law 2004-801 of August 6, 2004.

This research is part of the "Reference Methodology" MR-001 in application of Article 54 of the amended Law of January 6, 1978, related to information, files, and freedoms. CHU Bordeaux has signed a commitment to comply with this "Reference Methodology."

This research is registered in the ID-RCB database under number 2024-A00959-38. The research is also registered on the site [http://clinicaltrials.gov/](http://clinicaltrials.gov/).

**14.2 PROTOCOL AMENDMENTS**

Any substantial modification, i.e., any modification likely to have a significant impact on the protection of individuals, the validity conditions and results of the research, the quality and safety of experimental products, the interpretation of scientific documents supporting the research, or on the methods of conducting the research, will be subject to a written amendment submitted to the sponsor. The sponsor must obtain, before implementation, a favorable opinion from the CPP and, if applicable, authorization from ANSM.

Non-substantial modifications, i.e., those having no significant impact on any aspect of the research, are communicated to the CPP for information.

All modifications are validated by the sponsor and all relevant research participants before submission to the CPP and, if necessary, to ANSM. This validation may require a meeting of the committee formed for the research.

All protocol modifications must be communicated to all investigators participating in the research. The investigators agree to comply with the content of the modifications.

Any modification affecting the management of participants or the benefits, risks, and constraints of the research will require a new information sheet and a new informed consent form, following the same procedure as previously described.

**15. FINAL REPORT**

A final report will be prepared and signed by the sponsor and the investigator within one year following the completion or termination of the research. This report will be made available to the competent authority. The sponsor will transmit to the CPP and, if necessary, to ANSM the research results in the form of a summary of the final report within one year after the end of the research.

**16. RULES REGARDING PUBLICATION**

**16.1 SCIENTIFIC COMMUNICATIONS**

The data analysis provided by the investigator center is conducted by SOPHiA Genetics. This analysis results in a written report that is submitted to the sponsor, who will then transmit it to the Ethics Committee and the competent authority.

Any written or oral communication of research results must receive prior approval from the coordinating investigator and, if necessary, from any committee formed for the research.

The coordinating investigator commits to making the research results publicly available, whether positive, negative, or inconclusive.

For the publication of the main results, the names of the sponsor, all investigators who included or followed participants in the research, methodologists, biostatisticians, and data managers involved in the research, members of any committees formed for the research, and SOPHiA Genetics’ participation, as well as the mention “This work received funding from the State managed by the National Research Agency under the third PIA integrated into France 2030, reference ANR-21-RHUS-0015,” must be acknowledged. International writing and publication rules (The Uniform Requirements for Manuscripts by ICMJE, April 2010) will be followed.

**16.2 COMMUNICATION OF RESULTS TO PARTICIPANTS**

In accordance with Law No. 2002-303 of March 4, 2002, participants will be informed, upon request, of the overall results of the research.

# BIBLIOGRAPHIC REFERENCES

1. Siegel RL, Miller KD, Fuchs HE, Jemal A. Cancer Statistics, 2021. CA Cancer J Clin. janv 2021;71(1):7‑33.
2. Escudier B, Porta C, Schmidinger M, Rioux-Leclercq N, Bex A, Khoo V, et al. Renal cell carcinoma: ESMO Clinical Practice Guidelines for diagnosis, treatment and follow-up†. Ann Oncol Off J Eur Soc Med Oncol. 1 mai 2019;30(5):706‑20.
3. Frank RA, Dawit H, Bossuyt PMM, Leeflang M, Flood TA, Breau RH, et al. Diagnostic Accuracy of MRI for Solid Renal Masses: A Systematic Review and Meta-analysis. J Magn Reson Imaging JMRI. avr 2023;57(4):1172‑84.
4. Schieda N, Davenport MS, Silverman SG, Bagga B, Barkmeier D, Blank Z, et al. Multicenter Evaluation of Multiparametric MRI Clear Cell Likelihood Scores in Solid Indeterminate Small Renal Masses. Radiology. juin 2022;303(3):590‑9.
5. Bauman TM, Potretzke AM, Wright AJ, Knight BA, Vetter JM, Figenshau RS. Partial Nephrectomy for Presumed Renal-Cell Carcinoma: Incidence, Predictors, and Perioperative Outcomes of Benign Lesions. J Endourol. avr 2017;31(4):412‑7.
6. Cornelis F, Tricaud E, Lasserre AS, Petitpierre F, Bernhard JC, Le Bras Y, et al. Routinely performed multiparametric magnetic resonance imaging helps to differentiate common subtypes of renal tumours. Eur Radiol. mai 2014;24(5):1068‑80.
7. Kay FU, Canvasser NE, Xi Y, Pinho DF, Costa DN, Diaz de Leon A, et al. Diagnostic Performance and Interreader Agreement of a Standardized MR Imaging Approach in the Prediction of Small Renal Mass Histology. Radiology. mai 2018;287(2):543‑53.
8. Vendrami CL, Velichko YS, Miller FH, Chatterjee A, Villavicencio CP, Yaghmai V, et al. Differentiation of Papillary Renal Cell Carcinoma Subtypes on MRI: Qualitative and Texture Analysis. AJR Am J Roentgenol. déc 2018;211(6):1234‑45.
9. Zhang J, Yu Y, Liu X, Tang X, Xu F, Zhang M, et al. Evaluation of Renal Fibrosis by Mapping Histology and Magnetic Resonance Imaging. Kidney Dis Basel Switz. mars 2021;7(2):131‑42.
10. Güven AT, Idilman IS, Cebrayilov C, Önal C, Kibar MÜ, Sağlam A, et al. Evaluation of renal fibrosis in various causes of glomerulonephritis by MR elastography: a clinicopathologic comparative analysis. Abdom Radiol N Y. janv 2022;47(1):288‑96.
11. Prezzi D, Neji R, Kelly-Morland C, Verma H, OʼBrien T, Challacombe B, et al. Characterization of Small Renal Tumors With Magnetic Resonance Elastography: A Feasibility Study. Invest Radiol. juin 2018;53(6):344‑51.
12. Rouvière O, Souchon R, Pagnoux G, Ménager JM, Chapelon JY. Magnetic resonance elastography of the kidneys: feasibility and reproducibility in young healthy adults. J Magn Reson Imaging JMRI. oct 2011;34(4):880‑6.
13. Le Bihan D, Ichikawa S, Motosugi U. Diffusion and Intravoxel Incoherent Motion MR Imaging-based Virtual Elastography: A Hypothesis-generating Study in the Liver. Radiology. nov 2017;285(2):609‑19.
14. Toffoli T, Saut O, Etchegaray C, Jambon E, Le Bras Y, Grenier N, et al. Differentiation of Small Clear Renal Cell Carcinoma and Oncocytoma through Magnetic Resonance Imaging-Based Radiomics Analysis: Toward the End of Percutaneous Biopsy. J Pers Med. 28 sept 2023;13(10):1444.
15. Cornelis F, Grenier N. Multiparametric Magnetic Resonance Imaging of Solid Renal Tumors: A Practical Algorithm. Semin Ultrasound CT MR. févr 2017;38(1):47‑58.
16. Cornelis F, Tricaud E, Lasserre AS, Petitpierre F, Bernhard JC, Le Bras Y, et al. Multiparametric magnetic resonance imaging for the differentiation of low and high grade clear cell renal carcinoma. Eur Radiol. janv 2015;25(1):24‑31.
