# Supplementary material for: Multimodal imaging to analyze the biomechanical properties of kidney tumors, evaluating feasibility, inter-modality correspondence, and diagnostic value (UroCCR-115)
Source: PLoS One. 2026 Jul 8;21(7):e0351477. doi: 10.1371/journal.pone.0351477 (PMC13345387; doi:10.1371/journal.pone.0351477)
Supplement: S4 File — (DOCX) [file pone.0351477.s004.docx]

**Associated content**

Jambon E, Courtine C, Soulabaille B, Chéchin D, Robert B, Sarrazin J, Margue G, Bernhard JC, Le Bras Y, Crombé A. Improving the Accuracy and Repeatability of Renal Stiffness Measurement With Magnetic Resonance Elastography: Influence of Vibration Frequency, Acquisition Orientation, and Postprocessing-A Phantom and Volunteer Study. NMR Biomed. 2025 Jun;38(6):e70054. doi: 10.1002/nbm.70054. PMID: 40387392; PMCID: PMC12087421.

Rubat Baleuri F, Pattou M, Jaffredo M, Lacroix G, Courtine C, Sarrazin J, Tamer M, Larribere H, Ricard S, Jambon E, Sabatier J, Bos F, Faessel M, Mondain-Monval O, Leng J, Bernhard JC, Margue G. From visualization to education: the role of 3D-Printed and virtual kidney models in training for renal cancer surgery, a systematic review. J Robot Surg. 2025 Dec 5;20(1):52. doi: 10.1007/s11701-025-02961-7. PMID: 41345321.
