# Supplementary material for: Multimodal imaging to analyze the biomechanical properties of kidney tumors, evaluating feasibility, inter-modality correspondence, and diagnostic value (UroCCR-115)
Source: PLoS One. 2026 Jul 8;21(7):e0351477. doi: 10.1371/journal.pone.0351477 (PMC13345387; doi:10.1371/journal.pone.0351477)
Supplement: Peer review history

# Editor report: Decision Letter 0

**DOI:** 10.1371/journal.pone.0351477.r001

**Authors:** Jennifer Tucker

27 May 2026

MECHANICS PROTOCOL

Multimodal imaging to analyze the biomechanical properties of kidney tumors, evaluating feasibility, inter-modality correspondence, and diagnostic value (UroCCR-115).

PONE-D-26-10721

Dear Dr. Fourage,

We’re pleased to inform you that your manuscript has been judged scientifically suitable for publication and will be formally accepted for publication once it meets all outstanding technical requirements.

Within one week, you’ll receive an e-mail detailing the required amendments. When these have been addressed, you’ll receive a formal acceptance letter and your manuscript will be scheduled for publication.

An invoice will be generated when your article is formally accepted. Please note, if your institution has a publishing partnership with PLOS and your article meets the relevant criteria, all or part of your publication costs will be covered. Please make sure your user information is up-to-date by logging into Editorial Manager at Editorial Manager® and clicking the ‘Update My Information' link at the top of the page. For questions related to billing, please contact billing support.

If your institution or institutions have a press office, please notify them about your upcoming paper to help maximize its impact. If they’ll be preparing press materials, please inform our press team as soon as possible -- no later than 48 hours after receiving the formal acceptance. Your manuscript will remain under strict press embargo until 2 pm Eastern Time on the date of publication. For more information, please contact onepress@plos.org.

Kind regards,

Jennifer Tucker, PhD

Staff Editor

PLOS One

Journal requirements:

1.  Thank you for stating in your Funding Statement:

“This work was supported by the Digital Urology 3D project (www.digital-urology-3d.fr) through a government independant grant managed by the French National Research Agency under the third Investment Program for the Future, as part of France 2030 (reference ANR-21-RHUS-0015). Additional institutional support is provided by the University Hospital of Bordeaux (CHU Bordeaux). “

Please provide an amended statement that declares *all* the funding or sources of support (whether external or internal to your organization) received during this study, as detailed online in our guide for authors at http://journals.plos.org/plosone/s/submit-now.  Please also include the statement “There was no additional external funding received for this study.” in your updated Funding Statement.

Please respond by return e-mail so that we can amend your financial disclosure and competing interests on your behalf.

2. Thank you for stating the following financial disclosure:

“This work was supported by the Digital Urology 3D project (www.digital-urology-3d.fr) through a government independant grant managed by the French National Research Agency under the third Investment Program for the Future, as part of France 2030 (reference ANR-21-RHUS-0015). Additional institutional support is provided by the University Hospital of Bordeaux (CHU Bordeaux). “

Please state what role the funders took in the study.  If the funders had no role, please state: "The funders had no role in study design, data collection and analysis, decision to publish, or preparation of the manuscript."

If this statement is not correct you must amend it as needed.

Please respond by return e-mail so that we can amend your financial disclosure and competing interests on your behalf.

3. Your ethics statement should only appear in the Methods section of your manuscript. If your ethics statement is written in any section besides the Methods, please move it to the Methods section and delete it from any other section. Please ensure that your ethics statement is included in your manuscript, as the ethics statement entered into the online submission form will not be published alongside your manuscript.

Additional Editor Comments (optional):

We are pleased to accept your Study Protocol for publication following expedited review, and hope you may consider publishing the study findings with PLOS in the future.

Reviewers' comments:

# Editor report: Acceptance letter

**DOI:** 10.1371/journal.pone.0351477.r002

**Authors:** Jennifer Tucker

PONE-D-26-10721

PLOS One

Dear Dr. Fourage,

I'm pleased to inform you that your manuscript has been deemed suitable for publication in PLOS One. Congratulations! Your manuscript is now being handed over to our production team.

At this stage, our production department will prepare your paper for publication. This includes ensuring the following:

* All references, tables, and figures are properly cited

* All relevant supporting information is included in the manuscript submission,

* There are no issues that prevent the paper from being properly typeset

You will receive further instructions from the production team, including instructions on how to review your proof when it is ready. Please keep in mind that we are working through a large volume of accepted articles, so please give us a few days to review your paper and let you know the next and final steps.

Lastly, if your institution or institutions have a press office, please let them know about your upcoming paper now to help maximize its impact. If they'll be preparing press materials, please inform our press team within the next 48 hours. Your manuscript will remain under strict press embargo until 2 pm Eastern Time on the date of publication. For more information, please contact onepress@plos.org.

You will receive an invoice from PLOS for your publication fee after your manuscript has reached the completed accept phase. If you receive an email requesting payment before acceptance or for any other service, this may be a phishing scheme. Learn how to identify phishing emails and protect your accounts at https://explore.plos.org/phishing.

If we can help with anything else, please email us at customercare@plos.org.

Thank you for submitting your work to PLOS ONE and supporting open access.

Kind regards,

PLOS ONE Editorial Office Staff

on behalf of

Dr Jennifer Tucker

Staff Editor

PLOS One
